# *TP53* and *OSBPL10* alterations in diffuse large B-cell lymphoma: prognostic markers identified via exome analysis of cases with extreme prognosis

**DOI:** 10.18632/oncotarget.24656

**Published:** 2018-04-13

**Authors:** Akito Dobashi, Yuki Togashi, Norio Tanaka, Masahiro Yokoyama, Naoko Tsuyama, Satoko Baba, Seiichi Mori, Kiyohiko Hatake, Toshiharu Yamaguchi, Tetsuo Noda, Kengo Takeuchi

**Affiliations:** ^1^ Pathology Project for Molecular Targets, The Cancer Institute, Japanese Foundation for Cancer Research, Koto, Tokyo, Japan; ^2^ Division of Pathology, The Cancer Institute, Japanese Foundation for Cancer Research, Koto, Tokyo, Japan; ^3^ The Cancer Precision Medicine Center, Japanese Foundation for Cancer Research, Koto, Tokyo, Japan; ^4^ Department of Hematology and Oncology, The Cancer Institute Hospital, Japanese Foundation for Cancer Research, Koto, Tokyo, Japan

**Keywords:** TP53, OSBPL10, diffuse large B-cell lymphoma, next-generation sequencing, prognostic marker

## Abstract

Diffuse large B-cell lymphoma (DLBCL) is the most common lymphoma subtype characterized by both biological and clinical heterogeneity. In refractory cases, complete response/complete response unconfirmed rates in salvage therapy remain low. We performed whole-exome sequencing of DLBCL in a discovery cohort comprising 26 good and nine poor prognosis cases. After candidate genes were identified, prognoses were examined in 85 individuals in the DLBCL validation cohort. In the discovery cohort, five patients in the poor prognosis group harbored both a *TP53* mutation and 17p deletion. Sixteen mutations were identified in *OSBPL10* in nine patients in the good prognosis group, but none in the poor prognosis group. In the validation cohort, *TP53* mutations and *TP53* deletions were confirmed to be poor prognostic factors for overall survival (OS) (P = 0.016) and progression-free survival (PFS) (P = 0.023) only when both aberrations co-existed. *OSBPL10* mutations were validated as prognostic markers for excellent OS (P = 0.037) and PFS (P = 0.041). Significant differences in OS and PFS were observed when patients were stratified into three groups—*OSBPL10* mutation (best prognosis), the coexistence of both *TP53* mutation and *TP53* deletion (poorest prognosis), and others. In this study, the presence of both *TP53* mutation and 17p/*TP53* deletion, but not the individual variants, was associated with poor prognosis in DLBCL patients after treatment with rituximab, cyclophosphamide, doxorubicin, vincristine and prednisone (R-CHOP) or similar regimens. We also identified *OSBPL10* mutation as a marker for patients with excellent prognosis in the R-CHOP era.

## INTRODUCTION

Diffuse large B-cell lymphoma (DLBCL) is the most common lymphoma that accounts for 30%-50% of lymphoma cases and is characterized by both biological and clinical heterogeneity. Rituximab-added CHOP chemotherapy (cyclophosphamide, doxorubicin, vincristine, and prednisolone) has improved the long-term outcomes of DLBCL with low clinical risk factors [6-year overall survival (OS): 90.1%; 6-year progression-free survival (PFS): 80.2%] [1]. However, in refractory cases, complete response or complete response unconfirmed (CR/CRu) rate in salvage therapy was only 38%, and the 3-year event-free survival (EFS) was 31% [2].

Gene expression profiling was first introduced in 2000 as a tool for the stratification of DLBCL [3]. DLBCL was classified into two subgroups, which were then designated as germinal center B cell (GCB)-like and activated B cell (ABC)-like subgroups, which were used to define prognostic categories, with ABC-like subgroups showing poorer prognoses [3]. Since then, considerable efforts have directed the stratification of DLBCL, based on mutation profiling via next-generation sequencing, [4–8] although somatic mutations detected in these studies matched only in 10-20% reflecting the genetic diversity of DLBCL [9]. Several somatic mutations have been reported as prognostic factors for DLBCL after treatment with R-CHOP or similar regimens. In a previous study, targeted capture sequencing of selected 34 genes in 215 DLBCL patients revealed that *TNFAIP3* and *GNA13* mutations were significantly associated with poorer prognosis in ABC-like DLBCL patients subjected to R-CHOP treatment [10]. Whole-exome sequencing (WES) of 14 relapsed/refractory large B-cell lymphoma patients (nine DLBCL and five primary mediastinal large B-cell lymphoma) identified several frequently altered genes in the cohort; however, non-relapsed/refractory cases were not sequenced for comparison [11]. Morin et al. performed WES of 38 relapsed/refractory DLBCL and detected *TP53*, *FOXO1*, *KMT2C*, *CCND3*, *NFKBIZ*, and *STAT6* as top candidate genes in which mutations were related to treatment resistance [12]. In Korea, six refractory DLBCL patients and seven responsive DLBCL patients were analyzed via WES and transcriptome sequencing [13]. Missense mutations in *TP53* were observed exclusively in refractory patients (3/6), and *TP53* copy number deletions were also detected in the same three patients [13]. A Chinese group reported the results of targeted capture sequencing of 27 genes in 196 DLBCL patients. Mutations or copy number deletions of *CD58* and *TP53* were found to be poor prognostic factors in their cohort [14].

Herein, we report alterations in *TP53* (a combination of point mutation and gene deletion) and *OSBPL10* (point mutation) as prognostic indicators for DLBCL. These indicators were identified via WES of 35 samples from DLBCL patients with extremely poor or excellent prognosis upon treatment with R-CHOP or similar regimens. Results were validated in an additional 85 cases as independent prognostic factors from the International Prognostic Index (IPI) for OS and PFS.

## RESULTS

### Whole-exome sequencing in the discovery cohort

**Figure 1:**
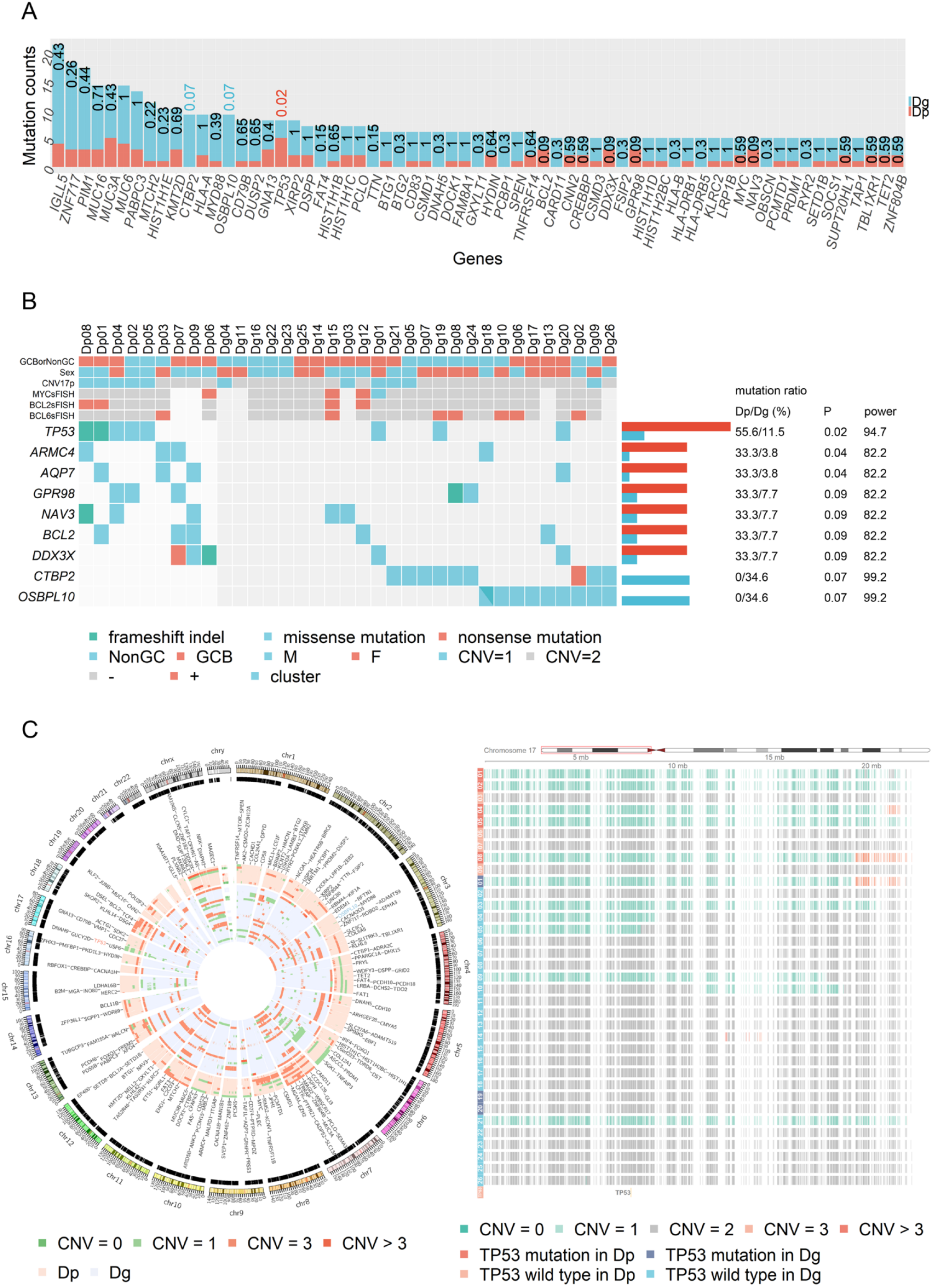
Mutational landscape and copy number variation in the discovery cohort **(A)** The numbers of cases with mutations stratified based on prognostic group in the discovery cohort are presented. Numbers above each bar represent *P*-values for Fisher’s exact test. All detected mutations before manual inspection are listed. **(B)** Genes that showed different mutations between the positive and poor prognostic groups are shown in each case of the cohort. Statistical power was calculated based on the method reported by Lawrence et al. [44] **(C)** The CIRCOS plot of copy number variation in the discovery cohort. The figure on the right is an enlarged view of 17p. Six of eight (75%) *TP53* mutations and six of 11 (55%) 17p deletions were found to coexist in the discovery cohort.

Clinical features and pathological characteristics of the discovery cohort are summarized in Table 1 and Supplementary Table 1. Significant differences in two IPI items (LDH and extranodal lesion) were found between groups with poor prognosis (Dp) and those with good prognosis (Dg) in the discovery cohort (Table 1). All double expressor cases (MYC >60% and BCL2 score 3+ [15]) were found in the poor prognosis group (Dp) (Table 1). WES was performed on 35 matched tumor-normal DNA (nine and 26 patients with poor and good prognoses, respectively). The average estimated tumor content was 56.47% (30.98 - 89.16%) (Supplementary Table 2). In both prognostic groups, CT/GA transversions were the most frequent variants, followed by AG/TC transversions; other mutations were relatively infrequent (Supplementary Figure 1A, 1B, and 1C). Mutations as triplets, XCG XTG/CGX CAX, were frequently observed (Supplementary Figure 1D). Somatic mutations filtered through pipeline are shown in Figure 1A and Supplementary Table 3.

**Table 1:** Comparison of characteristics between the patients with and without *TP53* or *OSBPL10* aberrations

		Discovery cohort			Validation cohort						
		Dp	Dg	*P*	V	*TP53* M + D	*TP53* W, M or D	*P*	*OSBPL10* M	*OSBPL10* W	*P*
*N*		9	26		85	6	79		21	64	
age		63.33 ± 3.77	60.58 ± 2.51	0.55	66.64 ± 1.37	71.50 ± 5.33	66.27 ± 1.42	0.38	65.24 ± 3.39	67.09 ± 1.46	0.62
sex	male	7	12		46	4	42		13	33	
	female	2	14	0.14	39	2	37	0.68	8	31	0.46
IPI	low	1	15		30	0	30		10	20	
	low intermediate	2	6		20	2	18		3	17	
	high intermediate	2	4		25	3	22		5	20	
	high	4	1	0.008	10	1	9	0.16	3	7	0.43
Clinical stage	I/II	2	16		47	3	44		11	36	
	III/IV	7	10	0.06	38	3	35	1	10	28	0.80
LDH	normal	1	19		43	1	42		11	32	
	high	8	7	0.002	42	5	37	0.11	10	32	1
ECOG-PS	0, 1	7	26		72	2	70		20	52	
	2, 3, 4	2	0	0.06	13	4	9	0.004	1	12	0.17
Extranodal lesion	<2	4	22		63	4	59		16	47	
	≥2	5	4	0.03	22	2	20	0.65	5	17	1
Hans algorithm	GCB	6	12		35	2	33		9	26	
	Non-GCB	3	14	0.44	42	4	38	0.68	12	30	0.80
Double expressor	negative	3	20		58	5	53		16	42	
	positive	2	0	0.03	8	0	8	1	0	8	0.18
CD5 (IHC)	negative	8	19		68	5	63		21	47	
	positive	1	6	0.64	9	1	8	0.54	0	9	0.10
*MYC* split FISH	negative	6	18								
	positive	1	3	1							
*BCL2* split FISH	negative	5	19								
	positive	2	2	0.25							
*BCL6* split FISH	negative	6	15								
	positive	1	6	0.64							

Double expressor: MYC >60% and BCL2 score 3+.

Dp: poor prognosis in the discovery cohort, Dg: good prognosis in the discovery cohort, V: Validation cohort, M: mutation, D: deletion, W: wild type, IHC: immunohistochemistry.

*TP53*, *CTBP2*, and *OSBPL10* alterations were selected as candidate prognostic factors based on the following criteria, *P* < 0.1 and statistical power > 90 (Figure 1B). However, *CTBP2* was discarded after manual inspection with Integrative Genomics Viewer (IGV) [16], because multiple mutations were detected from a single read of *CTBP2* in both tumor and normal samples, probably due to mapping error (Supplementary Figure 2). Therefore, only *TP53* and *OSBPL10* mutations were further verified by Sanger sequencing (data not shown).

**Figure 2:**
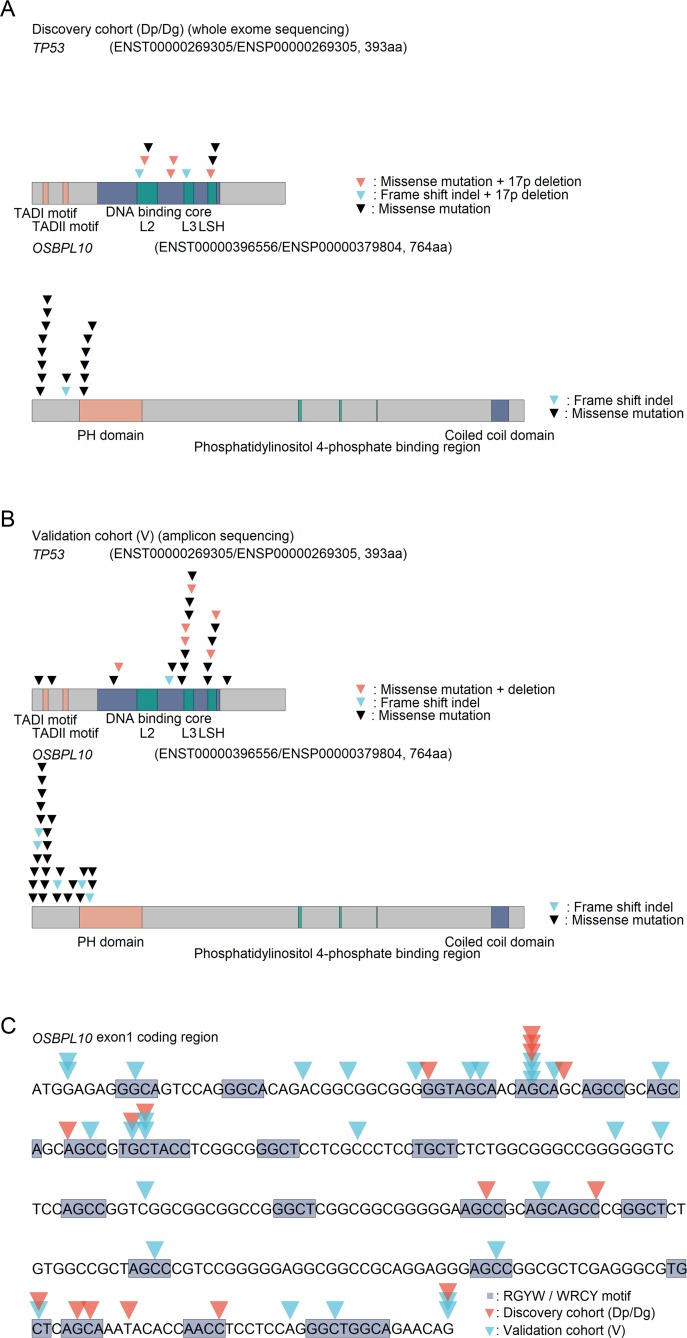
*TP53* and *OSBPL10* mutations in DLBCL **(A)** Graphical view of *TP53* and *OSBPL10* mutations in the discovery cohort. **(B)** Graphical view of the *TP53* and *OSBPL10* mutations in the validation cohort. **(C)** Overview of detected mutations in the *OSBPL10* exon 1 coding region.

*TP53* mutation sites were limited to the DNA-binding core domain (Figure 2A). Interestingly, in the poor prognostic group, five patients (Dp01, Dp02, Dp04, Dp05, and Dp08) harbored both *TP53* mutations and 17p deletion, and the remaining four patients did not have either one. By contrast, in the good prognostic group, only one (Dg01) had the both aberrations, although *TP53* mutations were detected in three patients and 17p deletion was detected in seven patients (Figure 1B, and 1C). Notably, the *TP53* mutation and 17p deletion were found to be poor prognostic factors for OS (*P* = 0.00035) and PFS (*P* = 0.013) only when patients had both aberrations (Supplementary Figure 3A).

A total of 16 mutations were identified in the *OSBPL10* genes of nine patients. Interestingly, all identified mutations were confined to the exon 1 coding region (Figure 2A), and all patients harboring the mutations belonged to the good prognostic group. *OSBPL10* mutations were found to be a highly reliable prognostic factor for improved PFS (*P* = 0.024) (Supplementary Figure 3B).

### *TP53* and *OSBPL10* aberrations in the validation cohort

On the basis of the results obtained from WES of the discovery cohort, we further analyzed another 85 DLBCL cases (validation cohort). Clinical features and pathological characteristics are summarized in Table 1 and Supplementary Table 1. Mutations in the whole coding regions of *TP53* and *OSBPL10* exon 1 were examined via amplicon sequencing. The average read counts and mean coverage were 650,800 (284,998 - 1,529,726) and 30,258 (12,414 - 71,264), respectively. Twenty-two *TP53* and 29 *OSBPL10* mutations were detected in 18 (21%) and 21 (25%) of the 85 patients, respectively (Supplementary Table 4).

*TP53* copy number loss (*TP53* deletion) was identified in 13 out of the 85 patients via real-time quantitative genomic PCR analysis (Supplementary Table 5). Six patients (V51, V67, V76, V77, V80, and V83) harbored both *TP53* mutation and deletion, and most mutations were confined to the DNA-binding core domain (Figure 2B). Although three patients had both *TP53* and *OSBPL10* mutations (V14, V25, and V31), none harbored all the three aberrations.

### *OSBPL10 in silico* functional prediction

*OSBPL10* mutations detected in our cohort were annotated based on protein functional prediction score (SIFT score [17] and Polyphen2 score [18]). Among the mutations that could be analyzed, 38.9% (7/18 cases) were classified as “deleterious” based on SIFT score and 30% (6/20 cases) were “possibly damaging” or “probably damaging” based on Polyphen2 score. On the other hand, 88.9% (16/18 cases) of *TP53* mutations were determined to be “deleterious” based on SIFT score, and 94.1% (16/17 cases) were analyzed as “probably damaging” based on Polyphen2 score (Supplementary Table 4).

### *OSBPL10* as a target of somatic hypermutation

**Figure 3:**
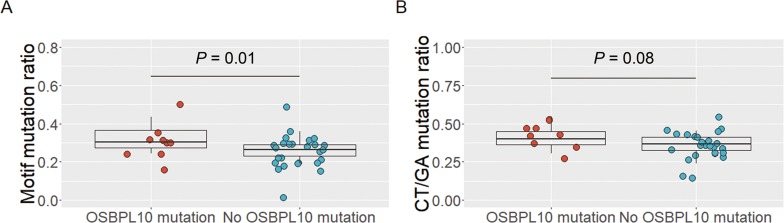
Analysis of *OSBPL10* mutations and somatic hypermutation target motifs **(A)** Proportion of RGYW/WRCY motif mutations to all somatic mutations. **(B)** Proportion of CT/GA mutations to all somatic mutations.

Interestingly, 30 out of the 45 *OSBPL10* mutations (67%) were located in the RGYW/WRCY motif (Figure 2C), which is known as a region susceptible to somatic hyper mutation (SHM), a mechanism that causes highly frequent somatic mutations in normal and neoplastic B cells [19]. In the discovery cohort, the proportion of motif mutations to all somatic mutations was significantly higher in individuals harboring *OSBPL10* mutations (32.6% vs. 26.2%; *P* = 0.01) (Figure 3A). The proportion of CT/GA mutations to all somatic mutations tended to be higher in individuals with *OSBPL10* mutations (*P* = 0.08) (Figure 3B). Furthermore, *OSBPL10* was identified as a SHM target based on the method reported by Khodabakhshi et al [20]. (Table 2, and Supplementary Table 6).

**Table 2:** *OSBPL10* was identified as a SHM target

Gene	Total SNVs	Motif mutation	Transition mutation	C:G mutation	SHM indicator
PIM1^*^	588	426	406	583	< 0.001
*IGLL5*	482	359	254	429	< 0.001
*PABPC3*	386	69	276	151	< 0.001
*KCNJ18*	214	0	102	214	< 0.001
*CTBP2*	209	31	93	111	0.0025
*ZNF717*	161	14	89	135	0.0018
*MUC3A*	161	25	75	76	0.0068
*ATAD3B*	120	0	120	120	< 0.001
*MUC6*	110	20	23	98	< 0.001
*LDHAL6B*	108	27	84	30	0.0073
*MTCH2*	107	3	81	32	< 0.001
*CD79B*	90	4	63	7	< 0.001
*PABPC1*	88	6	39	24	< 0.001
*CDC27*	76	30	66	0	< 0.001
*HLA-DRB1*	74	37	32	35	0.0408
*MYD88*	72	4	68	8	< 0.001
BTG1^*^	71	53	54	67	0.0024
DUSP2^*^	71	42	57	62	0.0273
*ANKLE1*	68	0	0	8	< 0.001
*AK2*	63	52	32	61	0.0013
*SHANK3*	61	21	61	17	0.0176
*AQP7*	58	1	32	57	0.0060
*CNN2*	43	31	39	24	0.0232
*MPEG1*	40	28	31	40	0.0307
*HNRNPL*	39	0	36	0	< 0.001
*OSBPL10*	30	28	22	27	0.0175
*KLRC2*	29	25	4	25	0.0039
*ARMC4*	28	8	0	20	0.0109
*FAM205A*	28	18	28	9	0.0387

^*^: previously reported gene.

### Prognostic values of *TP53* and *OSBPL10* aberrations

**Figure 4:**
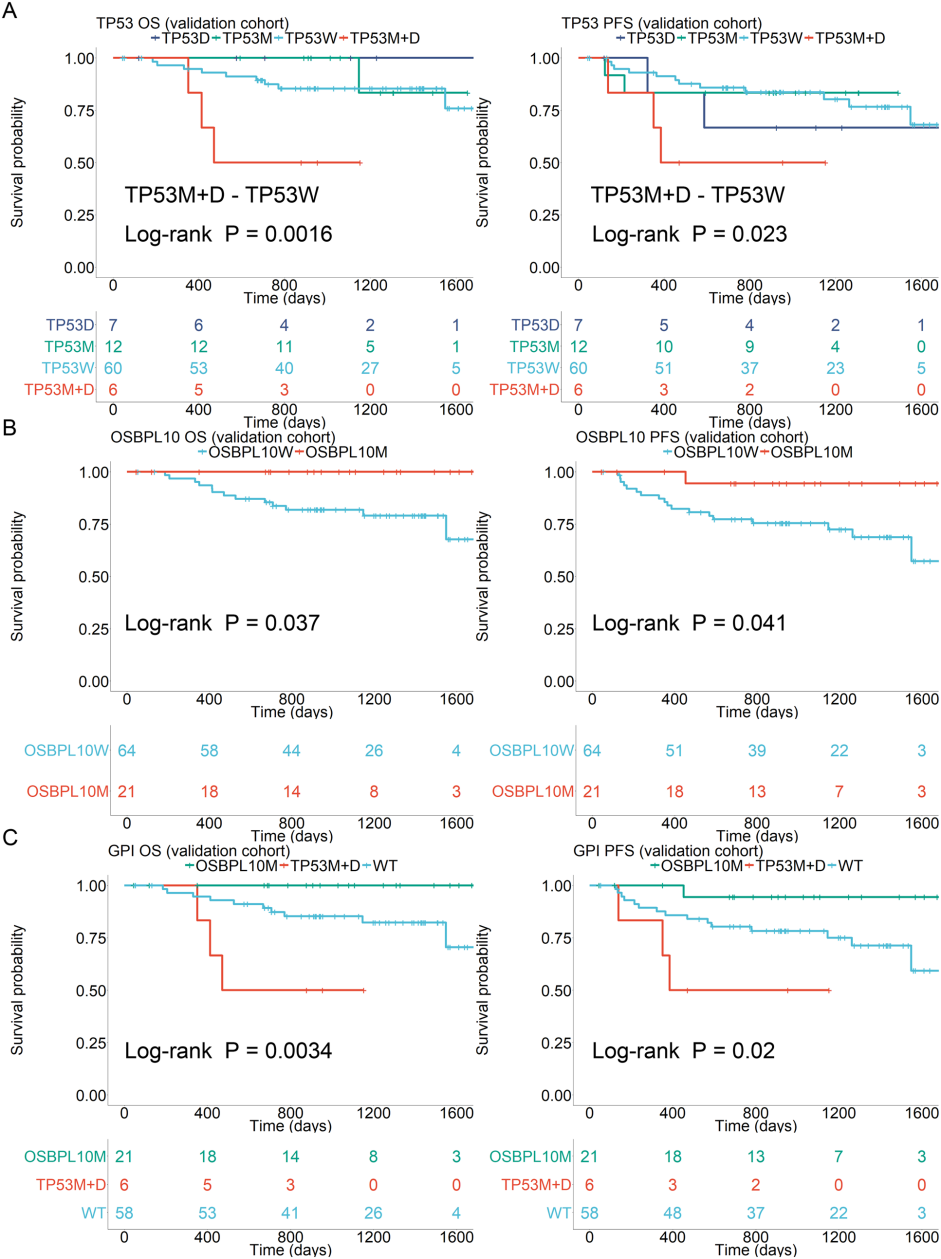
Survival analyses stratified by *TP53* and *OSBPL10* aberrations **(A)** Survival stratified by *TP53* status in the validation cohort. **(B)** Survival stratified by *OSBPL10* status in the validation cohort. **(C)** Survival stratified by Genetic Prognostic Index (GPI) in the validation cohort. TP53W: *TP53* wild-type; TP53M: *TP53* mutation; 17pD: 17p deletion; OSBPL10W: *OSBPL10* wild-type; and OSBPL10M: *OSBPL10* mutation.

In the validation cohort, as well as the discovery cohort, *TP53* mutations and deletions were found to be poor prognostic factors for OS (*P* = 0.0016) and PFS (*P* = 0.023) only when they co-existed (Figure 4A). *OSBPL10* mutation was validated as a highly reliable prognostic factor for better OS (*P* = 0.037) and PFS (*P* = 0.041) (Figure 4B). Significant differences were observed in OS and PFS when patients were stratified into three groups based on the presence of an *OSBPL10* mutation (best prognosis) and coexistence of both *TP53* mutation, deletion (poorest prognosis) (Figure 4C) and the others. Resulting values were designated as Genomic Prognostic Index (GPI).

In other clinicopathological factors listed in Table 1 and Supplementary Table 1, patients harboring both *TP53* mutations and deletions showed significantly lower ECOG performance status (ECOG-PS) (*P* = 0.004). Therefore, we applied the IPW method [21] to reduce the effects of IPI factors and conflicting gene mutations. Patients harboring both *TP53* mutation and deletion still had significantly poorer OS (*P* < 0.01) and PFS (*P* < 0.01) (Supplementary Figure 4A). The presence of both *TP53* mutation and deletion was found to be an independent poor prognostic factor from IPI in OS and PFS. Patients harboring *OSBPL10* mutations showed extremely good prognoses and tended to have better OS (*P* = 0.05) and PFS (*P* = 0.05) after applying the IPW method (Supplementary Figure 4B).

## DISCUSSION

In the present study, we showed that *TP53* mutation and 17p/*TP53* deletion were poor prognostic factors for OS and PFS in DLBCL patients treated with R-CHOP or similar regimens only when both aberrations were present. It is interesting to clarify whether the poor prognosis of patients harboring both aberrations is caused by loss of TP53 function alone or is augmented by deletions in other genes in the 17p region. Liu et al. suggested that the selective advantage of tumors is produced by the combined effects of *TP53* loss and the reduced levels of tumor suppressor genes linked to 17p deletion [22]. They reported that acute myeloid leukemia (AML) patients harboring both *TP53* mutation and 17p deletion showed a significantly poorer prognosis than patients with only one of these two genetic aberrations [22]. Liu et al. also demonstrated that heterozygous deletion of mouse chromosome 11B3, which corresponds to human 17p13.1, resulted in more aggressive lymphoma and leukemia than that produced by *Trp53* deletion because of the combined effect of *Trp53* loss and co-deletion of tumor suppressor genes in 11B3 [22]. The poor prognoses observed in our patients and the three refractory patients in a previous Korean study [13] who harbored both *TP53* mutation and 17p/*TP53* deletion were consistent with the observations reported by Liu et al. Meanwhile, no patients with biallelic 17p/*TP53* deletions were detected in the present study. Biallelic deletion (complete loss) of some genes in 17p may be lethal to lymphoma cells.

The relationship between *TP53* status and prognosis in DLBCL patients has been previously reported in 11 studies written in English (Table 3) [14, 23–32]. *TP53* mutation and 17p/*TP53* deletion showed variable prognostic impacts on DLBCL, although both tended to be poor prognostic factors. Notably, *TP53* mutations and deletions tend to coexist, and *TP53* deletion is frequently associated with 17p deletion, which frequently involves all or most of the chromosomal arm [22]. Accordingly, in the present study, 75% (6/8) of *TP53* mutations and 55% (6/11) of 17p deletions coexisted in the discovery cohort (Figure 1C), and 33% (6/18) of *TP53* mutations and 46% (6/13) of *TP53* deletions coexisted in the validation cohort. This pattern could be the cause of the variable prognostic impacts of *TP53* status as reported in literature. Among the 11 studies (Table 3), four examined both *TP53* mutations and 17p/*TP53* deletions and reported the number of patients having both genetic aberrations. However, only one study conducted during the CHOP era analyzed the impact of the coexistence of *TP53* mutation and deletion; the presence of both aberrations, but not only one of them, was determined to be a poor prognostic factor for DLBCL [27]. Our study is the first to provide data demonstrating the prognostic impacts of the coexistence of *TP53* mutation and deletion during the R-CHOP era.

**Table 3:** Literature review of *TP53* variant and prognostic analysis in DLBCL

					*TP53* mutation		17p deletion/*TP53* loss		Mutation with17p deletion/*TP53* loss	
Treatment	Study	Year		Total cases	OS	PFS/DFS	OS	PFS/DFS	OS	PFS/DFS
CHOP era	Ichikawa A et al.	1997 [23]		102	poor					
	Stokke T et al.	2001 [24]		94			poor			
	Leroy K et al.	2002 [25]		69	poor					
	Young KH et al.	2007 [26]		113	poor	NS	NS			
	Stöcklein H et al.	2008 [27]		40	NS		NS		poor	
	Young KH et al.	2008 [28]		477	poor					
R-CHOP era	Xu-Monette ZY et al.	2012 [29]		506	poor	poor	NS	NS		
	Asmar F et al.	2014 [30]		62	poor					
	Fiskvik I et al.	2015 [31]		43			poor	poor		
	Cao Y et al.	2016 [14]		165	poor	poor	poor	poor		
	Zenz T et al.	2017 [32]		265	poor	poor				
	Present study	2017	120	35 (Discovery cohort)	NS^*^	NS^*^	NS^#^	NS^#^	poor	poor
				85 (Validation cohort)	NS^*^	NS^*^	NS^#^	NS^#^	poor	poor

NS: not significant, ^*^: *TP53* mutation only, ^#^: 17p deletion/*TP53* loss only.

In a previous study, *OSBPL10* mutation was reported in three out of nine primary central nervous system lymphomas (PCNSLs) and was indicated as a novel target gene of SHM in PCNSL [33]. The authors suggested that aberrant SHM had a major impact on PCNSL pathogenesis, but the clinical impacts of *OSBPL10* mutation were not discussed [33]. In the present study, we confirmed that *OSBPL10* is also a target gene of SHM in non-central nervous system DLBCL and identified *OSBPL10* mutation as a biomarker for DLBCL with excellent prognosis. SHM, through which multiple somatic mutations may be generated in a single gene, is an important mechanism underlying the pathogenesis of B-cell neoplasms. Some mutation analysis pipelines employ filtering steps that discard candidate mutations when they are detected with several other mutations in a single read. It should be noted that applying such filtering steps to genetic alteration studies in B-cell neoplasms can potentially disregard relevant mutations generated by aberrant SHM.

OSBPL10 is a member of a family of sterol and phosphoinositide binding proteins, which consist of oxysterol-binding proteins (OSBPs) and OSBP-related proteins (ORPs). The mechanisms underlying their function remain to be fully elucidated [34]. In one breast cancer study, *OSBPL10* mutations, which have a prevalence of 5.2%, have been suggested as potential drivers of mutations; however, the clinical impacts of *OSBPL10* mutations were not described [35]. It is unclear whether OSBPL10 and/or its mutants play functionally important roles in DLBCL. The biological significance of *OSBPL10* mutations remains to be clarified by further studies.

Results of our present study showed that the presence of both *TP53* mutation and 17p/*TP53* deletion is associated with poor prognosis in DLBCL patients treated with an R-CHOP-like regimen. We also identified *OSBPL10* mutations as biomarkers for excellent prognosis in DLBCL patients during the R-CHOP era. In the clinical setting, reduced-intensity treatments may be delivered to patients with excellent prognoses. Further validation studies on larger cohorts, particularly both Asian and non-Asian groups, is warranted.

## MATERIALS AND METHODS

### Case selection

We selected 35 DLBCL cases as part of the discovery cohort according to the following criteria: (1) individuals diagnosed between January 2006 and December 2011 in The Cancer Institute Hospital (Tokyo, Japan), (2) individuals for whom frozen tissues or extracted DNA from frozen or fresh tissues were available, and (3) individuals with extremely poor prognosis [stable disease (SD) or progressive disease (PD) after first treatment] or with excellent prognosis [progression-free survival during the observation period for at least 3 years (until November 2016)]. Extracted DNA from matched fresh bone marrow specimens without lymphoma infiltration were available from 33 out of 35 cases. For the remaining two cases (Dg24 and Dg25), peripheral blood samples were used as matched normal samples.

All cases (85 DLBCL) that met the following criteria were included in the validation cohort: (1) individuals diagnosed between January 2012 to December 2014 in The Cancer Institute Hospital, (2) individuals for whom frozen tissues or extracted DNA from frozen or fresh materials were available, and (3) individuals treated with R-CHOP-like regimen.

All specimens were examined by pathologists (N. Tsuyama and K. Takeuchi), and DLBCL diagnoses were made according to the 4^th^ edition of the WHO classification [36]. This study was approved by the institutional review board.

### Sequencing analysis

Screening for gene mutations was performed via WES, using a customized capture probe set based on SureSelect XT Human All Exon V5 (Agilent, Santa Clara, USA). Libraries was prepared with a SureSelect Target Enrichment kit (Agilent) and sequenced on a HiSeq 2000 instrument (Illumina, San Diego, USA). Whole coding regions of *TP53* and *OSBPL10* exon 1 were amplified using TruSeq Custom Amplicon Low Input Kit (Illumina) and sequenced on a MiSeq platform (Illumina). Primers used for PCR and direct sequencing are listed in Supplementary Table 7. *TP53* copy number variations (CNVs) were determined via real-time quantitative genomic PCR by the 2^−ΔΔC^_T_ method [37] and using *GAPDH* as a reference gene. The primers used for real-time quantitative PCR are listed in Supplementary Table 8 [38, 39].

### Whole-exome sequencing data analysis

Analysis was performed as previously described, with several modifications [40]. NHLBI Exome Sequencing Project (http://evs.gs.washington.edu/EVS/) and Integrative Japanese Genome Variation Database (iJGVD) (https://ijgvd.megabank.tohoku.ac.jp/) were additionally included in the mutation reference data. For analysis of mutation overview and somatic hypermutation (SHM) targets, only annotated variants that met all the following conditions were selected: variants located in coding regions; variants detected from 10 or more reads; and variants called as somatic variants by more than one analysis tool. For analysis of somatic mutations, annotated variants that met at least one of the following conditions were discarded: exonic synonymous single nucleotide variants (SNVs); variants registered in dbSNP version 131; frequently observed variants (≥ 5%) in 1000 Genomes Project; frequently observed variants (≥ 5%) in NHLBI Exome Sequencing Project esp6500siv2; frequently observed variants (≥ five samples) in HGVD; frequently observed variants (≥ 5%) in iJGVD; and somatic variants called by more than one analysis tool. Copy number variation (CNV) and tumor content analyses were performed using ExomeCNV [41]. CNV was plotted with CIRCOS version 0.69-2, [42] Gviz, [43] and R version 3.3.2.

### Amplicon sequencing data analysis

After quality control of sequence reads, read mapping on hg19 was performed following the same method used in WES. SNVs and indel calling was performed using GATK Haplotype Caller and MiSeq Reporter v2. Mutations called by either one of the tools were manually selected using IGV [16].

### Propensity score analysis

To reduce bias during patient selection, inverse probability weighting (IPW) using propensity score was performed to investigate the causality of genetic variation and clinical outcomes in the validation cohort. In *TP53* mutation analysis, the variables entered in the propensity score model were IPI items [age, clinical stage, lactate dehydrogenase (LDH), ECOG-PS, and extranodal lesion] and *OSBPL10* mutation; similarly, in *OSBPL10* mutation analysis, the IPI items in addition to the *TP53* mutation were included as variables in the propensity score model. Next, analysis of adjusted survival curves and log-rank test were performed based on the IPW method, using R version 3.3.2 and the IPWsurvival package (http://www.divat.fr/en/softwares/ipwsurvival).

### Statistical analyses of clinical data

The Mann-Whitney test, Student's *t*-test, Welch two-sample *t*-test, Fisher's exact test, and log-rank test were performed using R version 3.3.2, coin, survminer (version 0.3.1), and survival (version 2.38).

### Availability of data and materials

Data has been deposited at the DDBJ Japanese Genotype-phenotype Archive (https://www.ddbj.nig.ac.jp/jga) under the accession JGAS00000000087.

## SUPPLEMENTARY MATERIALS FIGURES AND TABLES





## Abbreviations

ABC: activated B cell
AML: acute myeloid, leukemia
CNV: copy number variation
CR: complete response
CRu: complete response unconfirmed
Dg: good prognosis in the discovery cohort
DLBCL: diffuse large, B-cell lymphoma
Dp: poor prognosis in the discovery cohort
ECOG-PS: ECOG performance status
EFS: event-free survival
GCB: germinal center B cell
GPI: genomic prognostic index
IGV: integrative genomics viewer
iJGVD: integrative Japanese genome variation database
IPI: international prognostic index
IPW: inverse probability weighting
LDH: lactate dehydrogenase
ORPs: OSBP-related proteins
OS: overall survival
OSBPs: oxysterol-binding proteins
PCNSLs: primary central nervous system lymphomas
PD: progressive disease
PFS: progression-free survival
R-CHOP: (rituximab cyclophosphamide doxorubicin vincristine, and prednisone)
SD: stable disease
SHM: somatic hypermutation
SNVs: single nucleotide variants
V: the validation cohort
WES: whole-exome sequencing
