# Supplementary material for: TP53 and OSBPL10 alterations in diffuse large B-cell lymphoma: prognostic markers identified via exome analysis of cases with extreme prognosis
Source: Oncotarget. 2018 Apr 13;9(28):19555–68. doi: 10.18632/oncotarget.24656 (PMC5929408; doi:10.18632/oncotarget.24656)
Supplement: Supplementary file 1 [file oncotarget-09-19555-s001.pdf]

TP53 and *OSBPL10* alterations in diffuse large B-cell lymphoma: prognostic markers identified via exome analysis of cases with extreme prognosis

SUPPLEMENTARY MATERIALS

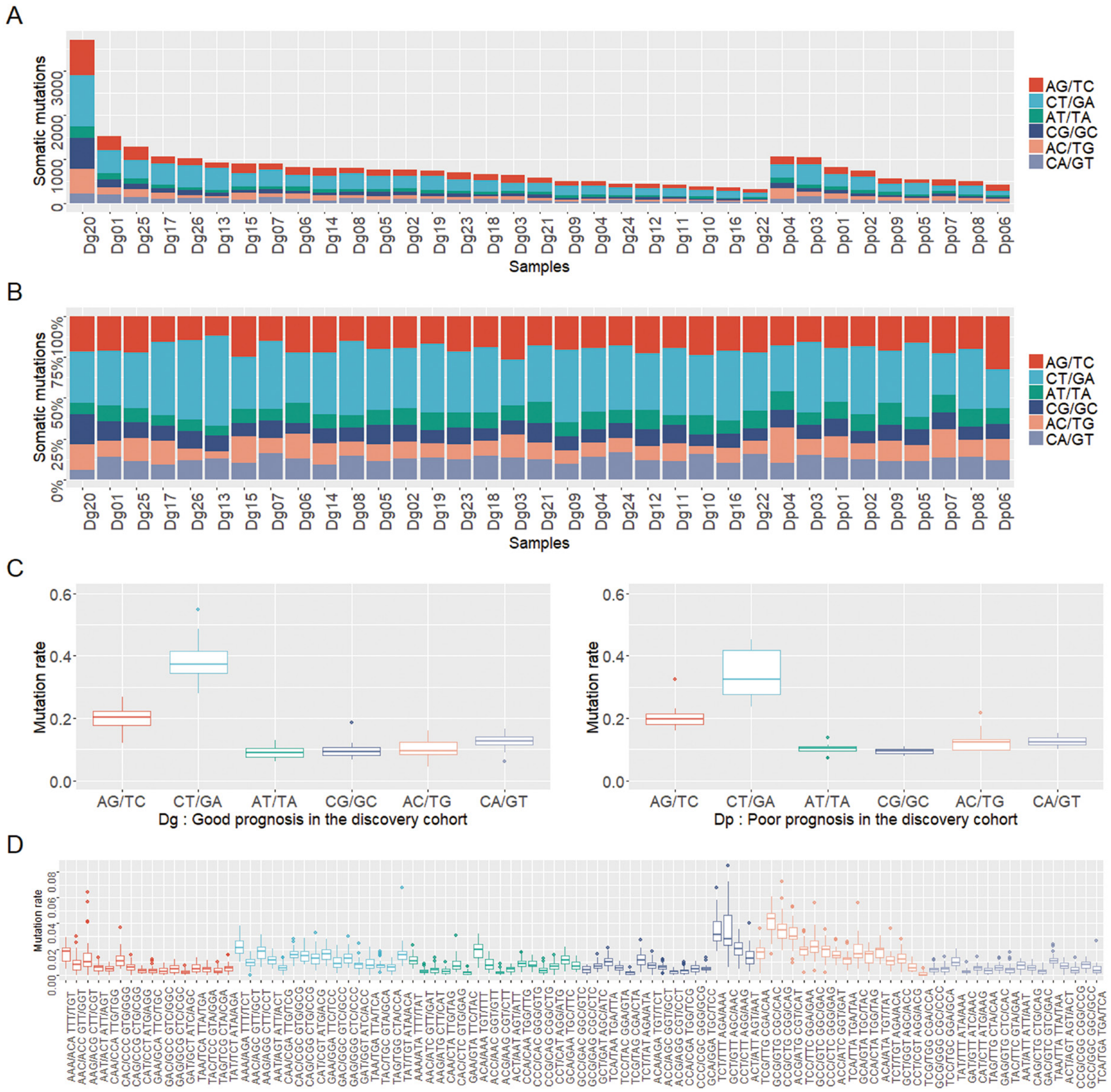

Supplementary Figure 1: Analysis of mutation overview. **(A)** The number of mutations. **(B, C)** The proportion of mutated bases. In both prognostic groups, CT/GA transversions were the most frequent variants. **(D)** Mutations as triplets, XCG XTG/CGX CAX, were frequently observed.

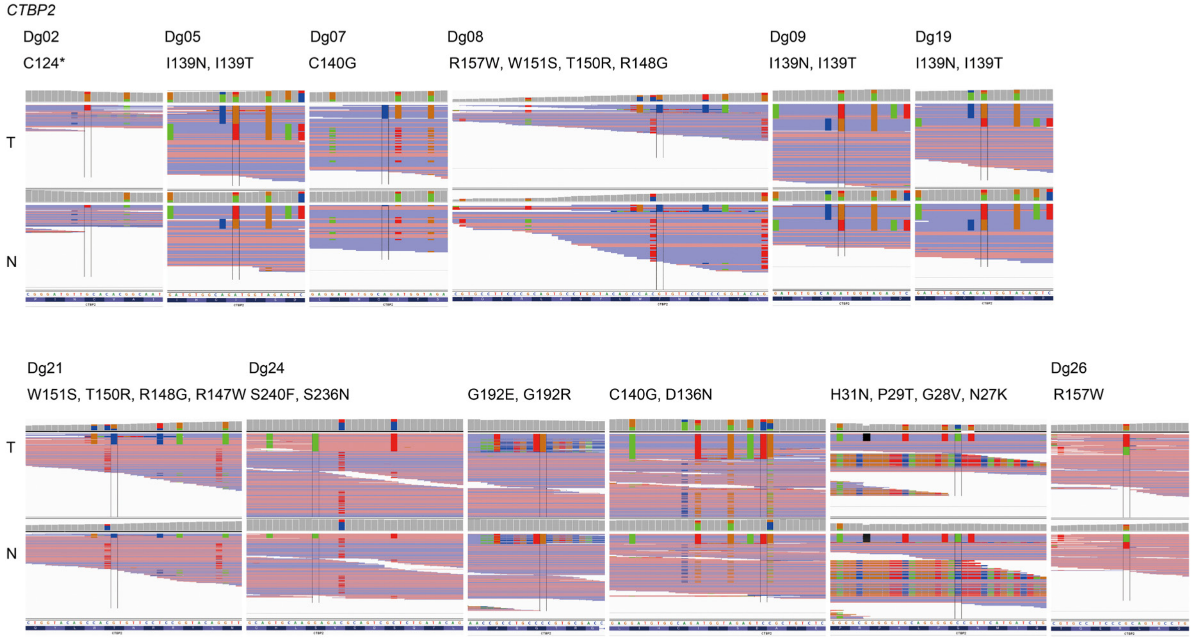

Supplementary Figure 2: Manual inspection with IGV. IGV view of *CTBP2* mutations. In both tumor and normal samples, many *CTBP2* mutations were detected from a single read, which is likely due to mapping errors.

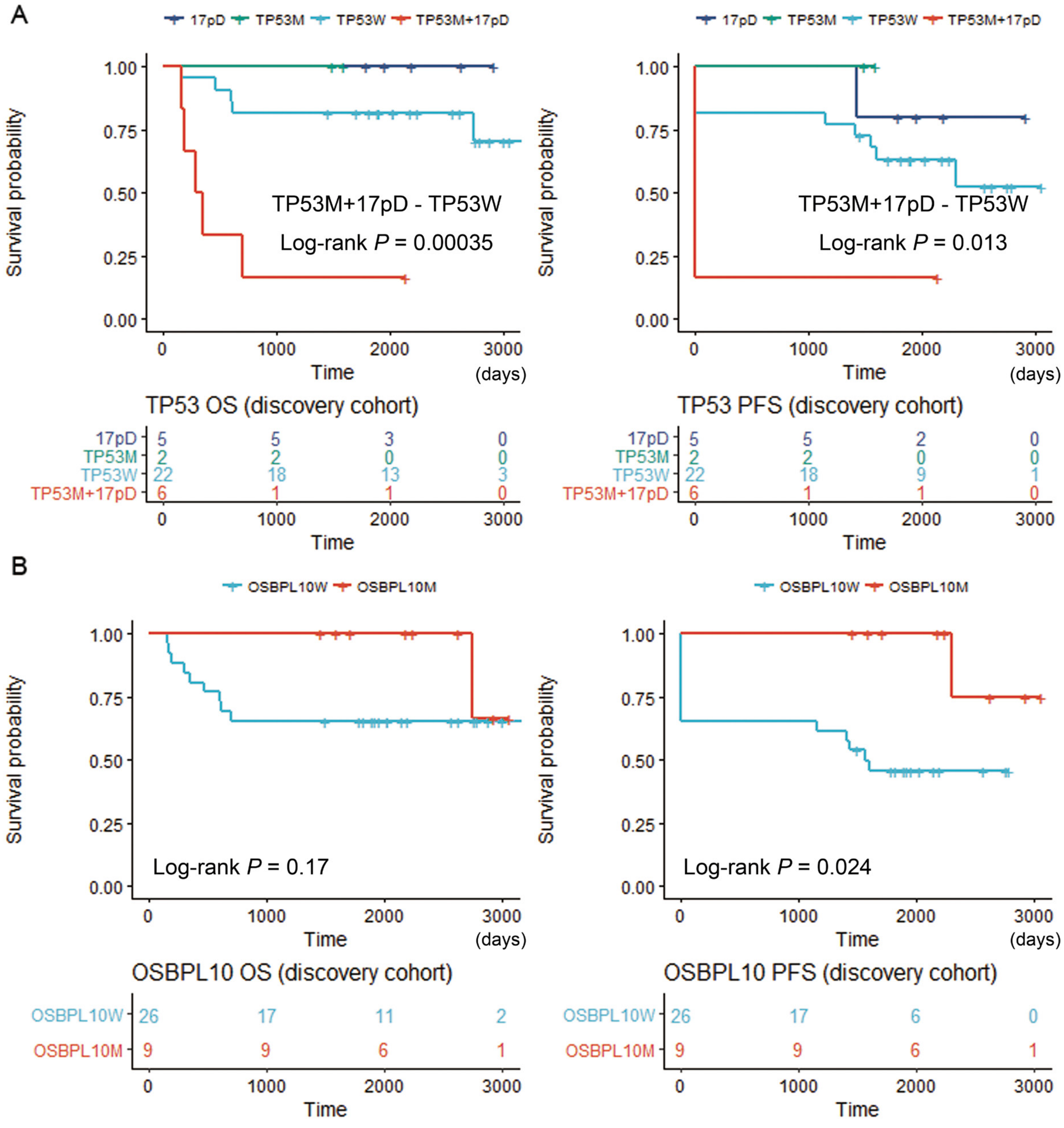

Supplementary Figure 3: Prognostic values of *TP53* and *OSBPL10* aberrations in the discovery cohort. **(A)** Survival stratified by *TP53* status in the discovery cohort. **(B)** Survival stratified by *OSBPL10* status in the discovery cohort. TP53W: *TP53* wild-type; TP53M: *TP53* mutation; 17pD: 17p deletion; OSBPL10W: *OSBPL10* wild-type; and OSBPL10M: *OSBPL10* mutation.

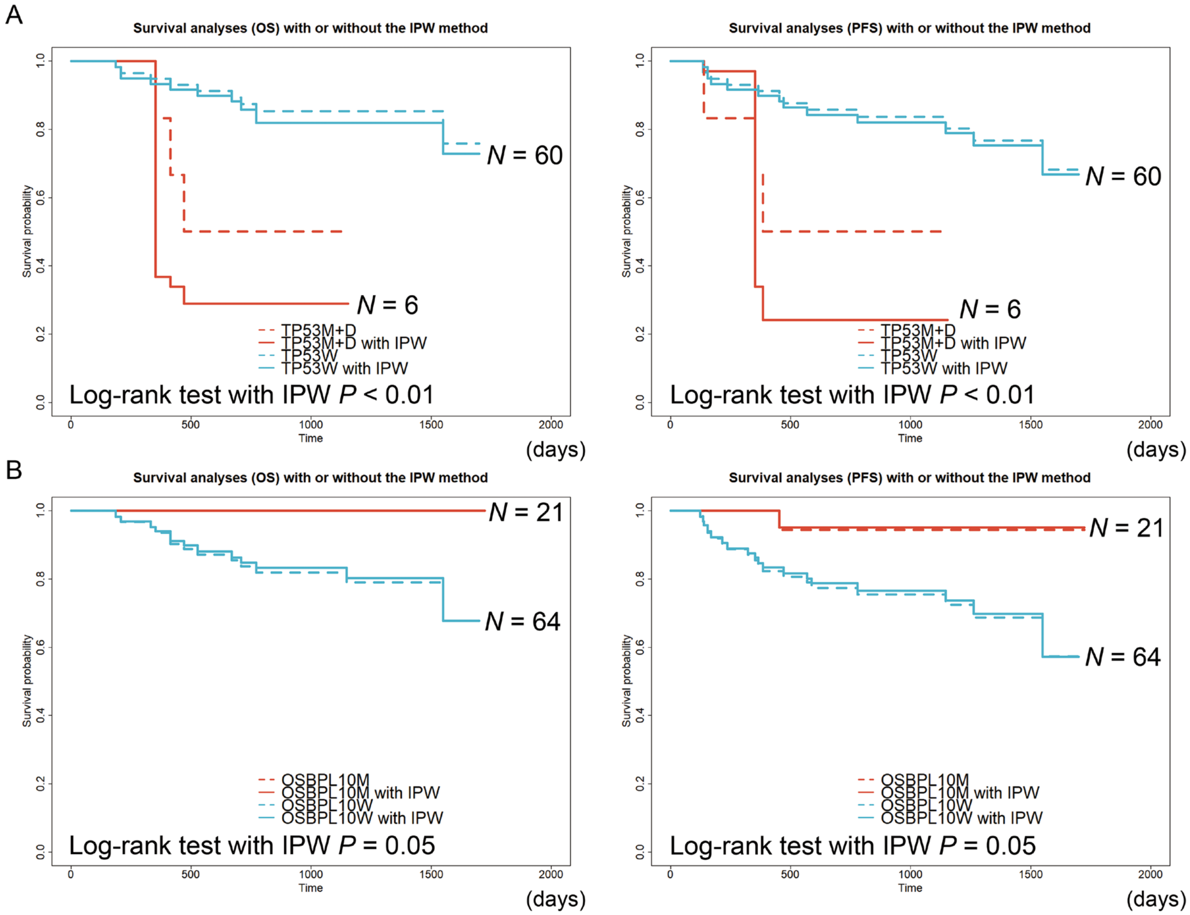

Supplementary Figure 4: Survival analyses with or without the IPW method. **(A)** Survival stratified by *TP53* status in the validation cohort. Variable IPI factors (age, clinical stage, LDH, ECOG-PS and extranodal lesion) and *OSBPL10* mutations were incorporated into the propensity score model. **(B)** Survival stratified by *OSBPL10* status in the validation cohort. Variable IPI factors (age, clinical stage, LDH, ECOG-PS and extranodal lesion) and *TP53* mutations with deletion were incorporated into the propensity score model. TP53W: *TP53* wild-type; TP53M + D: *TP53* mutation with deletion; OSBPL10W: *OSBPL10* wild-type, and OSBPL10M: *OSBPL10* mutation.

Supplementary Table 1: Clinical features of the cases

See Supplementary File 1

Supplementary Table 2: Summary of discovery cohort (Dp/Dg)

Case no.	Estimated tumor content	Tumor read count	Normal read count	Tumor mean coverage	Normal mean coverage
Dp01	49.76%	373940632	136990566	196.08	113.53
Dp02	85.31%	410472888	129055310	277.00	117.68
Dp03	75.26%	397613058	139262220	260.47	122.23
Dp04	40.01%	441213896	201387688	256.57	151.33
Dp05	89.16%	421244714	230629374	235.66	159.51
Dp06	53.90%	365128840	176025412	345.58	176.91
Dp07	55.59%	436434846	190379370	346.50	175.38
Dp08	54.93%	413668164	198094866	354.51	182.00
Dp09	83.09%	408791042	182424376	164.98	164.29
Dg01	69.01%	373607706	318469368	246.97	228.57
Dg02	42.79%	345028350	317723674	234.53	216.58
Dg03	30.98%	462074896	188928072	265.39	145.64
Dg04	46.91%	418957674	166095014	199.95	117.90
Dg05	69.17%	405672354	188951040	260.12	140.94
Dg06	46.74%	308925900	320051672	229.14	227.86
Dg07	57.43%	372825502	214892086	240.75	147.32
Dg08	81.94%	461159888	184365050	387.61	176.01
Dg09	31.24%	395665268	190121156	353.34	185.37
Dg10	46.69%	432748322	191748358	380.95	181.05
Dg11	31.65%	367943704	196731474	340.87	193.08
Dg12	59.04%	451985760	205511240	347.51	187.04
Dg13	63.36%	426718718	190970484	390.59	193.71
Dg14	49.21%	414677588	197144022	362.85	162.94
Dg15	75.68%	364776504	186065374	330.48	186.46
Dg16	50.79%	391319262	196419924	346.20	192.36
Dg17	44.64%	385161446	199274962	357.62	200.13
Dg18	37.35%	394245978	184924552	362.70	185.44
Dg19	60.05%	374064268	190517942	345.63	190.43
Dg20	64.73%	410967124	208163130	356.80	197.30
Dg21	67.39%	447603874	192539016	303.10	164.42
Dg22	36.74%	456688072	187724474	330.95	161.40
Dg23	35.98%	466685312	180109868	335.56	160.10
Dg24	72.68%	418532916	178647518	337.80	168.71
Dg25	54.81%	351967470	208790576	304.20	180.85
Dg26	62.59%	284959294	161439200	231.51	141.14

Supplementary Table 3: Mutations in discovery cohort (Dp/Dg) (five cases and above)

See Supplementary File 1

Supplementary Table 4: Mutations in validation cohort (V)

Case no.	CHR	POS	REF	ALT	refGene_Gene	refGene_No	refGene_AACH	SIFT	Deleterious: (≤ 0.05), Tolerated: (> 0.05)	Polyphen2_HVAR	Probably damaging : (≥ 0.957), Possibly damaging : (≥ 0.453), Benign : (< 0.453)
V14	chr17	7577029	G	C	TP53	NM_000546.5	S303R	0.06	Tolerated	0.817	Possibly damaging
V83	chr17	7577081	T	C	TP53	NM_000546.5	E286G	0	Deleterious	0.995	Probably damaging
V84	chr17	7577085	C	T	TP53	NM_000546.5	E285K	0	Deleterious	0.998	Probably damaging
V72	chr17	7577100	T	C	TP53	NM_000546.5	R280G	0	Deleterious	1	Probably damaging
V77	chr17	7577105	G	C	TP53	NM_000546.5	P278R	0	Deleterious	1	Probably damaging
V17	chr17	7577120	C	T	TP53	NM_000546.5	R273H	0.01	Deleterious	0.989	Probably damaging
V31	chr17	7577121	G	A	TP53	NM_000546.5	R273C	0	Deleterious	0.999	Probably damaging
V35	chr17	7577534	C	G	TP53	NM_000546.5	R249S	0.01	Deleterious	0.992	Probably damaging
V67	chr17	7577539	G	A	TP53	NM_000546.5	R248W	0	Deleterious	1	Probably damaging
V84	chr17	7577547	C	A	TP53	NM_000546.5	G245V	0	Deleterious	1	Probably damaging
V06	chr17	7577550	C	T	TP53	NM_000546.5	G244D	0	Deleterious	1	Probably damaging
V80	chr17	7577566	T	C	TP53	NM_000546.5	N239D	0	Deleterious	0.999	Probably damaging
V51	chr17	7577568	C	T	TP53	NM_000546.5	C238Y	0	Deleterious	1	Probably damaging
V68	chr17	7577569	A	G	TP53	NM_000546.5	C238R	0	Deleterious	1	Probably damaging
V68	chr17	7577572	T	G	TP53	NM_000546.5	M237L	0	Deleterious	0.999	Probably damaging
V68	chr17	7577583	T	A	TP53	NM_000546.5	H233L	1	Tolerated	0.055	Benign
V43	chr17	7578196	A	T	TP53	NM_000546.5	V218E	0	Deleterious	1	Probably damaging
V25	chr17	7578212	G	A	TP53	NM_000546.5	R213*	1	Tolerated	.	.
V76	chr17	7578526	C	A	TP53	NM_000546.5	C135F	0.01	Deleterious	1	Probably damaging
V78	chr17	7578550	G	T	TP53	NM_000546.5	S127Y	0	Deleterious	1	Probably damaging
V42	chr17	7579705	C	T	TP53	NM_000546.5	V31I	0.59	Tolerated	0.004	Benign
V14	chr17	7579882	C	G	TP53	NM_001126112.2	E11Q	0.03	Deleterious	0.972	Probably damaging
V50	chr3	32022391	C	G	OSBPL10	NM_001174060.1	R94T	.	.	0.993	Probably damaging
V56	chr3	32022391	C	G	OSBPL10	NM_001174060.1	R94T	.	.	0.993	Probably damaging
V52	chr3	32022402	GC	G	OSBPL10	NM_001174060.1	G90	.	.	.	.
V52	chr3	32022406	TGGAGGAGGTTGGTGTATTTGCTGAGCACGCCCTC	T	OSBPL10	NM_001174060.1	EGVLSKYTNLLQ78	.	.	.	.
V33	chr3	32022431	G	A	OSBPL10	NM_001174060.1	L81F	0	Deleterious	1	Probably damaging
V63	chr3	32022449	G	A	OSBPL10	NM_001174060.1	P75S	0.36	Tolerated	0.172	Benign
V46	chr3	32022480	G	T	OSBPL10	NM_001174060.1	S64R	0.06	Tolerated	0.029	Benign
V25	chr3	32022505	C	A	OSBPL10	NM_001174060.1	S56I	0.04	Deleterious	0.001	Benign
V39	chr3	32022541	G	A	OSBPL10	NM_001174060.1	S44L	0.39	Tolerated	0.005	Benign
V25	chr3	32022553	AC	A	OSBPL10	NM_001174060.1	V40	.	.	.	.
V61	chr3	32022557	C	T	OSBPL10	NM_001174060.1	G39R	0	Deleterious	0.131	Benign
V66	chr3	32022581	G	A	OSBPL10	NM_001174060.1	P31S	0.13	Tolerated	0.118	Benign
V66	chr3	32022601	G	C	OSBPL10	NM_001174060.1	A24G	0.36	Tolerated	0.079	Benign
V26	chr3	32022601	G	C	OSBPL10	NM_001174060.1	A24G	0.36	Tolerated	0.079	Benign
V81	chr3	32022602	C	T	OSBPL10	NM_001174060.1	A24T	0.14	Tolerated	0.059	Benign
V57	chr3	32022606	G	T	OSBPL10	NM_001174060.1	S22R	0.04	Deleterious	0.029	Benign
V14	chr3	32022613	C	A	OSBPL10	NM_001174060.1	S20I	0.16	Tolerated	0.795	Possibly damaging
V39	chr3	32022623	T	G	OSBPL10	NM_001174060.1	S17R	0.04	Deleterious	0.137	Benign
V62	chr3	32022625	C	G	OSBPL10	NM_001174060.1	S16T	0.3	Tolerated	0.332	Benign
V33	chr3	32022625	C	T	OSBPL10	NM_001174060.1	S16N	0.09	Tolerated	0.61	Possibly damaging
V48	chr3	32022625	C	T	OSBPL10	NM_001174060.1	S16N	0.09	Tolerated	0.61	Possibly damaging
V65	chr3	32022630	G	C	OSBPL10	NM_001174060.1	S14R	0.08	Tolerated	0.612	Possibly damaging
V02	chr3	32022631	C	G	OSBPL10	NM_001174060.1	S14T	0.23	Tolerated	0.31	Benign
V66	chr3	32022636	CCCG	C	OSBPL10	NM_001174060.1	GG11G	.	.	.	.
V46	chr3	32022642	GCCGTC	G	OSBPL10	NM_001174060.1	DG9	.	.	.	.
V40	chr3	32022646	T	C	OSBPL10	NM_001174060.1	D9G	0.2	Tolerated	0.001	Benign
V31	chr3	32022662	C	T	OSBPL10	NM_001174060.1	A4T	0.07	Tolerated	0.431	Benign
V26	chr3	32022668	C	T	OSBPL10	NM_001174060.1	E2K	0	Deleterious	0.227	Benign
V28	chr3	32022668	C	G	OSBPL10	NM_001174060.1	E2Q	0	Deleterious	0.362	Benign

Supplementary Table 5: Analysis of *TP53* gene copy number variation

See Supplementary File 1

Supplementary Table 6: *OSBPL10* was identified as a SHM target

Gene	Total SNVs	Motif mutation	Transition mutation	C:G mutation	Mutation case (total 35 cases)	Gene length	SHM indicator
PIM1*	588	426	406	583	19	1206	< 0.001
IGLL5	482	359	254	429	22	639	< 0.001
PABPC3	386	69	276	151	14	1892	< 0.001
KCNJ18	214	0	102	214	8	1298	< 0.001
CTBP2	209	31	93	111	12	2946	0.0025
ZNF717	161	14	89	135	19	2738	0.0018
MUC3A	161	25	75	76	17	4444	0.0068
ATAD3B	120	0	120	120	8	1928	< 0.001
MUC6	110	20	23	98	16	7284	< 0.001
LDHAL6B	108	27	84	30	3	1142	0.0073
MTCH2	107	3	81	32	12	929	< 0.001
CD79B	90	4	63	7	8	684	< 0.001
PABPC1	88	6	39	24	13	1894	< 0.001
SHANK1	87	29	86	59	7	6460	0.1289
MUC16	85	20	55	57	22	43437	0.7279
HLA-DRB5	83	36	40	36	9	792	0.0627
CDC27	76	30	66	0	7	2471	< 0.001
HLA-DRB1	74	37	32	35	8	3910	0.0408
MYD88	72	4	68	8	8	946	< 0.001
BTG1*	71	53	54	67	8	511	0.0024
DUSP2*	71	42	57	62	8	938	0.0273
WDR89	69	14	50	35	5	1160	0.2697
ANKLE1	68	0	0	8	9	1998	< 0.001
AK2	63	52	32	61	5	711	0.0013
HLA-A	63	17	37	52	9	8691	0.5857
SHANK3	61	21	61	17	4	5129	0.0176
GXYLT1	61	10	44	33	6	1312	0.2553
AQP7	58	1	32	57	6	1019	0.0060
SGK1*	52	23	30	30	4	1564	0.3608
SOCS1*	51	30	29	47	5	632	0.0716
HLA-DQB2	44	12	22	28	3	6288	0.6454
CNN2	43	31	39	24	5	983	0.0232
FMN2	43	10	24	24	9	5159	0.5862
HLA-B	41	22	13	30	6	6474	0.0885
MPEG1	40	28	31	40	3	2147	0.0307
HNRNPL	39	0	36	0	3	1754	< 0.001
FAM8A1	34	3	22	29	7	1234	0.2246
OSBPL10	30	28	22	27	11	2280	0.0175
KLRC2	29	25	4	25	7	687	0.0039
IGSF3	29	0	16	24	10	3631	0.0759
ARMC4	28	8	0	20	5	3113	0.0109
FAM205A	28	18	28	9	6	4001	0.0387
KMT5A	28	8	24	20	3	2792	0.6560
MESP2	26	1	26	16	5	1189	0.1239
BTG2*	26	14	16	23	7	472	0.3218
DSPP	24	6	24	6	2	3899	0.1411
BCL2*	24	14	18	23	6	715	0.1977
PRSS1	23	7	10	17	5	736	0.6881
ADAM21	22	0	22	10	4	2165	0.0649
DTX1*	22	16	16	20	6	1851	0.1379
TAS2R43	22	6	19	4	7	926	0.1430
HIST1H1E	22	13	18	22	12	656	0.1831

Supplementary Table 7: Confirmation by direct sequencing

See Supplementary File 1

Supplementary Table 8: Primers for real-time genomic PCR of *TP53* and *GAPDH*

Gene	Forward primer	Reverse primer	Product size (bp)
TP53	CACAGCACATGACGGAGGTTG	GTCATCCAAATACTCCACACGCA	209
GAPDH	CTGACCTGCCGTCTAGAAAAACCT	CAGGAAATGAGCTTGACAAAGTGG	206
