# Supplementary material for: TP53 and OSBPL10 alterations in diffuse large B-cell lymphoma: prognostic markers identified via exome analysis of cases with extreme prognosis
Source: Oncotarget. 2018 Apr 13;9(28):19555–68. doi: 10.18632/oncotarget.24656 (PMC5929408; doi:10.18632/oncotarget.24656)
Supplement: Supplementary file 2 [file oncotarget-09-19555-s002.docx]

Supplementary Table 1: Clinical features of the cases

| Case no. | Biopsy site | Treatment | IPI |
| --- | --- | --- | --- |
| Dp01 | colon | R-CHOP#8 > CHASER#2 > R-ICE#1 > R-DHAP#2 > Radiation > R#6-MA#1 > AlloSCT | Low intermediate risk |
| Dp02 | nasopharynx | R#8-CHOP#6 | High risk |
| Dp03 | right breast | R#8-CHOP#8 > IT#1 > R#8-ICE#3 > IT#1 > DHAP#3 > R-inotuzumab ozogamicin#1 | High risk |
| Dp04 | thyroid | R#8-CHOP#6 > VP16 > Radiation | High intermediate risk |
| Dp05 | supraclavicular lymph node | R-CHOP#6 > ICE#1 > DHAP#1 | Low intermediate risk |
| Dp06 | chest wall | R#7-CHOP#4 > R-ICE#1 | High intermediate risk |
| Dp07 | bone marrow | R#8-CHOP#5 > ICE#1 > DHAP#2 > Radiation | High risk |
| Dp08 | right inguinal lymph node | R#8-CHOP#6 > IT#5 > ICE#3 | Low risk |
| Dp09 | retroperitoneum (iliopsoas muscle) | R-CHOP#1 > IT#1 > R#4-DHAP#3 > R-ICE#2 > Radiation > R-ICE#2 > Radiation >R-ICE#1 | High risk |
| Dg01 | cervical lymph node | R#8-CHOP#6 (#4-#6 80%dose) + Radiation | Low risk |
| Dg02 | cervical lymph node | R#5-CHOP#5 | Low intermediate risk |
| Dg03 | tonsil | R#8-CHOP#3 + Radiation | Low risk |
| Dg04 | tonsil | R#8-CHOP#3 + Radiation | Low risk |
| Dg05 | right conjunctiva | R-CHOP#6 > IT#4 > R#4DHAP#3 | Low risk |
| Dg06 | cervical lymph node | R#8-CHOP#6 | Low intermediate risk |
| Dg07 | axillary lymph node | R-CHOP#6 | Low risk |
| Dg08 | left breast | R#4-CHOP#6 | High risk |
| Dg09 | right cervical lymph node | R#8-CHOP#6 | Low risk |
| Dg10 | root of tongue | R#8-CHOP#6 | High intermediate risk |
| Dg11 | cervical lymph node | R#8-CHOP#6 | Low risk |
| Dg12 | pharynx | R#8-CHOP#6 | Low risk |
| Dg13 | right breast | R#8-CHOP#6 | Low intermediate risk |
| Dg14 | thyroid | R-CHOP#6 > R-GDP#3 | Low intermediate risk |
| Dg15 | oral cavity | R#8-CHOP#6 | Low risk |
| Dg16 | cervical lymph node | R#8-CHOP#6 | Low risk |
| Dg17 | right breast | R-CHOP#3 + Radiation | Low risk |
| Dg18 | testis | R#5-CHOP#2 + Radiation | Low risk |
| Dg19 | tonsil | R-CHOP#6 | Low risk |
| Dg20 | cervical lymph node | R#8-CHOP#6 | Low intermediate risk |
| Dg21 | right nasal cavity | R#8-CHOP#6 | Low risk |
| Dg22 | small intestine | R#8-CHOP#6 | High intermediate risk |
| Dg23 | inguinal lymph node | R-CHO#6 > R-ICE#3 > High-MEC + AutoPBSCT > AZA#32 | Low intermediate risk |
| Dg24 | axillary lymph node | R-CHOP#6 > R#4-ICE#3 | High intermediate risk |
| Dg25 | tonsil | R-CHOP#8 > CHASER#3 (80%dose) | High intermediate risk |
| Dg26 | left nose | R#8-CH#7 + Radiation > R-ICE#1 > Radiation | Low risk |
| V01 | tonsil | R#8-CHOP#6 | Low risk |
| V02 | breast | R-CHOP#3 > RT | Low risk |
| V03 | orbit | R#8-CHOP(80%)#6 | High risk |
| V04 | tonsil | R-CHOP#8 | Low risk |
| V05 | neck | R#5-CHOP#3 | High risk |
| V06 | supraclavicular lymph node | R-CHOP#8 > Inotuzumab ozogamicin + R | Low risk |
| V07 | small intestine | R#8-CHOP#6 | Low intermediate risk |
| V08 | cervical lymph | Obinutuzumab#8-CHOP#6 | Low intermediate risk |
| V09 | paranasal cavity | R#8-CHOP#6 > RT | High intermediate risk |
| V10 | skin | R#8-CHOP#6 | Low risk |
| V11 | neck | R#8-CHOP#6 > R#5-GDP#3 | Low intermediate risk |
| V12 | gingiva | R#8-CHOP#6 | Low risk |
| V13 | axillary node | R#2-CHOP#2 | Low intermediate risk |
| V14 | pharynx | R-CHOP#6 | Low risk |
| V15 | neck | R-CHOP#6 | Low intermediate risk |
| V16 | cervical lymph | R#4-ICE#3 | Low intermediate risk |
| V17 | cervical lymph | R#8-CHOP#6 | Low risk |
| V18 | breast | IT#1 > R#8-CHOP#3 > RT | Low risk |
| V19 | cervical lymph | R#8-CHOP#6 > R-GDP#3 | High risk |
| V20 | breast | IT#1 > R#8-CHOP#6 | Low risk |
| V21 | neck | R#8-CHOP#6 > R#3-GDP#3 > ICE#1 > R#8-DHAP#4 | High intermediate risk |
| V22 | face skin | R-VP16#5 | Low intermediate risk |
| V23 | pharynx | R#8-CHOP#6 | Low risk |
| V24 | omentum and mesentery | R#8-CHOP#6 | Low intermediate risk |
| V25 | cervical lymph | R-CHOP#8 | Low intermediate risk |
| V26 | cervical lymph | IT#1 > R#8-CHOP#6 > IT#2 > R-DHAP(80%)#3 > R-ICE(80%)#3 > RTx30Gy | Low intermediate risk |
| V27 | neck | R#8-CHOP#6 > RT50Gy | Low intermediate risk |
| V28 | neck | R#8-CHOP#6 | High risk |
| V29 | tonsil | R#8-CHOP#6 > RT > R#3-GDP#2 > R-ICE#1 | Low risk |
| V30 | conjunctiva | R-CHOP#5 | Low risk |
| V31 | tonsil | R#8-CHOP#6 | Low risk |
| V32 | tonsil | R#8-CHOP#6 > RT30Gy > R-GDP#6 > ICE(80%)#3 > DHAP | Low risk |
| V33 | tonsil | R#8-CHOP#5 | Low risk |
| V34 | tonsil | R-CHOP#8 > PSL | High intermediate risk |
| V35 | clavicle | R#8-CHOP#6 | High intermediate risk |
| V36 | tonsil | R#8-CHOP#6 | High intermediate risk |
| V37 | skin | IT > R#8-CHOP#6 > VP16 > PSL | High intermediate risk |
| V38 | chest wall | R#8-CHOP#6 + IT#1 > RT50Gy > R-ICE#6 > R-GDP#1 | High risk |
| V39 | pharynx | R#8-CHOP#6 | High intermediate risk |
| V40 | maxilla | IT > R#8-CHOP#5 | High risk |
| V41 | tonsil | R-CHOP#6 | Low risk |
| V42 | tonsil | R-CHOP#6 | High intermediate risk |
| V43 | back | IT#4 > R#8-CHOP#6 | High risk |
| V44 | neck | R#8-CHOP#6 | Low intermediate risk |
| V45 | inguinal lymph node | R#8-CHOP#6 | Low intermediate risk |
| V46 | mesenteric lymph node | R#8-CHOP#6 | High intermediate risk |
| V47 | tonsil | R#8-CHOP#6 | Low risk |
| V48 | nasal cavity | R#8-CHOP#6 | Low risk |
| V49 | nasal cavity | R-CHOP±Ibrutinib#6 > IT#4 | Low intermediate risk |
| V50 | tonsil | R-CHOP | High intermediate risk |
| V51 | tonsil | R-CHOP#3 > R-GDP#3 > RT30Gy+45Gy > R-ICE(70%)#1 | High intermediate risk |
| V52 | left arm | R#8-GDP#3 | Low intermediate risk |
| V53 | larynx | R-VP16 | High intermediate risk |
| V54 | inguinal lymph node | R#8-CHOP#6 | High intermediate risk |
| V55 | cervical lymph | R-CHOP#6 | Low risk |
| V56 | breast | R#8-CHOP#6 > IT#4 | Low risk |
| V57 | mesopharynx | R#3-CHOP#3 | Low risk |
| V58 | cervical lymph | R#8-CHOP#6 | Low intermediate risk |
| V59 | neck | R#8-CHOP#6 | High intermediate risk |
| V60 | femur | R-CHOP(80%)#3 > R-GDP#3 > R#8 | High risk |
| V61 | cervical lymph | R#8-CHOP#6 | Low risk |
| V62 | neck | R#8-CHOP#6 | High risk |
| V63 | tonsil | R#8-CHOP#6 | High intermediate risk |
| V64 | iliac bone | R#8-CHOP#6 > RT50Gy | High intermediate risk |
| V65 | neck | R#8-CHOP(80%)#8 | High intermediate risk |
| V66 | breast | R#8-CHOP#6 | Low risk |
| V67 | retroperitoneum | R-CHOP(70%)#1 | Low intermediate risk |
| V68 | neck | R-CHOP#1 | Low intermediate risk |
| V69 | spleen | R-CHOP(80%)#6 | High intermediate risk |
| V70 | orbit | R#8-CHOP#3 > RT | Low risk |
| V71 | mesopharynx | R#8-CHOP#6 | Low risk |
| V72 | cervical lymph | R-CEOP#1 > R#3-ICE#2 > R#5 | High intermediate risk |
| V73 | abdominal cavity | R-CHOP#6 | Low risk |
| V74 | stomach and lymph node | R-CHOP#6 | Low risk |
| V75 | left orchiectomy | IT#1 > R#8-CHOP#6 > IT#2 + High-dose methotrexate#2 > IT#2 + R-GDP#1 > High MEC > AutoSCT | Low intermediate risk |
| V76 | spleen | R#8-CHOP#8 > RT > GDP#2 > ICE(80%)#1 > R-DHAP(80%)#1 | Low intermediate risk |
| V77 | neck | R-ICE#2 > R-VP16 > R#5 > RT | High intermediate risk |
| V78 | retroperitoneum | R#6-ICE(70%)#3 > GDP(80%)#3 > VP16#21 | High intermediate risk |
| V79 | duodenum | R#8-CHOP#6 | Low intermediate risk |
| V80 | pelvic | R#8-CHOP#6 | High risk |
| V81 | tongue | R#8-CHOP#6 | Low risk |
| V82 | tonsil | R#8-CHOP#6 | Low risk |
| V83 | neck | R#8-CHOP#6 | High intermediate risk |
| V84 | retroperitoneal lymph node | R#6-CHP(80%)#6 | High intermediate risk |
| V85 | spleen | R#8-CHOP#6 > GDP#3 | High intermediate risk |

Supplementary Table 3: Mutations in discovery cohort (Dp/Dg) (five cases and above)

| Case_No | CHR | POS |  | REF | ALT | refGene_Gene | Cases | refGene_No | refGene_AACH |
| --- | --- | --- | --- | --- | --- | --- | --- | --- | --- |
| Dg01 | chr22 | 23230258 |  | G | C | IGLL5 | 21 | NM_001178126.1 | G9R |
| Dg01 | chr22 | 23230268 |  | C | T | IGLL5 | 21 | NM_001178126.1 | T12I |
| Dg01 | chr22 | 23230278 |  | G | T | IGLL5 | 21 | NM_001178126.1 | E15D |
| Dg01 | chr22 | 23230303 |  | TG | T | IGLL5 | 21 | NM_001178126.1 | W24 |
| Dg01 | chr22 | 23230318 |  | C | G | IGLL5 | 21 | NM_001178126.1 | L29V |
| Dg01 | chr22 | 23230321 |  | G | T | IGLL5 | 21 | NM_001178126.1 | G30C |
| Dg01 | chr22 | 23230357 |  | A | G | IGLL5 | 21 | NM_001178126.1 | M42V |
| Dg01 | chr22 | 23230361 |  | T | A | IGLL5 | 21 | NM_001178126.1 | V43D |
| Dg01 | chr22 | 23230366 |  | C | G | IGLL5 | 21 | NM_001178126.1 | P45A |
| Dg01 | chr22 | 23230383 |  | A | C | IGLL5 | 21 | NM_001256296.1 | Q15P |
| Dg01 | chr22 | 23230399 |  | G | A | IGLL5 | 21 | NM_001178126.1 | V56I |
| Dg01 | chr22 | 23230403 |  | G | T | IGLL5 | 21 | NM_001256296.1 | E22* |
| Dg01 | chr22 | 23230419 |  | C | G | IGLL5 | 21 | NM_001178126.1 | S62R |
| Dg01 | chr22 | 23230420 |  | C | A | IGLL5 | 21 | NM_001178126.1 | L63M |
| Dg01 | chr22 | 23230422 |  | G | A | IGLL5 | 21 | NM_001256296.1 | C28Y |
| Dg01 | chr22 | 23230428 |  | C | G | IGLL5 | 21 | NM_001178126.1 | S65R |
| Dg03 | chr22 | 23230363 |  | G | T | IGLL5 | 21 | NM_001178126.1 | A44S |
| Dg03 | chr22 | 23230398 |  | A | G | IGLL5 | 21 | NM_001256296.1 | Q20R |
| Dg05 | chr22 | 23230318 |  | C | G | IGLL5 | 21 | NM_001178126.1 | L29V |
| Dg05 | chr22 | 23230328 |  | C | A | IGLL5 | 21 | NM_001178126.1 | A32D |
| Dg05 | chr22 | 23230358 |  | T | A | IGLL5 | 21 | NM_001178126.1 | M42K |
| Dg05 | chr22 | 23230368 |  | G | A | IGLL5 | 21 | NM_001256296.1 | R10H |
| Dg05 | chr22 | 23230393 |  | G | A | IGLL5 | 21 | NM_001178126.1 | A54T |
| Dg07 | chr22 | 23230292 |  | C | T | IGLL5 | 21 | NM_001178126.1 | P20L |
| Dg07 | chr22 | 23230295 |  | G | C | IGLL5 | 21 | NM_001178126.1 | R21T |
| Dg07 | chr22 | 23230297 |  | C | T | IGLL5 | 21 | NM_001178126.1 | Q22* |
| Dg07 | chr22 | 23230328 |  | C | T | IGLL5 | 21 | NM_001178126.1 | A32V |
| Dg07 | chr22 | 23230348 |  | C | G | IGLL5 | 21 | NM_001178126.1 | L39V |
| Dg07 | chr22 | 23230363 |  | G | A | IGLL5 | 21 | NM_001178126.1 | A44T |
| Dg07 | chr22 | 23230366 |  | C | T | IGLL5 | 21 | NM_001178126.1 | P45S |
| Dg07 | chr22 | 23230369 |  | C | T | IGLL5 | 21 | NM_001178126.1 | Q46* |
| Dg07 | chr22 | 23230393 |  | G | A | IGLL5 | 21 | NM_001178126.1 | A54T |
| Dg07 | chr22 | 23230398 |  | A | T | IGLL5 | 21 | NM_001256296.1 | Q20L |
| Dg07 | chr22 | 23230436 |  | G | T | IGLL5 | 21 | NM_001178126.1 | G68V |
| Dg07 | chr22 | 23235879 |  | GGCTCCTGCT | G | IGLL5 | 21 | NM_001178126.1 | RLLL69S |
| Dg07 | chr22 | 23235887 |  | C | A | IGLL5 | 21 | NM_001178126.1 | L72I |
| Dg08 | chr22 | 23230255 |  | G | C | IGLL5 | 21 | NM_001178126.1 | V8L |
| Dg08 | chr22 | 23230364 |  | C | A | IGLL5 | 21 | NM_001178126.1 | A44E |
| Dg08 | chr22 | 23235907 |  | A | C | IGLL5 | 21 | NM_001178126.1 | R78S |
| Dg09 | chr22 | 23230410 |  | C | T | IGLL5 | 21 | NM_001256296.1 | A24V |
| Dg09 | chr22 | 23230439 |  | G | A | IGLL5 | 21 | NM_001178126.1 | R69K |
| Dg10 | chr22 | 23230268 |  | C | T | IGLL5 | 21 | NM_001178126.1 | T12I |
| Dg11 | chr22 | 23230415 |  | C | G | IGLL5 | 21 | NM_001178126.1 | S61C |
| Dg11 | chr22 | 23235966 |  | T | C | IGLL5 | 21 | NM_001178126.1 | V98A |
| Dg12 | chr22 | 23230279 |  | C | G | IGLL5 | 21 | NM_001178126.1 | L16V |
| Dg14 | chr22 | 23230311 |  | GC | G | IGLL5 | 21 | NM_001178126.1 | L27 |
| Dg14 | chr22 | 23230315 |  | C | G | IGLL5 | 21 | NM_001178126.1 | L28V |
| Dg14 | chr22 | 23230348 |  | C | G | IGLL5 | 21 | NM_001178126.1 | L39V |
| Dg14 | chr22 | 23230373 |  | G | T | IGLL5 | 21 | NM_001178126.1 | S47I |
| Dg14 | chr22 | 23230376 |  | G | A | IGLL5 | 21 | NM_001178126.1 | G48E |
| Dg14 | chr22 | 23230377 |  | G | A | IGLL5 | 21 | NM_001256296.1 | G13E |
| Dg14 | chr22 | 23230418 |  | G | A | IGLL5 | 21 | NM_001178126.1 | S62N |
| Dg17 | chr22 | 23230322 |  | G | C | IGLL5 | 21 | NM_001178126.1 | G30A |
| Dg17 | chr22 | 23230348 |  | C | G | IGLL5 | 21 | NM_001178126.1 | L39V |
| Dg17 | chr22 | 23230363 |  | G | T | IGLL5 | 21 | NM_001178126.1 | A44S |
| Dg17 | chr22 | 23230370 |  | A | G | IGLL5 | 21 | NM_001178126.1 | Q46R |
| Dg17 | chr22 | 23230388 |  | C | T | IGLL5 | 21 | NM_001178126.1 | P52L |
| Dg17 | chr22 | 23230408 |  | A | G | IGLL5 | 21 | NM_001178126.1 | S59G |
| Dg18 | chr22 | 23230348 |  | C | A | IGLL5 | 21 | NM_001256296.1 | C3* |
| Dg18 | chr22 | 23230359 |  | G | A | IGLL5 | 21 | NM_001256296.1 | W7* |
| Dg20 | chr22 | 23230234 |  | A | G | IGLL5 | 21 | NM_001178126.1 | M1V |
| Dg20 | chr22 | 23230259 |  | G | A | IGLL5 | 21 | NM_001178126.1 | G9D |
| Dg20 | chr22 | 23230315 |  | C | G | IGLL5 | 21 | NM_001178126.1 | L28V |
| Dg20 | chr22 | 23230315 |  | C | A | IGLL5 | 21 | NM_001178126.1 | L28M |
| Dg20 | chr22 | 23230327 |  | G | A | IGLL5 | 21 | NM_001178126.1 | A32T |
| Dg20 | chr22 | 23230337 |  | C | T | IGLL5 | 21 | NM_001178126.1 | A35V |
| Dg20 | chr22 | 23230347 |  | G | T | IGLL5 | 21 | NM_001256296.1 | C3F |
| Dg20 | chr22 | 23230361 |  | T | C | IGLL5 | 21 | NM_001178126.1 | V43A |
| Dg20 | chr22 | 23230383 |  | A | T | IGLL5 | 21 | NM_001256296.1 | Q15L |
| Dg20 | chr22 | 23230399 |  | G | A | IGLL5 | 21 | NM_001178126.1 | V56I |
| Dg20 | chr22 | 23230405 |  | A | G | IGLL5 | 21 | NM_001178126.1 | S58G |
| Dg20 | chr22 | 23230406 |  | G | A | IGLL5 | 21 | NM_001178126.1 | S58N |
| Dg20 | chr22 | 23230410 |  | C | G | IGLL5 | 21 | NM_001178126.1 | S59R |
| Dg20 | chr22 | 23230428 |  | C | G | IGLL5 | 21 | NM_001178126.1 | S65R |
| Dg20 | chr22 | 23230435 |  | G | A | IGLL5 | 21 | NM_001178126.1 | G68S |
| Dg20 | chr22 | 23230439 |  | G | A | IGLL5 | 21 | NM_001178126.1 | R69K |
| Dg21 | chr22 | 23230301 |  | G | A | IGLL5 | 21 | NM_001178126.1 | R23H |
| Dg21 | chr22 | 23230394 |  | C | T | IGLL5 | 21 | NM_001178126.1 | A54V |
| Dg23 | chr22 | 23230410 |  | C | G | IGLL5 | 21 | NM_001178126.1 | S59R |
| Dg23 | chr22 | 23230428 |  | C | T | IGLL5 | 21 | NM_001256296.1 | A30V |
| Dg25 | chr22 | 23230306 |  | C | A | IGLL5 | 21 | NM_001178126.1 | P25T |
| Dg25 | chr22 | 23230310 |  | T | A | IGLL5 | 21 | NM_001178126.1 | L26Q |
| Dg25 | chr22 | 23230353 |  | C | T | IGLL5 | 21 | NM_001256296.1 | A5V |
| Dg25 | chr22 | 23230357 |  | A | T | IGLL5 | 21 | NM_001178126.1 | M42L |
| Dg25 | chr22 | 23230366 |  | C | T | IGLL5 | 21 | NM_001178126.1 | P45S |
| Dg25 | chr22 | 23230439 |  | G | C | IGLL5 | 21 | NM_001178126.1 | R69T |
| Dg25 | chr22 | 23235962 |  | T | C | IGLL5 | 21 | NM_001178126.1 | Y97H |
| Dg25 | chr22 | 23235966 |  | T | C | IGLL5 | 21 | NM_001178126.1 | V98A |
| Dg25 | chr22 | 23235968 |  | T | A | IGLL5 | 21 | NM_001178126.1 | F99I |
| Dg26 | chr22 | 23230321 |  | G | A | IGLL5 | 21 | NM_001178126.1 | G30S |
| Dg26 | chr22 | 23230416 |  | C | CAGTTGG | IGLL5 | 21 | NM_001178126.1 | S62SWS |
| Dg26 | chr22 | 23230419 |  | C | G | IGLL5 | 21 | NM_001178126.1 | S62R |
| Dg26 | chr22 | 23230421 |  | T | A | IGLL5 | 21 | NM_001178126.1 | L63Q |
| Dg26 | chr22 | 23230424 |  | G | C | IGLL5 | 21 | NM_001178126.1 | R64P |
| Dg26 | chr22 | 23230428 |  | C | T | IGLL5 | 21 | NM_001256296.1 | A30V |
| Dg26 | chr22 | 23237748 |  | G | C | IGLL5 | 21 | NM_001178126.1 | K173N |
| Dg26 | chr22 | 23237805 |  | C | G | IGLL5 | 21 | NM_001178126.1 | S192R |
| Dp01 | chr22 | 23230234 |  | A | C | IGLL5 | 21 | NM_001178126.1 | M1L |
| Dp01 | chr22 | 23230312 |  | C | G | IGLL5 | 21 | NM_001178126.1 | L27V |
| Dp01 | chr22 | 23230313 |  | T | A | IGLL5 | 21 | NM_001178126.1 | L27Q |
| Dp01 | chr22 | 23230346 |  | T | A | IGLL5 | 21 | NM_001178126.1 | L38Q |
| Dp04 | chr22 | 23230308 |  | CCTGCTGCTGCTGG | C | IGLL5 | 21 | NM_001178126.1 | LLLLG26 |
| Dp04 | chr22 | 23230343 |  | G | A | IGLL5 | 21 | NM_001178126.1 | G37D |
| Dp04 | chr22 | 23230357 |  | A | T | IGLL5 | 21 | NM_001178126.1 | M42L |
| Dp04 | chr22 | 23230385 |  | A | C | IGLL5 | 21 | NM_001178126.1 | D51A |
| Dp04 | chr22 | 23230415 |  | C | A | IGLL5 | 21 | NM_001178126.1 | S61Y |
| Dp05 | chr22 | 23230347 |  | G | A | IGLL5 | 21 | NM_001256296.1 | C3Y |
| Dp05 | chr22 | 23230366 |  | C | T | IGLL5 | 21 | NM_001178126.1 | P45S |
| Dp08 | chr22 | 23230312 |  | C | G | IGLL5 | 21 | NM_001178126.1 | L27V |
| Dp08 | chr22 | 23230336 |  | G | A | IGLL5 | 21 | NM_001178126.1 | A35T |
| Dg02 | chr3 | 75790860 |  | C | G | ZNF717 | 18 | NM_001128223.1 | V29L |
| Dg02 | chr3 | 75790860 |  | C | T | ZNF717 | 18 | NM_001128223.1 | V29M |
| Dg03 | chr3 | 75786315 |  | C | T | ZNF717 | 18 | NM_001128223.1 | R820K |
| Dg04 | chr3 | 75787276 |  | T | C | ZNF717 | 18 | NM_001128223.1 | T500A |
| Dg04 | chr3 | 75788226 |  | C | G | ZNF717 | 18 | NM_001128223.1 | C183S |
| Dg04 | chr3 | 75788226 |  | C | A | ZNF717 | 18 | NM_001128223.1 | C183F |
| Dg05 | chr3 | 75786315 |  | C | T | ZNF717 | 18 | NM_001128223.1 | R820K |
| Dg07 | chr3 | 75787405 |  | C | T | ZNF717 | 18 | NM_001128223.1 | G457R |
| Dg07 | chr3 | 75790824 |  | C | T | ZNF717 | 18 | NM_001128223.1 | D41N |
| Dg10 | chr3 | 75788152 |  | T | G | ZNF717 | 18 | NM_001128223.1 | T208P |
| Dg10 | chr3 | 75788152 |  | T | C | ZNF717 | 18 | NM_001128223.1 | T208A |
| Dg10 | chr3 | 75788260 |  | C | T | ZNF717 | 18 | NM_001128223.1 | G172R |
| Dg11 | chr3 | 75790837 |  | C | A | ZNF717 | 18 | NM_001128223.1 | W36C |
| Dg13 | chr3 | 75781272 |  | C | A | ZNF717 | 18 | NM_001290210.1 | G93V |
| Dg13 | chr3 | 75786315 |  | C | T | ZNF717 | 18 | NM_001128223.1 | R820K |
| Dg13 | chr3 | 75787273 |  | G | T | ZNF717 | 18 | NM_001128223.1 | H501N |
| Dg16 | chr3 | 75786315 |  | C | T | ZNF717 | 18 | NM_001128223.1 | R820K |
| Dg19 | chr3 | 75781272 |  | C | A | ZNF717 | 18 | NM_001290210.1 | G93V |
| Dg20 | chr3 | 75786315 |  | C | T | ZNF717 | 18 | NM_001128223.1 | R820K |
| Dg20 | chr3 | 75787343 |  | T | G | ZNF717 | 18 | NM_001128223.1 | K477N |
| Dg21 | chr3 | 75787179 |  | T | C | ZNF717 | 18 | NM_001128223.1 | E532G |
| Dg23 | chr3 | 75786315 |  | C | T | ZNF717 | 18 | NM_001128223.1 | R820K |
| Dg24 | chr3 | 75781257 |  | T | G | ZNF717 | 18 | NM_001290210.1 | Q98P |
| Dg24 | chr3 | 75786132 |  | T | C | ZNF717 | 18 | NM_001128223.1 | Q881R |
| Dg24 | chr3 | 75786501 |  | T | A | ZNF717 | 18 | NM_001128223.1 | Y758F |
| Dg24 | chr3 | 75788226 |  | C | A | ZNF717 | 18 | NM_001128223.1 | C183F |
| Dg25 | chr3 | 75786740 |  | C | G | ZNF717 | 18 | NM_001128223.1 | M678I |
| Dp01 | chr3 | 75786556 |  | A | C | ZNF717 | 18 | NM_001128223.1 | C740G |
| Dp01 | chr3 | 75788226 |  | C | G | ZNF717 | 18 | NM_001128223.1 | C183S |
| Dp01 | chr3 | 75788226 |  | C | A | ZNF717 | 18 | NM_001128223.1 | C183F |
| Dp03 | chr3 | 75788226 |  | C | G | ZNF717 | 18 | NM_001128223.1 | C183S |
| Dp07 | chr3 | 75788130 |  | C | T | ZNF717 | 18 | NM_001128223.1 | G215E |
| Dg02 | chr6 | 37138638 |  | G | A | PIM1 | 17 | NM_001243186.1 | V149I |
| Dg03 | chr6 | 37138427 |  | G | C | PIM1 | 17 | NM_001243186.1 | A117P |
| Dg04 | chr6 | 37138642 |  | C | G | PIM1 | 17 | NM_001243186.1 | S150C |
| Dg05 | chr6 | 37138260 |  | G | A | PIM1 | 17 | NM_001243186.1 | S61N |
| Dg05 | chr6 | 37138549 |  | G | A | PIM1 | 17 | NM_001243186.1 | G119D |
| Dg05 | chr6 | 37138577 |  | G | C | PIM1 | 17 | NM_001243186.1 | Q128H |
| Dg05 | chr6 | 37139199 |  | G | A | PIM1 | 17 | NM_001243186.1 | G271D |
| Dg05 | chr6 | 37139247 |  | C | T | PIM1 | 17 | NM_001243186.1 | T287I |
| Dg07 | chr6 | 37138272 |  | G | C | PIM1 | 17 | NM_001243186.1 | S65T |
| Dg07 | chr6 | 37138340 |  | C | G | PIM1 | 17 | NM_001243186.1 | L88V |
| Dg07 | chr6 | 37138354 |  | G | C | PIM1 | 17 | NM_002648.3 | M1I |
| Dg07 | chr6 | 37138549 |  | G | A | PIM1 | 17 | NM_001243186.1 | G119D |
| Dg07 | chr6 | 37138554 |  | G | A | PIM1 | 17 | NM_001243186.1 | E121K |
| Dg07 | chr6 | 37138560 |  | G | A | PIM1 | 17 | NM_001243186.1 | E123K |
| Dg07 | chr6 | 37138572 |  | T | C | PIM1 | 17 | NM_001243186.1 | S127P |
| Dg07 | chr6 | 37138902 |  | C | G | PIM1 | 17 | NM_001243186.1 | P172R |
| Dg07 | chr6 | 37139045 |  | C | T | PIM1 | 17 | NM_001243186.1 | L220F |
| Dg07 | chr6 | 37139209 |  | G | T | PIM1 | 17 | NM_001243186.1 | K274N |
| Dg08 | chr6 | 37138563 |  | C | T | PIM1 | 17 | NM_001243186.1 | P124S |
| Dg08 | chr6 | 37138908 |  | G | T | PIM1 | 17 | NM_001243186.1 | G174V |
| Dg08 | chr6 | 37139210 |  | C | G | PIM1 | 17 | NM_001243186.1 | L275V |
| Dg09 | chr6 | 37139210 |  | C | T | PIM1 | 17 | NM_001243186.1 | L275F |
| Dg11 | chr6 | 37138956 |  | G | A | PIM1 | 17 | NM_001243186.1 | G190D |
| Dg11 | chr6 | 37139063 |  | G | A | PIM1 | 17 | NM_001243186.1 | E226K |
| Dg11 | chr6 | 37139237 |  | C | T | PIM1 | 17 | NM_001243186.1 | L284F |
| Dg13 | chr6 | 37138243 |  | C | A | PIM1 | 17 | NM_001243186.1 | S55R |
| Dg13 | chr6 | 37138248 |  | G | A | PIM1 | 17 | NM_001243186.1 | S57N |
| Dg13 | chr6 | 37138332 |  | C | G | PIM1 | 17 | NM_001243186.1 | A85G |
| Dg13 | chr6 | 37138354 |  | G | A | PIM1 | 17 | NM_002648.3 | M1I |
| Dg13 | chr6 | 37138423 |  | G | C | PIM1 | 17 | NM_001243186.1 | K115N |
| Dg13 | chr6 | 37138554 |  | G | C | PIM1 | 17 | NM_001243186.1 | E121Q |
| Dg13 | chr6 | 37138563 |  | C | T | PIM1 | 17 | NM_001243186.1 | P124S |
| Dg13 | chr6 | 37138577 |  | G | C | PIM1 | 17 | NM_001243186.1 | Q128H |
| Dg13 | chr6 | 37138609 |  | G | A | PIM1 | 17 | NM_001243186.1 | G139D |
| Dg13 | chr6 | 37138901 |  | C | T | PIM1 | 17 | NM_001243186.1 | P172S |
| Dg13 | chr6 | 37138908 |  | G | A | PIM1 | 17 | NM_001243186.1 | G174D |
| Dg13 | chr6 | 37138962 |  | C | T | PIM1 | 17 | NM_001243186.1 | S192F |
| Dg13 | chr6 | 37139027 |  | CCCGAG | C | PIM1 | 17 | NM_001243186.1 | PE214 |
| Dg13 | chr6 | 37139039 |  | C | T | PIM1 | 17 | NM_001243186.1 | Q218* |
| Dg13 | chr6 | 37139063 |  | G | A | PIM1 | 17 | NM_001243186.1 | E226K |
| Dg13 | chr6 | 37139069 |  | G | T | PIM1 | 17 | NM_001243186.1 | G228* |
| Dg13 | chr6 | 37139138 |  | A | T | PIM1 | 17 | NM_001243186.1 | N251Y |
| Dg13 | chr6 | 37139203 |  | G | C | PIM1 | 17 | NM_001243186.1 | E272D |
| Dg17 | chr6 | 37138243 |  | C | G | PIM1 | 17 | NM_001243186.1 | S55R |
| Dg17 | chr6 | 37138249 |  | C | G | PIM1 | 17 | NM_001243186.1 | S57R |
| Dg17 | chr6 | 37138290 |  | G | A | PIM1 | 17 | NM_001243186.1 | G71D |
| Dg17 | chr6 | 37138309 |  | C | A | PIM1 | 17 | NM_001243186.1 | S77R |
| Dg17 | chr6 | 37138332 |  | C | T | PIM1 | 17 | NM_001243186.1 | A85V |
| Dg17 | chr6 | 37138350 |  | G | A | PIM1 | 17 | NM_001243186.1 | G91E |
| Dg17 | chr6 | 37138427 |  | G | T | PIM1 | 17 | NM_001243186.1 | A117S |
| Dg17 | chr6 | 37138433 |  | G | A | PIM1 | 17 | NM_001243186.1 | G119S |
| Dg17 | chr6 | 37138549 |  | G | A | PIM1 | 17 | NM_001243186.1 | G119D |
| Dg17 | chr6 | 37138554 |  | G | T | PIM1 | 17 | NM_001243186.1 | E121* |
| Dg17 | chr6 | 37138563 |  | C | A | PIM1 | 17 | NM_001243186.1 | P124T |
| Dg17 | chr6 | 37138577 |  | G | C | PIM1 | 17 | NM_001243186.1 | Q128H |
| Dg17 | chr6 | 37138649 |  | C | G | PIM1 | 17 | NM_001243186.1 | N152K |
| Dg17 | chr6 | 37138653 |  | C | T | PIM1 | 17 | NM_001243186.1 | P154S |
| Dg17 | chr6 | 37138769 |  | C | T | PIM1 | 17 | NM_001243186.1 | H159Y |
| Dg17 | chr6 | 37138796 |  | TG | T | PIM1 | 17 | NM_001243186.1 | W168 |
| Dg17 | chr6 | 37138805 |  | C | G | PIM1 | 17 | NM_001243186.1 | L171V |
| Dg17 | chr6 | 37138901 |  | C | T | PIM1 | 17 | NM_001243186.1 | P172S |
| Dg17 | chr6 | 37138950 |  | G | A | PIM1 | 17 | NM_001243186.1 | S188N |
| Dg17 | chr6 | 37138951 |  | C | G | PIM1 | 17 | NM_001243186.1 | S188R |
| Dg17 | chr6 | 37138956 |  | G | A | PIM1 | 17 | NM_001243186.1 | G190D |
| Dg17 | chr6 | 37139036 |  | G | A | PIM1 | 17 | NM_001243186.1 | V217M |
| Dg17 | chr6 | 37139063 |  | G | A | PIM1 | 17 | NM_001243186.1 | E226K |
| Dg17 | chr6 | 37140894 |  | C | T | PIM1 | 17 | NM_001243186.1 | H335Y |
| Dg18 | chr6 | 37138901 |  | C | T | PIM1 | 17 | NM_001243186.1 | P172S |
| Dg18 | chr6 | 37139012 |  | C | G | PIM1 | 17 | NM_001243186.1 | L209V |
| Dg20 | chr6 | 37138091 |  | C | T | PIM1 | 17 | NM_001243186.1 | P5S |
| Dg20 | chr6 | 37138107 |  | C | G | PIM1 | 17 | NM_001243186.1 | T10S |
| Dg20 | chr6 | 37138247 |  | A | T | PIM1 | 17 | NM_001243186.1 | S57C |
| Dg20 | chr6 | 37138296 |  | G | A | PIM1 | 17 | NM_001243186.1 | G73D |
| Dg20 | chr6 | 37138352 |  | A | G | PIM1 | 17 | NM_002648.3 | M1V |
| Dg20 | chr6 | 37138355 |  | C | G | PIM1 | 17 | NM_001243186.1 | L93V |
| Dg20 | chr6 | 37138554 |  | G | A | PIM1 | 17 | NM_001243186.1 | E121K |
| Dg20 | chr6 | 37138563 |  | C | G | PIM1 | 17 | NM_001243186.1 | P124A |
| Dg20 | chr6 | 37138652 |  | G | C | PIM1 | 17 | NM_001243186.1 | L153F |
| Dg20 | chr6 | 37138901 |  | C | G | PIM1 | 17 | NM_001243186.1 | P172A |
| Dg20 | chr6 | 37139039 |  | C | T | PIM1 | 17 | NM_001243186.1 | Q218* |
| Dg20 | chr6 | 37139084 |  | G | C | PIM1 | 17 | NM_001243186.1 | E233Q |
| Dg20 | chr6 | 37139086 |  | G | T | PIM1 | 17 | NM_001243186.1 | E233D |
| Dg21 | chr6 | 37138423 |  | G | C | PIM1 | 17 | NM_001243186.1 | K115N |
| Dg21 | chr6 | 37138549 |  | G | T | PIM1 | 17 | NM_001243186.1 | G119V |
| Dg26 | chr6 | 37138293 |  | C | G | PIM1 | 17 | NM_001243186.1 | S72C |
| Dg26 | chr6 | 37138355 |  | C | T | PIM1 | 17 | NM_001243186.1 | L93F |
| Dg26 | chr6 | 37138374 |  | C | T | PIM1 | 17 | NM_001243186.1 | S99L |
| Dg26 | chr6 | 37138423 |  | G | C | PIM1 | 17 | NM_001243186.1 | K115N |
| Dg26 | chr6 | 37138549 |  | G | A | PIM1 | 17 | NM_001243186.1 | G119D |
| Dg26 | chr6 | 37138553 |  | G | C | PIM1 | 17 | NM_001243186.1 | K120N |
| Dg26 | chr6 | 37138563 |  | C | T | PIM1 | 17 | NM_001243186.1 | P124S |
| Dg26 | chr6 | 37138630 |  | G | A | PIM1 | 17 | NM_001243186.1 | G146D |
| Dg26 | chr6 | 37138649 |  | C | G | PIM1 | 17 | NM_001243186.1 | N152K |
| Dg26 | chr6 | 37138928 |  | G | A | PIM1 | 17 | NM_001243186.1 | V181M |
| Dg26 | chr6 | 37138950 |  | G | C | PIM1 | 17 | NM_001243186.1 | S188T |
| Dg26 | chr6 | 37139039 |  | C | T | PIM1 | 17 | NM_001243186.1 | Q218* |
| Dg26 | chr6 | 37139063 |  | G | A | PIM1 | 17 | NM_001243186.1 | E226K |
| Dg26 | chr6 | 37139107 |  | G | A | PIM1 | 17 | NM_001243186.1 | W240* |
| Dg26 | chr6 | 37139120 |  | GCCGTGCGGCACTGCCA | G | PIM1 | 17 | NM_001243186.1 | AVRHCH245 |
| Dg26 | chr6 | 37139210 |  | C | T | PIM1 | 17 | NM_001243186.1 | L275F |
| Dg26 | chr6 | 37139237 |  | C | T | PIM1 | 17 | NM_001243186.1 | L284F |
| Dg26 | chr6 | 37140886 |  | C | T | PIM1 | 17 | NM_001243186.1 | P332L |
| Dp01 | chr6 | 37138308 |  | G | A | PIM1 | 17 | NM_001243186.1 | S77N |
| Dp03 | chr6 | 37138769 |  | C | G | PIM1 | 17 | NM_001243186.1 | H159D |
| Dp04 | chr6 | 37138424 |  | C | G | PIM1 | 17 | NM_001243186.1 | L116V |
| Dg01 | chr19 | 9009325 |  | A | T | MUC16 | 14 | NM_024690.2 | L13050M |
| Dg02 | chr19 | 8999518 |  | A | G | MUC16 | 14 | NM_024690.2 | Y13553H |
| Dg03 | chr19 | 8971738 |  | G | C | MUC16 | 14 | NM_024690.2 | T14285S |
| Dg05 | chr19 | 8999445 |  | C | T | MUC16 | 14 | NM_024690.2 | S13577N |
| Dg05 | chr19 | 8999498 |  | G | T | MUC16 | 14 | NM_024690.2 | S13559R |
| Dg05 | chr19 | 8999509 |  | C | T | MUC16 | 14 | NM_024690.2 | D13556N |
| Dg08 | chr19 | 9047963 |  | G | C | MUC16 | 14 | NM_024690.2 | S11223* |
| Dg12 | chr19 | 9018508 |  | T | G | MUC16 | 14 | NM_024690.2 | K12556Q |
| Dg12 | chr19 | 9061789 |  | G | T | MUC16 | 14 | NM_024690.2 | P8553T |
| Dg14 | chr19 | 9024473 |  | A | G | MUC16 | 14 | NM_024690.2 | S12354P |
| Dg14 | chr19 | 9024481 |  | C | T | MUC16 | 14 | NM_024690.2 | S12351N |
| Dg16 | chr19 | 8999443 |  | T | C | MUC16 | 14 | NM_024690.2 | I13578V |
| Dg18 | chr19 | 8999446 |  | T | C | MUC16 | 14 | NM_024690.2 | S13577G |
| Dg20 | chr19 | 8999538 |  | T | C | MUC16 | 14 | NM_024690.2 | K13546R |
| Dg20 | chr19 | 9084934 |  | T | C | MUC16 | 14 | NM_024690.2 | D2294G |
| Dg22 | chr19 | 9069489 |  | G | T | MUC16 | 14 | NM_024690.2 | A5986E |
| Dp01 | chr19 | 9009276 |  | A | G | MUC16 | 14 | NM_024690.2 | I13066T |
| Dp01 | chr19 | 9009325 |  | A | T | MUC16 | 14 | NM_024690.2 | L13050M |
| Dp01 | chr19 | 9009331 |  | C | T | MUC16 | 14 | NM_024690.2 | G13048S |
| Dp07 | chr19 | 9006657 |  | A | C | MUC16 | 14 | NM_024690.2 | S13197R |
| Dp09 | chr19 | 9008344 |  | A | G | MUC16 | 14 | NM_024690.2 | S13070P |
| Dg02 | chr7 | 100549655 |  | C | T | MUC3A | 14 | NM_005960.1 | T79I |
| Dg04 | chr7 | 100550168 |  | A | C | MUC3A | 14 | NM_005960.1 | K250T |
| Dg04 | chr7 | 100550176 |  | G | A | MUC3A | 14 | NM_005960.1 | V253M |
| Dg04 | chr7 | 100550243 |  | C | T | MUC3A | 14 | NM_005960.1 | T275I |
| Dg04 | chr7 | 100550251 |  | C | T | MUC3A | 14 | NM_005960.1 | Q278* |
| Dg05 | chr7 | 100550168 |  | A | C | MUC3A | 14 | NM_005960.1 | K250T |
| Dg07 | chr7 | 100550168 |  | A | C | MUC3A | 14 | NM_005960.1 | K250T |
| Dg08 | chr7 | 100550176 |  | G | A | MUC3A | 14 | NM_005960.1 | V253M |
| Dg10 | chr7 | 100550176 |  | G | A | MUC3A | 14 | NM_005960.1 | V253M |
| Dg10 | chr7 | 100551208 |  | G | A | MUC3A | 14 | NM_005960.1 | E597K |
| Dg15 | chr7 | 100550432 |  | C | T | MUC3A | 14 | NM_005960.1 | T338I |
| Dg15 | chr7 | 100551122 |  | T | G | MUC3A | 14 | NM_005960.1 | I568S |
| Dg22 | chr7 | 100550399 |  | G | C | MUC3A | 14 | NM_005960.1 | R327T |
| Dg22 | chr7 | 100550957 |  | C | G | MUC3A | 14 | NM_005960.1 | T513S |
| Dg22 | chr7 | 100551130 |  | G | A | MUC3A | 14 | NM_005960.1 | G571S |
| Dg25 | chr7 | 100550384 |  | C | T | MUC3A | 14 | NM_005960.1 | P322L |
| Dp01 | chr7 | 100550168 |  | A | C | MUC3A | 14 | NM_005960.1 | K250T |
| Dp01 | chr7 | 100550176 |  | G | A | MUC3A | 14 | NM_005960.1 | V253M |
| Dp01 | chr7 | 100551133 |  | C | T | MUC3A | 14 | NM_005960.1 | L572F |
| Dp03 | chr7 | 100550168 |  | A | C | MUC3A | 14 | NM_005960.1 | K250T |
| Dp03 | chr7 | 100550176 |  | G | A | MUC3A | 14 | NM_005960.1 | V253M |
| Dp05 | chr7 | 100550168 |  | A | C | MUC3A | 14 | NM_005960.1 | K250T |
| Dp05 | chr7 | 100550176 |  | G | A | MUC3A | 14 | NM_005960.1 | V253M |
| Dp06 | chr7 | 100551008 |  | C | T | MUC3A | 14 | NM_005960.1 | T530I |
| Dp09 | chr7 | 100549537 |  | G | A | MUC3A | 14 | NM_005960.1 | A40T |
| Dp09 | chr7 | 100549540 |  | A | C | MUC3A | 14 | NM_005960.1 | S41R |
| Dp09 | chr7 | 100549543 |  | G | A | MUC3A | 14 | NM_005960.1 | A42T |
| Dp09 | chr7 | 100549547 |  | T | C | MUC3A | 14 | NM_005960.1 | V43A |
| Dg03 | chr11 | 1017591 |  | C | G | MUC6 | 14 | NM_005961.2 | R1737P |
| Dg07 | chr11 | 1017592 |  | G | C | MUC6 | 14 | NM_005961.2 | R1737G |
| Dg10 | chr11 | 1017592 |  | G | C | MUC6 | 14 | NM_005961.2 | R1737G |
| Dg10 | chr11 | 1018246 |  | T | C | MUC6 | 14 | NM_005961.2 | N1519D |
| Dg13 | chr11 | 1018341 |  | G | A | MUC6 | 14 | NM_005961.2 | P1487L |
| Dg13 | chr11 | 1018390 |  | C | A | MUC6 | 14 | NM_005961.2 | A1471S |
| Dg16 | chr11 | 1018390 |  | C | A | MUC6 | 14 | NM_005961.2 | A1471S |
| Dg17 | chr11 | 1018419 |  | G | A | MUC6 | 14 | NM_005961.2 | T1461I |
| Dg20 | chr11 | 1016910 |  | G | A | MUC6 | 14 | NM_005961.2 | T1964I |
| Dg21 | chr11 | 1017573 |  | G | C | MUC6 | 14 | NM_005961.2 | A1743G |
| Dg21 | chr11 | 1017623 |  | C | G | MUC6 | 14 | NM_005961.2 | M1726I |
| Dg21 | chr11 | 1018255 |  | A | T | MUC6 | 14 | NM_005961.2 | F1516I |
| Dg21 | chr11 | 1018275 |  | G | A | MUC6 | 14 | NM_005961.2 | T1509I |
| Dg25 | chr11 | 1017529 |  | G | A | MUC6 | 14 | NM_005961.2 | H1758Y |
| Dg25 | chr11 | 1017573 |  | G | C | MUC6 | 14 | NM_005961.2 | A1743G |
| Dg25 | chr11 | 1017597 |  | T | G | MUC6 | 14 | NM_005961.2 | Q1735P |
| Dg26 | chr11 | 1018245 |  | T | G | MUC6 | 14 | NM_005961.2 | N1519T |
| Dg26 | chr11 | 1018246 |  | T | C | MUC6 | 14 | NM_005961.2 | N1519D |
| Dp01 | chr11 | 1017592 |  | G | C | MUC6 | 14 | NM_005961.2 | R1737G |
| Dp01 | chr11 | 1018456 |  | G | A | MUC6 | 14 | NM_005961.2 | P1449S |
| Dp03 | chr11 | 1017988 |  | G | T | MUC6 | 14 | NM_005961.2 | P1605T |
| Dp05 | chr11 | 1016581 |  | T | C | MUC6 | 14 | NM_005961.2 | T2074A |
| Dp05 | chr11 | 1018456 |  | G | A | MUC6 | 14 | NM_005961.2 | P1449S |
| Dp06 | chr11 | 1016914 |  | G | T | MUC6 | 14 | NM_005961.2 | P1963T |
| Dp06 | chr11 | 1017592 |  | G | C | MUC6 | 14 | NM_005961.2 | R1737G |
| Dg04 | chr13 | 25670691 |  | G | T | PABPC3 | 13 | NM_030979.2 | V119F |
| Dg04 | chr13 | 25670780 |  | C | G | PABPC3 | 13 | NM_030979.2 | H148Q |
| Dg04 | chr13 | 25670797 |  | C | G | PABPC3 | 13 | NM_030979.2 | A154G |
| Dg04 | chr13 | 25670803 |  | A | G | PABPC3 | 13 | NM_030979.2 | K156R |
| Dg04 | chr13 | 25671672 |  | A | G | PABPC3 | 13 | NM_030979.2 | S446G |
| Dg04 | chr13 | 25671679 |  | T | C | PABPC3 | 13 | NM_030979.2 | I448T |
| Dg04 | chr13 | 25671688 |  | G | C | PABPC3 | 13 | NM_030979.2 | G451A |
| Dg08 | chr13 | 25671688 |  | G | C | PABPC3 | 13 | NM_030979.2 | G451A |
| Dg10 | chr13 | 25670703 |  | G | T | PABPC3 | 13 | NM_030979.2 | G123C |
| Dg10 | chr13 | 25670712 |  | C | G | PABPC3 | 13 | NM_030979.2 | L126V |
| Dg10 | chr13 | 25670767 |  | A | G | PABPC3 | 13 | NM_030979.2 | H144R |
| Dg10 | chr13 | 25671672 |  | A | G | PABPC3 | 13 | NM_030979.2 | S446G |
| Dg10 | chr13 | 25671679 |  | T | C | PABPC3 | 13 | NM_030979.2 | I448T |
| Dg11 | chr13 | 25671672 |  | A | G | PABPC3 | 13 | NM_030979.2 | S446G |
| Dg11 | chr13 | 25671679 |  | T | C | PABPC3 | 13 | NM_030979.2 | I448T |
| Dg12 | chr13 | 25670953 |  | G | A | PABPC3 | 13 | NM_030979.2 | R206H |
| Dg12 | chr13 | 25670955 |  | C | T | PABPC3 | 13 | NM_030979.2 | L207F |
| Dg16 | chr13 | 25671679 |  | T | C | PABPC3 | 13 | NM_030979.2 | I448T |
| Dg17 | chr13 | 25671679 |  | T | C | PABPC3 | 13 | NM_030979.2 | I448T |
| Dg17 | chr13 | 25671795 |  | C | T | PABPC3 | 13 | NM_030979.2 | R487C |
| Dg22 | chr13 | 25671742 |  | G | A | PABPC3 | 13 | NM_030979.2 | R469Q |
| Dg22 | chr13 | 25671759 |  | C | T | PABPC3 | 13 | NM_030979.2 | R475C |
| Dg22 | chr13 | 25671795 |  | C | T | PABPC3 | 13 | NM_030979.2 | R487C |
| Dg23 | chr13 | 25670907 |  | C | A | PABPC3 | 13 | NM_030979.2 | P191T |
| Dg24 | chr13 | 25671795 |  | C | T | PABPC3 | 13 | NM_030979.2 | R487C |
| Dp03 | chr13 | 25670723 |  | C | A | PABPC3 | 13 | NM_030979.2 | N129K |
| Dp05 | chr13 | 25671672 |  | A | G | PABPC3 | 13 | NM_030979.2 | S446G |
| Dp05 | chr13 | 25671679 |  | T | C | PABPC3 | 13 | NM_030979.2 | I448T |
| Dp05 | chr13 | 25671688 |  | G | C | PABPC3 | 13 | NM_030979.2 | G451A |
| Dp09 | chr13 | 25671210 |  | C | T | PABPC3 | 13 | NM_030979.2 | Q292* |
| Dg03 | chr11 | 47647265 |  | A | G | MTCH2 | 11 | NM_014342.3 | F237S |
| Dg05 | chr11 | 47663948 |  | C | T | MTCH2 | 11 | NM_014342.3 | V24M |
| Dg05 | chr11 | 47663951 |  | A | G | MTCH2 | 11 | NM_014342.3 | Y23H |
| Dg07 | chr11 | 47644257 |  | T | C | MTCH2 | 11 | NM_014342.3 | K274R |
| Dg07 | chr11 | 47644270 |  | A | C | MTCH2 | 11 | NM_014342.3 | C270G |
| Dg07 | chr11 | 47644271 |  | C | T | MTCH2 | 11 | NM_014342.3 | W269* |
| Dg07 | chr11 | 47660294 |  | C | T | MTCH2 | 11 | NM_014342.3 | C79Y |
| Dg07 | chr11 | 47660295 |  | A | G | MTCH2 | 11 | NM_014342.3 | C79R |
| Dg08 | chr11 | 47647238 |  | A | G | MTCH2 | 11 | NM_014342.3 | V246A |
| Dg11 | chr11 | 47644257 |  | T | C | MTCH2 | 11 | NM_014342.3 | K274R |
| Dg11 | chr11 | 47644270 |  | A | C | MTCH2 | 11 | NM_014342.3 | C270G |
| Dg14 | chr11 | 47647238 |  | A | G | MTCH2 | 11 | NM_014342.3 | V246A |
| Dg17 | chr11 | 47660294 |  | C | T | MTCH2 | 11 | NM_014342.3 | C79Y |
| Dg17 | chr11 | 47660295 |  | A | G | MTCH2 | 11 | NM_014342.3 | C79R |
| Dg17 | chr11 | 47660301 |  | T | A | MTCH2 | 11 | NM_014342.3 | R77* |
| Dg18 | chr11 | 47647238 |  | A | G | MTCH2 | 11 | NM_014342.3 | V246A |
| Dg20 | chr11 | 47647265 |  | A | G | MTCH2 | 11 | NM_014342.3 | F237S |
| Dg22 | chr11 | 47660294 |  | C | T | MTCH2 | 11 | NM_014342.3 | C79Y |
| Dg22 | chr11 | 47660295 |  | A | G | MTCH2 | 11 | NM_014342.3 | C79R |
| Dg22 | chr11 | 47660301 |  | T | A | MTCH2 | 11 | NM_014342.3 | R77* |
| Dp05 | chr11 | 47647238 |  | A | G | MTCH2 | 11 | NM_014342.3 | V246A |
| Dp05 | chr11 | 47647265 |  | A | G | MTCH2 | 11 | NM_014342.3 | F237S |
| Dp05 | chr11 | 47663959 |  | G | C | MTCH2 | 11 | NM_014342.3 | P20R |
| Dg01 | chr6 | 26157198 |  | G | A | HIST1H1E | 10 | NM_005321.2 | V194I |
| Dg02 | chr6 | 26156985 |  | G | A | HIST1H1E | 10 | NM_005321.2 | A123T |
| Dg02 | chr6 | 26157127 |  | C | T | HIST1H1E | 10 | NM_005321.2 | A170V |
| Dg07 | chr6 | 26156827 |  | G | C | HIST1H1E | 10 | NM_005321.2 | G70A |
| Dg07 | chr6 | 26157009 |  | C | T | HIST1H1E | 10 | NM_005321.2 | P131S |
| Dg07 | chr6 | 26157090 |  | G | C | HIST1H1E | 10 | NM_005321.2 | A158P |
| Dg09 | chr6 | 26157039 |  | G | A | HIST1H1E | 10 | NM_005321.2 | A141T |
| Dg13 | chr6 | 26156650 |  | C | T | HIST1H1E | 10 | NM_005321.2 | A11V |
| Dg13 | chr6 | 26156928 |  | TC | T | HIST1H1E | 10 | NM_005321.2 | S104 |
| Dg13 | chr6 | 26156979 |  | AAAAAG | A | HIST1H1E | 10 | NM_005321.2 | KK121 |
| Dg17 | chr6 | 26157012 |  | G | C | HIST1H1E | 10 | NM_005321.2 | A132P |
| Dg17 | chr6 | 26157067 |  | G | A | HIST1H1E | 10 | NM_005321.2 | S150N |
| Dg17 | chr6 | 26157109 |  | C | G | HIST1H1E | 10 | NM_005321.2 | A164G |
| Dg20 | chr6 | 26156985 |  | G | A | HIST1H1E | 10 | NM_005321.2 | A123T |
| Dg23 | chr6 | 26157112 |  | C | T | HIST1H1E | 10 | NM_005321.2 | A165V |
| Dg26 | chr6 | 26157114 |  | G | A | HIST1H1E | 10 | NM_005321.2 | G166R |
| Dp08 | chr6 | 26156811 |  | G | A | HIST1H1E | 10 | NM_005321.2 | A65T |
| Dg07 | chr12 | 49422719 |  | A | T | KMT2D | 10 | NM_003482.3 | Y4758* |
| Dg09 | chr12 | 49416417 |  | G | A | KMT2D | 10 | NM_003482.3 | R5432W |
| Dg12 | chr12 | 49444930 |  | GC | G | KMT2D | 10 | NM_003482.3 | R845 |
| Dg17 | chr12 | 49431937 |  | C | A | KMT2D | 10 | NM_003482.3 | E3068* |
| Dg17 | chr12 | 49448759 |  | G | A | KMT2D | 10 | NM_003482.3 | P34S |
| Dg18 | chr12 | 49420640 |  | C | A | KMT2D | 10 | NM_003482.3 | E5037* |
| Dg18 | chr12 | 49440496 |  | G | T | KMT2D | 10 | NM_003482.3 | D1438E |
| Dg23 | chr12 | 49432566 |  | G | GA | KMT2D | 10 | NM_003482.3 | S2858F? |
| Dg23 | chr12 | 49433509 |  | G | A | KMT2D | 10 | NM_003482.3 | Q2682* |
| Dg26 | chr12 | 49431956 |  | GT | G | KMT2D | 10 | NM_003482.3 | D3061 |
| Dg26 | chr12 | 49431958 |  | C | G | KMT2D | 10 | NM_003482.3 | D3061H |
| Dp01 | chr12 | 49427687 |  | G | A | KMT2D | 10 | NM_003482.3 | Q3601* |
| Dp01 | chr12 | 49446461 |  | TG | T | KMT2D | 10 | NM_003482.3 | P381 |
| Dp04 | chr12 | 49416087 |  | G | A | KMT2D | 10 | NM_003482.3 | A5463V |
| Dp05 | chr12 | 49420578 |  | C | T | KMT2D | 10 | NM_003482.3 | W5057* |
| Dp05 | chr12 | 49432147 |  | C | CA | KMT2D | 10 | NM_003482.3 | F2997F? |
| Dg02 | chr10 | 126686726 |  | G | T | CTBP2 | 9 | NM_001083914.1 | C124* |
| Dg05 | chr10 | 126686682 |  | A | T | CTBP2 | 9 | NM_001083914.1 | I139N |
| Dg05 | chr10 | 126686682 |  | A | G | CTBP2 | 9 | NM_001083914.1 | I139T |
| Dg07 | chr10 | 126686680 |  | A | C | CTBP2 | 9 | NM_001083914.1 | C140G |
| Dg08 | chr10 | 126686629 |  | G | A | CTBP2 | 9 | NM_001083914.1 | R157W |
| Dg08 | chr10 | 126686646 |  | C | G | CTBP2 | 9 | NM_001083914.1 | W151S |
| Dg08 | chr10 | 126686649 |  | G | C | CTBP2 | 9 | NM_001083914.1 | T150R |
| Dg08 | chr10 | 126686656 |  | T | C | CTBP2 | 9 | NM_001083914.1 | R148G |
| Dg09 | chr10 | 126686682 |  | A | T | CTBP2 | 9 | NM_001083914.1 | I139N |
| Dg09 | chr10 | 126686682 |  | A | G | CTBP2 | 9 | NM_001083914.1 | I139T |
| Dg19 | chr10 | 126686682 |  | A | T | CTBP2 | 9 | NM_001083914.1 | I139N |
| Dg19 | chr10 | 126686682 |  | A | G | CTBP2 | 9 | NM_001083914.1 | I139T |
| Dg21 | chr10 | 126686646 |  | C | G | CTBP2 | 9 | NM_001083914.1 | W151S |
| Dg21 | chr10 | 126686649 |  | G | C | CTBP2 | 9 | NM_001083914.1 | T150R |
| Dg21 | chr10 | 126686656 |  | T | C | CTBP2 | 9 | NM_001083914.1 | R148G |
| Dg21 | chr10 | 126686659 |  | G | A | CTBP2 | 9 | NM_001083914.1 | R147W |
| Dg24 | chr10 | 126683099 |  | G | A | CTBP2 | 9 | NM_001083914.1 | S240F |
| Dg24 | chr10 | 126683111 |  | C | T | CTBP2 | 9 | NM_001083914.1 | S236N |
| Dg24 | chr10 | 126683243 |  | C | T | CTBP2 | 9 | NM_001083914.1 | G192E |
| Dg24 | chr10 | 126683244 |  | C | G | CTBP2 | 9 | NM_001083914.1 | G192R |
| Dg24 | chr10 | 126686680 |  | A | C | CTBP2 | 9 | NM_001083914.1 | C140G |
| Dg24 | chr10 | 126686692 |  | C | T | CTBP2 | 9 | NM_001083914.1 | D136N |
| Dg24 | chr10 | 126692029 |  | G | T | CTBP2 | 9 | NM_001083914.1 | H31N |
| Dg24 | chr10 | 126692035 |  | G | T | CTBP2 | 9 | NM_001083914.1 | P29T |
| Dg24 | chr10 | 126692037 |  | C | A | CTBP2 | 9 | NM_001083914.1 | G28V |
| Dg24 | chr10 | 126692039 |  | G | T | CTBP2 | 9 | NM_001083914.1 | N27K |
| Dg26 | chr10 | 126686629 |  | G | A | CTBP2 | 9 | NM_001083914.1 | R157W |
| Dg02 | chr6 | 29910609 |  | G | A | HLA-A | 9 | NM_001242758.1 | G50D |
| Dg05 | chr6 | 29910693 |  | A | G | HLA-A | 9 | NM_001242758.1 | Q78R |
| Dg08 | chr6 | 29910594 |  | G | A | HLA-A | 9 | NM_001242758.1 | R45H |
| Dg13 | chr6 | 29910692 |  | C | T | HLA-A | 9 | NM_001242758.1 | Q78* |
| Dg20 | chr6 | 29910617 |  | G | A | HLA-A | 9 | NM_001242758.1 | D53N |
| Dg20 | chr6 | 29910693 |  | A | G | HLA-A | 9 | NM_001242758.1 | Q78R |
| Dg25 | chr6 | 29911072 |  | G | A | HLA-A | 9 | NM_001242758.1 | G124D |
| Dg26 | chr6 | 29910692 |  | C | T | HLA-A | 9 | NM_001242758.1 | Q78* |
| Dg26 | chr6 | 29911047 |  | TCTCACACCATCCAGATA | T | HLA-A | 9 | NM_001242758.1 | SHTIQI116 |
| Dp02 | chr6 | 29910795 |  | GC | G | HLA-A | 9 | NM_001242758.1 | S112 |
| Dp04 | chr6 | 29910602 |  | G | C | HLA-A | 9 | NM_001242758.1 | A48P |
| Dg02 | chr3 | 38182292 |  | G | A | MYD88 | 9 | NM_002468.4 | S243A |
| Dg03 | chr3 | 38182641 |  | T | C | MYD88 | 9 | NM_002468.4 | L265P |
| Dg05 | chr3 | 38182641 |  | T | C | MYD88 | 9 | NM_002468.4 | L265P |
| Dg08 | chr3 | 38182025 |  | G | T | MYD88 | 9 | NM_002468.4 | V217T |
| Dg10 | chr3 | 38182025 |  | G | T | MYD88 | 9 | NM_002468.4 | V217T |
| Dg18 | chr3 | 38182641 |  | T | C | MYD88 | 9 | NM_002468.4 | L265P |
| Dg19 | chr3 | 38182292 |  | G | A | MYD88 | 9 | NM_002468.4 | S243A |
| Dg26 | chr3 | 38182641 |  | T | C | MYD88 | 9 | NM_002468.4 | L265P |
| Dp02 | chr3 | 38182630 |  | TCAGAAG | T | MYD88 | 9 | NM_002468.4 | HQK261H |
| Dg02 | chr3 | 32022427 |  | C | G | OSBPL10 | 9 | NM_001174060.1 | S82T |
| Dg02 | chr3 | 32022608 |  | T | A | OSBPL10 | 9 | NM_001174060.1 | S22C |
| Dg06 | chr3 | 32022625 |  | C | T | OSBPL10 | 9 | NM_001174060.1 | S16N |
| Dg09 | chr3 | 32022635 |  | C | T | OSBPL10 | 9 | NM_001174060.1 | G13S |
| Dg10 | chr3 | 32022510 |  | G | T | OSBPL10 | 9 | NM_001174060.1 | S54R |
| Dg13 | chr3 | 32022601 |  | G | A | OSBPL10 | 9 | NM_001174060.1 | A24V |
| Dg17 | chr3 | 32022622 |  | C | T | OSBPL10 | 9 | NM_001174060.1 | S17N |
| Dg18 | chr3 | 32022500 |  | GGCTGCTGCGGCTT | G | OSBPL10 | 9 | NM_001174060.1 | GSRSS53 |
| Dg18 | chr3 | 32022602 |  | C | T | OSBPL10 | 9 | NM_001174060.1 | A24T |
| Dg20 | chr3 | 32022625 |  | C | T | OSBPL10 | 9 | NM_001174060.1 | S16N |
| Dg26 | chr3 | 32022391 |  | C | T | OSBPL10 | 9 | NM_001174060.1 | R94K |
| Dg26 | chr3 | 32022413 |  | G | A | OSBPL10 | 9 | NM_001174060.1 | L87F |
| Dg26 | chr3 | 32022422 |  | A | T | OSBPL10 | 9 | NM_001174060.1 | Y84N |
| Dg26 | chr3 | 32022426 |  | G | C | OSBPL10 | 9 | NM_001174060.1 | S82R |
| Dg26 | chr3 | 32022431 |  | G | C | OSBPL10 | 9 | NM_001174060.1 | L81V |
| Dg26 | chr3 | 32022625 |  | C | T | OSBPL10 | 9 | NM_001174060.1 | S16N |
| Dg05 | chr17 | 62006799 |  | A | G | CD79B | 8 | NM_000626.2 | Y196H |
| Dg08 | chr17 | 62007200 |  | C | A | CD79B | 8 | NM_000626.2 | G160V |
| Dg09 | chr17 | 62006798 |  | T | C | CD79B | 8 | NM_000626.2 | Y196C |
| Dg09 | chr17 | 62006799 |  | A | G | CD79B | 8 | NM_000626.2 | Y196H |
| Dg17 | chr17 | 62006674 |  | A | T | CD79B | 8 | NM_000626.2 | I201N |
| Dg17 | chr17 | 62006799 |  | A | C | CD79B | 8 | NM_000626.2 | Y196D |
| Dg18 | chr17 | 62006799 |  | A | G | CD79B | 8 | NM_000626.2 | Y196H |
| Dg19 | chr17 | 62006600 |  | G | C | CD79B | 8 | NM_000626.2 | P226A |
| Dg19 | chr17 | 62006604 |  | C | G | CD79B | 8 | NM_000626.2 | E224D |
| Dg19 | chr17 | 62006799 |  | A | T | CD79B | 8 | NM_000626.2 | Y196N |
| Dg19 | chr17 | 62007190 |  | C | T | CD79B | 8 | NM_000626.2 | M163I |
| Dg26 | chr17 | 62006798 |  | T | C | CD79B | 8 | NM_000626.2 | Y196C |
| Dp02 | chr17 | 62006799 |  | A | G | CD79B | 8 | NM_000626.2 | Y196H |
| Dp02 | chr17 | 62008687 |  | CTACTGACCTTTGGGATTCCGG | C | CD79B | 8 | NM_000626.2 | YRNPKG35 |
| Dg08 | chr2 | 96810898 |  | C | T | DUSP2 | 8 | NM_004418.3 | V66I |
| Dg14 | chr2 | 96810003 |  | G | A | DUSP2 | 8 | NM_004418.3 | A207V |
| Dg14 | chr2 | 96810529 |  | A | G | DUSP2 | 8 | NM_004418.3 | S161P |
| Dg14 | chr2 | 96810589 |  | C | T | DUSP2 | 8 | NM_004418.3 | D141N |
| Dg17 | chr2 | 96809989 |  | G | A | DUSP2 | 8 | NM_004418.3 | H212Y |
| Dg17 | chr2 | 96810946 |  | G | A | DUSP2 | 8 | NM_004418.3 | P50S |
| Dg17 | chr2 | 96811017 |  | C | T | DUSP2 | 8 | NM_004418.3 | R26H |
| Dg20 | chr2 | 96809662 |  | T | G | DUSP2 | 8 | NM_004418.3 | E282A |
| Dg20 | chr2 | 96809766 |  | C | G | DUSP2 | 8 | NM_004418.3 | K247N |
| Dg20 | chr2 | 96810000 |  | C | G | DUSP2 | 8 | NM_004418.3 | S208T |
| Dg20 | chr2 | 96810054 |  | T | C | DUSP2 | 8 | NM_004418.3 | D190G |
| Dg20 | chr2 | 96810504 |  | T | G | DUSP2 | 8 | NM_004418.3 | D169A |
| Dg20 | chr2 | 96810549 |  | G | C | DUSP2 | 8 | NM_004418.3 | T154R |
| Dg20 | chr2 | 96810556 |  | G | A | DUSP2 | 8 | NM_004418.3 | P152S |
| Dg20 | chr2 | 96810568 |  | C | G | DUSP2 | 8 | NM_004418.3 | A148P |
| Dg20 | chr2 | 96810582 |  | C | T | DUSP2 | 8 | NM_004418.3 | C143Y |
| Dg20 | chr2 | 96810600 |  | C | T | DUSP2 | 8 | NM_004418.3 | G137D |
| Dg20 | chr2 | 96810609 |  | C | T | DUSP2 | 8 | NM_004418.3 | G134D |
| Dg20 | chr2 | 96810618 |  | C | G | DUSP2 | 8 | NM_004418.3 | G131A |
| Dg20 | chr2 | 96810712 |  | G | C | DUSP2 | 8 | NM_004418.3 | L128V |
| Dg20 | chr2 | 96810762 |  | A | G | DUSP2 | 8 | NM_004418.3 | L111P |
| Dg20 | chr2 | 96810828 |  | A | T | DUSP2 | 8 | NM_004418.3 | V89E |
| Dg20 | chr2 | 96810841 |  | G | C | DUSP2 | 8 | NM_004418.3 | L85V |
| Dg20 | chr2 | 96810895 |  | G | C | DUSP2 | 8 | NM_004418.3 | L67V |
| Dg21 | chr2 | 96810909 |  | G | A | DUSP2 | 8 | NM_004418.3 | P62L |
| Dg25 | chr2 | 96810582 |  | C | T | DUSP2 | 8 | NM_004418.3 | C143Y |
| Dg25 | chr2 | 96810768 |  | A | G | DUSP2 | 8 | NM_004418.3 | V109A |
| Dg26 | chr2 | 96810549 |  | G | A | DUSP2 | 8 | NM_004418.3 | T154I |
| Dp04 | chr2 | 96810556 |  | G | A | DUSP2 | 8 | NM_004418.3 | P152S |
| Dp04 | chr2 | 96810579 |  | G | T | DUSP2 | 8 | NM_004418.3 | S144Y |
| Dg05 | chr17 | 63049826 |  | C | T | GNA13 | 8 | NM_001282425.1 | A7T |
| Dg13 | chr17 | 63010835 |  | C | T | GNA13 | 8 | NM_001282425.1 | G130D |
| Dg20 | chr17 | 63010536 |  | A | AT | GNA13 | 8 | NM_001282425.1 | K229K? |
| Dg21 | chr17 | 63010893 |  | T | A | GNA13 | 8 | NM_001282425.1 | I111F |
| Dg25 | chr17 | 63052631 |  | C | A | GNA13 | 8 | NM_006572.5 | Q27H |
| Dp01 | chr17 | 63049759 |  | G | GACATCTAT | GNA13 | 8 | NM_001282425.1 | S29YRC? |
| Dp04 | chr17 | 63052630 |  | G | A | GNA13 | 8 | NM_006572.5 | Q28* |
| Dp09 | chr17 | 63010709 |  | T | A | GNA13 | 8 | NM_001282425.1 | E172V |
| Dp09 | chr17 | 63049822 |  | CGA | C | GNA13 | 8 | NM_001282425.1 | AR7 |
| Dg01 | chr17 | 7578190 |  | T | C | TP53 | 8 | NM_000546.5 | Y220C |
| Dg19 | chr17 | 7578388 |  | C | T | TP53 | 8 | NM_000546.5 | R181H |
| Dg20 | chr17 | 7577085 |  | C | T | TP53 | 8 | NM_000546.5 | E285K |
| Dg20 | chr17 | 7577097 |  | C | G | TP53 | 8 | NM_000546.5 | D281H |
| Dp01 | chr17 | 7578429 |  | CTG | C | TP53 | 8 | NM_000546.5 | Q167 |
| Dp02 | chr17 | 7577105 |  | G | A | TP53 | 8 | NM_000546.5 | P278L |
| Dp04 | chr17 | 7578407 |  | G | C | TP53 | 8 | NM_000546.5 | R175G |
| Dp05 | chr17 | 7578203 |  | C | T | TP53 | 8 | NM_000546.5 | V216M |
| Dp08 | chr17 | 7577561 |  | ACT | A | TP53 | 8 | NM_000546.5 | S240 |
| Dg01 | chr2 | 168106192 |  | C | A | XIRP2 | 8 | NM_001199144.1 | Q2542K |
| Dg06 | chr2 | 168100733 |  | A | G | XIRP2 | 8 | NM_001199144.1 | D722G |
| Dg17 | chr2 | 168103460 |  | AT | A | XIRP2 | 8 | NM_001199144.1 | N1631 |
| Dg20 | chr2 | 168114624 |  | G | A | XIRP2 | 8 | NM_001079810.3 | R523K |
| Dg24 | chr2 | 168074700 |  | G | A | XIRP2 | 8 | NM_001079810.3 | A250T |
| Dg25 | chr2 | 167760317 |  | C | T | XIRP2 | 8 | NM_001079810.3 | R109C |
| Dp02 | chr2 | 168114704 |  | T | G | XIRP2 | 8 | NM_001079810.3 | L550V |
| Dp07 | chr2 | 168107662 |  | G | T | XIRP2 | 8 | NM_001199144.1 | E3032* |
| Dg05 | chr4 | 88535832 |  | A | ATAGCAGTGACAGCAGCAG | DSPP | 7 | NM_014208.3 | D673DSSDSSS |
| Dg09 | chr4 | 88535832 |  | A | ATAGCAGTGACAGCAGCAG | DSPP | 7 | NM_014208.3 | D673DSSDSSS |
| Dg11 | chr4 | 88535831 |  | G | A | DSPP | 7 | NM_014208.3 | D673N |
| Dg11 | chr4 | 88535832 |  | A | G | DSPP | 7 | NM_014208.3 | D673G |
| Dg13 | chr4 | 88535832 |  | A | ATAGCAGTGACAGCAGCAG | DSPP | 7 | NM_014208.3 | D673DSSDSSS |
| Dg18 | chr4 | 88535840 |  | G | GACAGCAGCAGTAGCAGTG | DSPP | 7 | NM_014208.3 | D676DSSSSSD |
| Dp02 | chr4 | 88535831 |  | G | A | DSPP | 7 | NM_014208.3 | D673N |
| Dp02 | chr4 | 88535832 |  | A | G | DSPP | 7 | NM_014208.3 | D673G |
| Dp04 | chr4 | 88535837 |  | A | AGTGACAGCAGCAGTAGCA | DSPP | 7 | NM_014208.3 | S675SDSSSSS |
| Dg01 | chr4 | 126389831 |  | C | T | FAT4 | 7 | NM_001291285.1 | R4024W |
| Dg05 | chr4 | 126328129 |  | G | A | FAT4 | 7 | NM_001291285.1 | R1801Q |
| Dg06 | chr4 | 126373365 |  | C | A | FAT4 | 7 | NM_001291285.1 | H3734N |
| Dg14 | chr4 | 126242585 |  | A | T | FAT4 | 7 | NM_001291285.1 | E1673D |
| Dg19 | chr4 | 126239860 |  | C | A | FAT4 | 7 | NM_001291285.1 | A765D |
| Dg23 | chr4 | 126336160 |  | C | A | FAT4 | 7 | NM_001291285.1 | Y2014* |
| Dg25 | chr4 | 126336420 |  | C | A | FAT4 | 7 | NM_001291285.1 | T2101N |
| Dg01 | chr6 | 27835016 |  | G | A | HIST1H1B | 7 | NM_005322.2 | Q98* |
| Dg02 | chr6 | 27835060 |  | A | G | HIST1H1B | 7 | NM_005322.2 | I83T |
| Dg09 | chr6 | 27834968 |  | C | T | HIST1H1B | 7 | NM_005322.2 | A114T |
| Dg12 | chr6 | 27835032 |  | G | C | HIST1H1B | 7 | NM_005322.2 | S92R |
| Dg13 | chr6 | 27835055 |  | GC | G | HIST1H1B | 7 | NM_005322.2 | K84 |
| Dg17 | chr6 | 27834980 |  | G | A | HIST1H1B | 7 | NM_005322.2 | L110F |
| Dp03 | chr6 | 27834998 |  | C | T | HIST1H1B | 7 | NM_005322.2 | A104T |
| Dg01 | chr6 | 26056280 |  | G | A | HIST1H1C | 7 | NM_005319.3 | T126I |
| Dg07 | chr6 | 26056374 |  | G | A | HIST1H1C | 7 | NM_005319.3 | Q95* |
| Dg13 | chr6 | 26056361 |  | G | A | HIST1H1C | 7 | NM_005319.3 | T99I |
| Dg17 | chr6 | 26056275 |  | G | A | HIST1H1C | 7 | NM_005319.3 | P128S |
| Dg23 | chr6 | 26056353 |  | A | C | HIST1H1C | 7 | NM_005319.3 | S102A |
| Dp03 | chr6 | 26056089 |  | C | T | HIST1H1C | 7 | NM_005319.3 | A190T |
| Dp03 | chr6 | 26056448 |  | C | T | HIST1H1C | 7 | NM_005319.3 | G70D |
| Dp05 | chr6 | 26056616 |  | G | A | HIST1H1C | 7 | NM_005319.3 | P14L |
| Dg03 | chr7 | 82585729 |  | C | G | PCLO | 7 | NM_014510.2 | E1514Q |
| Dg14 | chr7 | 82545631 |  | A | C | PCLO | 7 | NM_014510.2 | S3891A |
| Dg18 | chr7 | 82580368 |  | T | A | PCLO | 7 | NM_014510.2 | D3179V |
| Dg19 | chr7 | 82581239 |  | C | A | PCLO | 7 | NM_014510.2 | K3010N |
| Dg20 | chr7 | 82584244 |  | C | G | PCLO | 7 | NM_014510.2 | D2009H |
| Dp02 | chr7 | 82785302 |  | T | G | PCLO | 7 | NM_014510.2 | I219L |
| Dp04 | chr7 | 82390096 |  | T | A | PCLO | 7 | NM_033026.5 | L5049F |
| Dp04 | chr7 | 82390098 |  | A | T | PCLO | 7 | NM_033026.5 | L5049I |
| Dp04 | chr7 | 82508700 |  | T | G | PCLO | 7 | NM_014510.2 | K4536T |
| Dg02 | chr2 | 179395341 |  | A | C | TTN | 7 | NM_001256850.1 | F33693C |
| Dg04 | chr2 | 179425049 |  | G | T | TTN | 7 | NM_001256850.1 | P26963T |
| Dg12 | chr2 | 179481557 |  | A | G | TTN | 7 | NM_001256850.1 | L14379P |
| Dg17 | chr2 | 179419774 |  | G | A | TTN | 7 | NM_001256850.1 | T27830I |
| Dg17 | chr2 | 179604614 |  | T | A | TTN | 7 | NM_001256850.1 | K4132M |
| Dg18 | chr2 | 179430866 |  | A | T | TTN | 7 | NM_001256850.1 | L25024M |
| Dg20 | chr2 | 179497136 |  | G | C | TTN | 7 | NM_001256850.1 | I12854M |
| Dg20 | chr2 | 179589211 |  | G | A | TTN | 7 | NM_001256850.1 | T6647M |
| Dg26 | chr2 | 179589239 |  | C | T | TTN | 7 | NM_001256850.1 | G6638R |
| Dg26 | chr2 | 179605879 |  | AAGC | A | TTN | 7 | NM_001256850.1 | LL3709L |
| Dg09 | chr12 | 92538122 |  | C | T | BTG1 | 6 | NM_001731.2 | A84T |
| Dg13 | chr12 | 92538016 |  | C | T | BTG1 | 6 | NM_001731.2 | G119D |
| Dg13 | chr12 | 92538122 |  | C | A | BTG1 | 6 | NM_001731.2 | A84S |
| Dg13 | chr12 | 92538183 |  | C | G | BTG1 | 6 | NM_001731.2 | K63N |
| Dg13 | chr12 | 92538187 |  | C | T | BTG1 | 6 | NM_001731.2 | C62Y |
| Dg13 | chr12 | 92539184 |  | C | T | BTG1 | 6 | NM_001731.2 | S43N |
| Dg13 | chr12 | 92539189 |  | G | C | BTG1 | 6 | NM_001731.2 | S41R |
| Dg13 | chr12 | 92539200 |  | G | A | BTG1 | 6 | NM_001731.2 | Q38* |
| Dg13 | chr12 | 92539286 |  | G | A | BTG1 | 6 | NM_001731.2 | A9V |
| Dg14 | chr12 | 92539246 |  | G | C | BTG1 | 6 | NM_001731.2 | I22M |
| Dg20 | chr12 | 92539296 |  | T | G | BTG1 | 6 | NM_001731.2 | T6P |
| Dg20 | chr12 | 92539309 |  | C | A | BTG1 | 6 | NM_001731.2 | M1I |
| Dg21 | chr12 | 92538215 |  | T | G | BTG1 | 6 | NM_001731.2 | K53Q |
| Dp04 | chr12 | 92538040 |  | A | T | BTG1 | 6 | NM_001731.2 | V111E |
| Dp04 | chr12 | 92539184 |  | C | T | BTG1 | 6 | NM_001731.2 | S43N |
| Dp04 | chr12 | 92539203 |  | G | T | BTG1 | 6 | NM_001731.2 | L37M |
| Dg07 | chr1 | 203276418 |  | C | G | BTG2 | 6 | NM_006763.2 | S110C |
| Dg14 | chr1 | 203274761 |  | G | A | BTG2 | 6 | NM_006763.2 | M9I |
| Dg17 | chr1 | 203274754 |  | C | T | BTG2 | 6 | NM_006763.2 | T7I |
| Dg17 | chr1 | 203274826 |  | G | C | BTG2 | 6 | NM_006763.2 | S31T |
| Dg17 | chr1 | 203274842 |  | G | C | BTG2 | 6 | NM_006763.2 | K36N |
| Dg20 | chr1 | 203274762 |  | C | T | BTG2 | 6 | NM_006763.2 | L10F |
| Dg20 | chr1 | 203274843 |  | G | C | BTG2 | 6 | NM_006763.2 | V37L |
| Dg20 | chr1 | 203274844 |  | T | C | BTG2 | 6 | NM_006763.2 | V37A |
| Dg20 | chr1 | 203274845 |  | CTTCAGCGGGG | C | BTG2 | 6 | NM_006763.2 | FSGA38 |
| Dg20 | chr1 | 203274850 |  | G | C | BTG2 | 6 | NM_006763.2 | S39T |
| Dg20 | chr1 | 203276262 |  | AG | A | BTG2 | 6 | NM_006763.2 | K58 |
| Dg20 | chr1 | 203276421 |  | A | T | BTG2 | 6 | NM_006763.2 | Y111F |
| Dg20 | chr1 | 203276544 |  | A | T | BTG2 | 6 | NM_006763.2 | Y152F |
| Dg20 | chr1 | 203276562 |  | G | C | BTG2 | 6 | NM_006763.2 | S158T |
| Dg25 | chr1 | 203274808 |  | G | C | BTG2 | 6 | NM_006763.2 | R25T |
| Dg26 | chr1 | 203274867 |  | G | A | BTG2 | 6 | NM_006763.2 | A45T |
| Dg26 | chr1 | 203276325 |  | G | A | BTG2 | 6 | NM_006763.2 | S79N |
| Dg01 | chr6 | 14118183 |  | T | A | CD83 | 6 | NM_001040280.1 | Y14N |
| Dg01 | chr6 | 14118290 |  | G | A | CD83 | 6 | NM_001040280.1 | W49* |
| Dg01 | chr6 | 14118291 |  | G | A | CD83 | 6 | NM_001040280.1 | V50I |
| Dg13 | chr6 | 14118167 |  | TCTCTTTCTTGTAGCCTACAGC | T | CD83 | 6 | NM_001040280.1 | AYS13 |
| Dg13 | chr6 | 14118192 |  | G | A | CD83 | 6 | NM_001040280.1 | A17T |
| Dg13 | chr6 | 14118260 |  | G | A | CD83 | 6 | NM_001040280.1 | W39* |
| Dg14 | chr6 | 14118219 |  | G | C | CD83 | 6 | NM_001040280.1 | A26P |
| Dg17 | chr6 | 14118243 |  | C | G | CD83 | 6 | NM_001040280.1 | P34A |
| Dg20 | chr6 | 14118192 |  | G | C | CD83 | 6 | NM_001040280.1 | A17P |
| Dg20 | chr6 | 14118265 |  | C | T | CD83 | 6 | NM_001040280.1 | P41L |
| Dp04 | chr6 | 14118224 |  | C | G | CD83 | 6 | NM_001040280.1 | C27W |
| Dg02 | chr8 | 3351179 |  | C | T | CSMD1 | 6 | NM_033225.5 | G472R |
| Dg06 | chr8 | 3265703 |  | A | G | CSMD1 | 6 | NM_033225.5 | S597P |
| Dg07 | chr8 | 2823382 |  | TG | T | CSMD1 | 6 | NM_033225.5 | P3065 |
| Dg07 | chr8 | 2823384 |  | G | C | CSMD1 | 6 | NM_033225.5 | P3065A |
| Dg08 | chr8 | 2857576 |  | C | G | CSMD1 | 6 | NM_033225.5 | V2703L |
| Dg20 | chr8 | 2796244 |  | T | A | CSMD1 | 6 | NM_033225.5 | N3520Y |
| Dg20 | chr8 | 2954374 |  | C | T | CSMD1 | 6 | NM_033225.5 | D2379N |
| Dp09 | chr8 | 2965287 |  | G | A | CSMD1 | 6 | NM_033225.5 | A2263V |
| Dg01 | chr5 | 13777417 |  | C | A | DNAH5 | 6 | NM_001369.2 | R3000L |
| Dg02 | chr5 | 13714653 |  | G | A | DNAH5 | 6 | NM_001369.2 | A4329V |
| Dg06 | chr5 | 13928233 |  | A | G | DNAH5 | 6 | NM_001369.2 | Y83H |
| Dg08 | chr5 | 13788975 |  | G | A | DNAH5 | 6 | NM_001369.2 | R2833C |
| Dg20 | chr5 | 13714588 |  | T | C | DNAH5 | 6 | NM_001369.2 | T4351A |
| Dg25 | chr5 | 13870934 |  | G | A | DNAH5 | 6 | NM_001369.2 | A1259V |
| Dg01 | chr10 | 128829919 |  | G | A | DOCK1 | 6 | NM_001290223.1 | D544N |
| Dg09 | chr10 | 128810545 |  | C | T | DOCK1 | 6 | NM_001290223.1 | R370C |
| Dg15 | chr10 | 129160397 |  | C | A | DOCK1 | 6 | NM_001290223.1 | T1118K |
| Dg20 | chr10 | 128830531 |  | A | T | DOCK1 | 6 | NM_001290223.1 | K620M |
| Dg25 | chr10 | 129178368 |  | G | T | DOCK1 | 6 | NM_001290223.1 | R1233I |
| Dg25 | chr10 | 129231622 |  | C | T | DOCK1 | 6 | NM_001290223.1 | R1664C |
| Dp02 | chr10 | 129202619 |  | G | A | DOCK1 | 6 | NM_001290223.1 | V1350I |
| Dg02 | chr6 | 17602895 |  | A | G | FAM8A1 | 6 | NM_016255.2 | I263V |
| Dg13 | chr6 | 17602826 |  | G | T | FAM8A1 | 6 | NM_016255.2 | E240* |
| Dg13 | chr6 | 17602839 |  | C | T | FAM8A1 | 6 | NM_016255.2 | P244L |
| Dg13 | chr6 | 17602863 |  | C | T | FAM8A1 | 6 | NM_016255.2 | A252V |
| Dg18 | chr6 | 17602826 |  | G | T | FAM8A1 | 6 | NM_016255.2 | E240* |
| Dg18 | chr6 | 17602839 |  | C | T | FAM8A1 | 6 | NM_016255.2 | P244L |
| Dg19 | chr6 | 17602826 |  | G | T | FAM8A1 | 6 | NM_016255.2 | E240* |
| Dg19 | chr6 | 17602839 |  | C | T | FAM8A1 | 6 | NM_016255.2 | P244L |
| Dg23 | chr6 | 17602895 |  | A | G | FAM8A1 | 6 | NM_016255.2 | I263V |
| Dp02 | chr6 | 17601040 |  | G | A | FAM8A1 | 6 | NM_016255.2 | G134S |
| Dp02 | chr6 | 17601041 |  | G | T | FAM8A1 | 6 | NM_016255.2 | G134V |
| Dp02 | chr6 | 17601058 |  | G | A | FAM8A1 | 6 | NM_016255.2 | A140T |
| Dp02 | chr6 | 17601086 |  | A | G | FAM8A1 | 6 | NM_016255.2 | Q149R |
| Dp02 | chr6 | 17601098 |  | C | T | FAM8A1 | 6 | NM_016255.2 | S153L |
| Dp02 | chr6 | 17601103 |  | G | A | FAM8A1 | 6 | NM_016255.2 | G155S |
| Dg03 | chr12 | 42499837 |  | A | G | GXYLT1 | 6 | NM_001099650.1 | V185A |
| Dg04 | chr12 | 42499701 |  | C | A | GXYLT1 | 6 | NM_001099650.1 | R230S |
| Dg04 | chr12 | 42499711 |  | C | A | GXYLT1 | 6 | NM_001099650.1 | R227L |
| Dg04 | chr12 | 42499714 |  | T | C | GXYLT1 | 6 | NM_001099650.1 | N226S |
| Dg04 | chr12 | 42499738 |  | T | C | GXYLT1 | 6 | NM_001099650.1 | E218G |
| Dg04 | chr12 | 42499739 |  | C | T | GXYLT1 | 6 | NM_001099650.1 | E218K |
| Dg04 | chr12 | 42499763 |  | T | C | GXYLT1 | 6 | NM_001099650.1 | I210V |
| Dg06 | chr12 | 42499763 |  | T | C | GXYLT1 | 6 | NM_001099650.1 | I210V |
| Dg10 | chr12 | 42538340 |  | C | A | GXYLT1 | 6 | NM_001099650.1 | G37C |
| Dg11 | chr12 | 42499711 |  | C | A | GXYLT1 | 6 | NM_001099650.1 | R227L |
| Dg11 | chr12 | 42499714 |  | T | C | GXYLT1 | 6 | NM_001099650.1 | N226S |
| Dg11 | chr12 | 42499738 |  | T | C | GXYLT1 | 6 | NM_001099650.1 | E218G |
| Dg11 | chr12 | 42499739 |  | C | T | GXYLT1 | 6 | NM_001099650.1 | E218K |
| Dg11 | chr12 | 42499763 |  | T | C | GXYLT1 | 6 | NM_001099650.1 | I210V |
| Dg19 | chr12 | 42538334 |  | C | A | GXYLT1 | 6 | NM_001099650.1 | G39W |
| Dg19 | chr12 | 42538349 |  | T | C | GXYLT1 | 6 | NM_001099650.1 | T34A |
| Dg19 | chr12 | 42538352 |  | C | A | GXYLT1 | 6 | NM_001099650.1 | G33* |
| Dg19 | chr12 | 42538366 |  | A | T | GXYLT1 | 6 | NM_001099650.1 | V28E |
| Dg19 | chr12 | 42538367 |  | C | T | GXYLT1 | 6 | NM_001099650.1 | V28M |
| Dg05 | chr16 | 70913208 |  | G | A | HYDIN | 6 | NM_001270974.1 | P3517S |
| Dg20 | chr16 | 70937823 |  | T | G | HYDIN | 6 | NM_001270974.1 | K2887N |
| Dg20 | chr16 | 71052077 |  | T | C | HYDIN | 6 | NM_001270974.1 | K1200R |
| Dg24 | chr16 | 70908799 |  | C | A | HYDIN | 6 | NM_001270974.1 | E3527D |
| Dg25 | chr16 | 71103184 |  | A | C | HYDIN | 6 | NM_001198542.1 | F681V |
| Dp03 | chr16 | 70841658 |  | C | T | HYDIN | 6 | NM_001270974.1 | R5064H |
| Dp03 | chr16 | 70937945 |  | C | T | HYDIN | 6 | NM_001270974.1 | G2847S |
| Dp04 | chr16 | 71015396 |  | T | G | HYDIN | 6 | NM_001270974.1 | S1470R |
| Dg13 | chr2 | 70315228 |  | G | A | PCBP1 | 6 | NM_006196.3 | C118Y |
| Dg14 | chr2 | 70315757 |  | A | G | PCBP1 | 6 | NM_006196.3 | I294M |
| Dg18 | chr2 | 70314964 |  | G | A | PCBP1 | 6 | NM_006196.3 | G30E |
| Dg20 | chr2 | 70315129 |  | G | A | PCBP1 | 6 | NM_006196.3 | S85N |
| Dg20 | chr2 | 70315398 |  | C | T | PCBP1 | 6 | NM_006196.3 | Q175* |
| Dg20 | chr2 | 70315628 |  | G | A | PCBP1 | 6 | NM_006196.3 | M251I |
| Dg20 | chr2 | 70315712 |  | A | C | PCBP1 | 6 | NM_006196.3 | Q279H |
| Dg21 | chr2 | 70315754 |  | C | G | PCBP1 | 6 | NM_006196.3 | C293W |
| Dg25 | chr2 | 70315642 |  | G | A | PCBP1 | 6 | NM_006196.3 | G256E |
| Dg02 | chr1 | 16262471 |  | A | C | SPEN | 6 | NM_015001.2 | T3246P |
| Dg06 | chr1 | 16237662 |  | T | A | SPEN | 6 | NM_015001.2 | I370K |
| Dg08 | chr1 | 16265843 |  | T | C | SPEN | 6 | NM_015001.2 | L3639P |
| Dg14 | chr1 | 16258916 |  | G | T | SPEN | 6 | NM_015001.2 | E2061* |
| Dg20 | chr1 | 16258893 |  | A | AC | SPEN | 6 | NM_015001.2 | N2053N? |
| Dp03 | chr1 | 16256528 |  | C | T | SPEN | 6 | NM_015001.2 | R1265* |
| Dg01 | chr1 | 2488138 |  | G | A | TNFRSF14 | 6 | NM_001297605.1 | W12* |
| Dg06 | chr1 | 2489782 |  | G | C | TNFRSF14 | 6 | NM_001297605.1 | G60A |
| Dg14 | chr1 | 2488171 |  | T | G | TNFRSF14 | 6 | NM_001297605.1 | L23R |
| Dg23 | chr1 | 2491320 |  | C | A | TNFRSF14 | 6 | NM_001297605.1 | C121* |
| Dp01 | chr1 | 2488105 |  | T | C | TNFRSF14 | 6 | NM_001297605.1 | M1T |
| Dp04 | chr1 | 2488105 |  | T | C | TNFRSF14 | 6 | NM_001297605.1 | M1T |
| Dg12 | chr18 | 60985721 |  | G | A | BCL2 | 5 | NM_000657.2 | A60V |
| Dg12 | chr18 | 60985881 |  | T | C | BCL2 | 5 | NM_000657.2 | T7A |
| Dg13 | chr18 | 60985896 |  | C | G | BCL2 | 5 | NM_000657.2 | A2P |
| Dp01 | chr18 | 60985697 |  | C | T | BCL2 | 5 | NM_000657.2 | R68K |
| Dp07 | chr18 | 60985542 |  | G | T | BCL2 | 5 | NM_000657.2 | H120N |
| Dp09 | chr18 | 60985514 |  | C | T | BCL2 | 5 | NM_000657.2 | R129H |
| Dg07 | chr7 | 2978320 |  | C | T | CARD11 | 5 | NM_032415.5 | R337Q |
| Dg09 | chr7 | 2985468 |  | A | T | CARD11 | 5 | NM_032415.5 | F115I |
| Dg13 | chr7 | 2956981 |  | GTCGCTTGTTGAAAGCGCTTCT | G | CARD11 | 5 | NM_032415.5 | EEALSTSD875D |
| Dg15 | chr7 | 2979491 |  | T | A | CARD11 | 5 | NM_032415.5 | K252N |
| Dg15 | chr7 | 2984162 |  | C | T | CARD11 | 5 | NM_032415.5 | G123D |
| Dg20 | chr7 | 2983885 |  | C | G | CARD11 | 5 | NM_032415.5 | K215N |
| Dg09 | chr19 | 1037670 |  | A | G | CNN2 | 5 | NM_004368.2 | K234R |
| Dg09 | chr19 | 1037679 |  | C | T | CNN2 | 5 | NM_004368.2 | T237I |
| Dg11 | chr19 | 1037670 |  | A | G | CNN2 | 5 | NM_004368.2 | K234R |
| Dg11 | chr19 | 1037679 |  | C | T | CNN2 | 5 | NM_004368.2 | T237I |
| Dg11 | chr19 | 1037681 |  | G | A | CNN2 | 5 | NM_004368.2 | D238N |
| Dg11 | chr19 | 1037715 |  | T | C | CNN2 | 5 | NM_004368.2 | M249T |
| Dg12 | chr19 | 1037670 |  | A | G | CNN2 | 5 | NM_004368.2 | K234R |
| Dg12 | chr19 | 1037679 |  | C | T | CNN2 | 5 | NM_004368.2 | T237I |
| Dg12 | chr19 | 1037715 |  | T | C | CNN2 | 5 | NM_004368.2 | M249T |
| Dp02 | chr19 | 1037640 |  | C | T | CNN2 | 5 | NM_004368.2 | P224L |
| Dp07 | chr19 | 1037756 |  | G | A | CNN2 | 5 | NM_004368.2 | G263S |
| Dp07 | chr19 | 1037766 |  | G | A | CNN2 | 5 | NM_004368.2 | R266Q |
| Dp07 | chr19 | 1037781 |  | C | A | CNN2 | 5 | NM_004368.2 | P271H |
| Dg12 | chr16 | 3786691 |  | A | G | CREBBP | 5 | NM_001079846.1 | L1469P |
| Dg13 | chr16 | 3788605 |  | T | C | CREBBP | 5 | NM_001079846.1 | Y1412C |
| Dg17 | chr16 | 3788618 |  | G | A | CREBBP | 5 | NM_001079846.1 | R1408C |
| Dp07 | chr16 | 3807921 |  | GAGCC | G | CREBBP | 5 | NM_001079846.1 | WL1127 |
| Dp07 | chr16 | 3808045 |  | TA | T | CREBBP | 5 | NM_001079846.1 | Y1087 |
| Dp09 | chr16 | 3817738 |  | G | T | CREBBP | 5 | NM_001079846.1 | S1040* |
| Dp09 | chr16 | 3823813 |  | G | C | CREBBP | 5 | NM_001079846.1 | S763* |
| Dg07 | chr8 | 113353769 |  | T | G | CSMD3 | 5 | NM_052900.2 | T2093P |
| Dg19 | chr8 | 113518989 |  | G | A | CSMD3 | 5 | NM_052900.2 | P1505L |
| Dg20 | chr8 | 113314129 |  | C | A | CSMD3 | 5 | NM_052900.2 | G2609V |
| Dg20 | chr8 | 114326836 |  | T | A | CSMD3 | 5 | NM_052900.2 | Y122F |
| Dg25 | chr8 | 113702210 |  | GA | G | CSMD3 | 5 | NM_052900.2 | S577 |
| Dp07 | chr8 | 114111064 |  | A | C | CSMD3 | 5 | NM_052900.2 | S280A |
| Dg01 | chrX | 41204477 |  | T | C | DDX3X | 5 | NM_001193416.1 | F357S |
| Dg01 | chrX | 41204720 |  | T | C | DDX3X | 5 | NM_001193416.1 | S412P |
| Dg01 | chrX | 41204751 |  | T | A | DDX3X | 5 | NM_001193416.1 | V422E |
| Dg20 | chrX | 41202585 |  | G | C | DDX3X | 5 | NM_001193416.1 | L220F |
| Dg20 | chrX | 41202992 |  | T | C | DDX3X | 5 | NM_001193416.1 | S228P |
| Dg20 | chrX | 41205523 |  | G | T | DDX3X | 5 | NM_001193416.1 | G453C |
| Dg20 | chrX | 41205605 |  | G | C | DDX3X | 5 | NM_001193416.1 | R480T |
| Dp06 | chrX | 41200744 |  | CGATAAAGACAGTT | C | DDX3X | 5 | NM_001193416.1 | DKDSS54 |
| Dp07 | chrX | 41204676 |  | T | G | DDX3X | 5 | NM_001193416.1 | L397* |
| Dp09 | chrX | 41193529 |  | T | A | DDX3X | 5 | NM_001193416.1 | N8K |
| Dg02 | chr2 | 186671591 |  | A | C | FSIP2 | 5 | NM_173651.2 | N5942T |
| Dg07 | chr2 | 186659265 |  | A | C | FSIP2 | 5 | NM_173651.2 | I2557L |
| Dg15 | chr2 | 186657521 |  | A | C | FSIP2 | 5 | NM_173651.2 | K1975N |
| Dg24 | chr2 | 186655879 |  | A | G | FSIP2 | 5 | NM_173651.2 | K1428R |
| Dg25 | chr2 | 186692910 |  | A | G | FSIP2 | 5 | NM_173651.2 | H6923R |
| Dg08 | chr5 | 89925267 |  | A | AT | GPR98 | 5 | NM_032119.3 | I584I? |
| Dg24 | chr5 | 89924569 |  | C | A | GPR98 | 5 | NM_032119.3 | P477T |
| Dp02 | chr5 | 90107113 |  | G | C | GPR98 | 5 | NM_032119.3 | D5346H |
| Dp04 | chr5 | 89949311 |  | T | G | GPR98 | 5 | NM_032119.3 | I1307S |
| Dp07 | chr5 | 89925037 |  | A | C | GPR98 | 5 | NM_032119.3 | Y507S |
| Dg02 | chr6 | 26234915 |  | G | C | HIST1H1D | 5 | NM_005320.2 | L83V |
| Dg03 | chr6 | 26235010 |  | G | A | HIST1H1D | 5 | NM_005320.2 | A51V |
| Dg13 | chr6 | 26234902 |  | C | G | HIST1H1D | 5 | NM_005320.2 | S87T |
| Dg20 | chr6 | 26234623 |  | G | C | HIST1H1D | 5 | NM_005320.2 | T180R |
| Dp01 | chr6 | 26234590 |  | G | C | HIST1H1D | 5 | NM_005320.2 | A191G |
| Dp01 | chr6 | 26234932 |  | T | G | HIST1H1D | 5 | NM_005320.2 | N77T |
| Dg07 | chr6 | 26123917 |  | C | G | HIST1H2BC | 5 | NM_003526.2 | E72D |
| Dg09 | chr6 | 26123753 |  | T | G | HIST1H2BC | 5 | NM_003526.2 | *127S |
| Dg18 | chr6 | 26123805 |  | G | A | HIST1H2BC | 5 | NM_003526.2 | H110Y |
| Dg25 | chr6 | 26123805 |  | G | T | HIST1H2BC | 5 | NM_003526.2 | H110N |
| Dg25 | chr6 | 26123994 |  | T | C | HIST1H2BC | 5 | NM_003526.2 | K47E |
| Dp01 | chr6 | 26123916 |  | G | A | HIST1H2BC | 5 | NM_003526.2 | R73C |
| Dg05 | chr6 | 31323175 |  | C | T | HLA-B | 5 | NM_005514.6.3 | V272M |
| Dg08 | chr6 | 31323967 |  | C | T | HLA-B | 5 | NM_005514.6.3 | G199E |
| Dg16 | chr6 | 31323154 |  | G | A | HLA-B | 5 | NM_005514.6.3 | Q279* |
| Dg25 | chr6 | 31324484 |  | G | C | HLA-B | 5 | NM_005514.6.3 | Y108* |
| Dg26 | chr6 | 31323133 |  | G | A | HLA-B | 5 | NM_005514.6.3 | Q286* |
| Dg26 | chr6 | 31324579 |  | C | G | HLA-B | 5 | NM_005514.6.3 | E77Q |
| Dg26 | chr6 | 31324659 |  | C | G | HLA-B | 5 | NM_005514.6.3 | G50A |
| Dg01 | chr6 | 32551970 |  | T | A | HLA-DRB1 | 5 | NM_002124.3 | I96F |
| Dg01 | chr6 | 32552132 |  | T | C | HLA-DRB1 | 5 | NM_002124.3 | R42G |
| Dg01 | chr6 | 32552138 |  | G | A | HLA-DRB1 | 5 | NM_002124.3 | P40S |
| Dg01 | chr6 | 32552139 |  | C | G | HLA-DRB1 | 5 | NM_002124.3 | Q39H |
| Dg05 | chr6 | 32552085 |  | G | T | HLA-DRB1 | 5 | NM_002124.3 | D57E |
| Dg15 | chr6 | 32552132 |  | T | C | HLA-DRB1 | 5 | NM_002124.3 | R42G |
| Dg15 | chr6 | 32552138 |  | G | A | HLA-DRB1 | 5 | NM_002124.3 | P40S |
| Dg15 | chr6 | 32552139 |  | C | G | HLA-DRB1 | 5 | NM_002124.3 | Q39H |
| Dg24 | chr6 | 32552087 |  | C | A | HLA-DRB1 | 5 | NM_002124.3 | D57Y |
| Dp06 | chr6 | 32551991 |  | A | G | HLA-DRB1 | 5 | NM_002124.3 | Y89H |
| Dg01 | chr6 | 32489934 |  | C | A | HLA-DRB5 | 5 | NM_002125.3 | D40Y |
| Dg01 | chr6 | 32497960 |  | C | A | HLA-DRB5 | 5 | NM_002125.3 | K14N |
| Dg01 | chr6 | 32497961 |  | T | A | HLA-DRB5 | 5 | NM_002125.3 | K14M |
| Dg01 | chr6 | 32497970 |  | T | C | HLA-DRB5 | 5 | NM_002125.3 | Y11C |
| Dg01 | chr6 | 32497988 |  | T | C | HLA-DRB5 | 5 | NM_002125.3 | K5R |
| Dg04 | chr6 | 32497988 |  | T | C | HLA-DRB5 | 5 | NM_002125.3 | K5R |
| Dg05 | chr6 | 32489889 |  | A | T | HLA-DRB5 | 5 | NM_002125.3 | F55I |
| Dg05 | chr6 | 32489892 |  | G | A | HLA-DRB5 | 5 | NM_002125.3 | R54W |
| Dg05 | chr6 | 32489927 |  | T | G | HLA-DRB5 | 5 | NM_002125.3 | Y42S |
| Dg15 | chr6 | 32489856 |  | C | T | HLA-DRB5 | 5 | NM_002125.3 | D66N |
| Dg15 | chr6 | 32489856 |  | C | A | HLA-DRB5 | 5 | NM_002125.3 | D66Y |
| Dg15 | chr6 | 32489877 |  | C | A | HLA-DRB5 | 5 | NM_002125.3 | D59Y |
| Dg18 | chr6 | 32489844 |  | C | T | HLA-DRB5 | 5 | NM_002125.3 | D70N |
| Dg03 | chr12 | 10588530 |  | C | G | KLRC2 | 5 | NM_002260.3 | R19P |
| Dg10 | chr12 | 10588530 |  | C | G | KLRC2 | 5 | NM_002260.3 | R19P |
| Dg16 | chr12 | 10588530 |  | C | G | KLRC2 | 5 | NM_002260.3 | R19P |
| Dg20 | chr12 | 10588530 |  | C | G | KLRC2 | 5 | NM_002260.3 | R19P |
| Dp02 | chr12 | 10588530 |  | C | G | KLRC2 | 5 | NM_002260.3 | R19P |
| Dg09 | chr2 | 141460110 |  | C | A | LRP1B | 5 | NM_018557.2 | W2012C |
| Dg09 | chr2 | 141460112 |  | A | T | LRP1B | 5 | NM_018557.2 | W2012R |
| Dg16 | chr2 | 141253233 |  | T | G | LRP1B | 5 | NM_018557.2 | S2979R |
| Dg18 | chr2 | 141208171 |  | G | GTCATCC | LRP1B | 5 | NM_018557.2 | D3341EDD |
| Dg20 | chr2 | 141143479 |  | G | A | LRP1B | 5 | NM_018557.2 | S3505L |
| Dg20 | chr2 | 141143558 |  | G | T | LRP1B | 5 | NM_018557.2 | H3479N |
| Dp04 | chr2 | 141108388 |  | C | A | LRP1B | 5 | NM_018557.2 | R3957M |
| Dg03 | chr8 | 128750543 |  | A | C | MYC | 5 | NM_002467.4 | Y27S |
| Dg14 | chr8 | 128748864 |  | A | G | MYC | 5 | NM_002467.4 | N9D |
| Dg15 | chr8 | 128750612 |  | A | T | MYC | 5 | NM_002467.4 | Q50L |
| Dg15 | chr8 | 128750613 |  | G | T | MYC | 5 | NM_002467.4 | Q50H |
| Dg15 | chr8 | 128750614 |  | C | G | MYC | 5 | NM_002467.4 | Q51E |
| Dg15 | chr8 | 128750616 |  | G | C | MYC | 5 | NM_002467.4 | Q51H |
| Dg15 | chr8 | 128750625 |  | G | C | MYC | 5 | NM_002467.4 | E54D |
| Dg15 | chr8 | 128751064 |  | T | C | MYC | 5 | NM_002467.4 | S201P |
| Dp04 | chr8 | 128750525 |  | G | A | MYC | 5 | NM_002467.4 | S21N |
| Dp07 | chr8 | 128750680 |  | A | C | MYC | 5 | NM_002467.4 | T73P |
| Dg03 | chr12 | 78592387 |  | C | A | NAV3 | 5 | NM_001024383.1 | P2150Q |
| Dg15 | chr12 | 78574802 |  | A | G | NAV3 | 5 | NM_001024383.1 | N1890S |
| Dp04 | chr12 | 78511876 |  | C | A | NAV3 | 5 | NM_001024383.1 | P947T |
| Dp08 | chr12 | 78534083 |  | TG | T | NAV3 | 5 | NM_001024383.1 | L1551 |
| Dp09 | chr12 | 78513539 |  | G | A | NAV3 | 5 | NM_001024383.1 | R1188H |
| Dg01 | chr1 | 228452089 |  | G | T | OBSCN | 5 | NM_001098623.2 | A1620S |
| Dg06 | chr1 | 228467060 |  | G | T | OBSCN | 5 | NM_001098623.2 | M2437I |
| Dg17 | chr1 | 228491398 |  | A | C | OBSCN | 5 | NM_001271223.2 | E4587D |
| Dg17 | chr1 | 228562120 |  | G | A | OBSCN | 5 | NM_001098623.2 | S7497N |
| Dg20 | chr1 | 228469819 |  | G | C | OBSCN | 5 | NM_001098623.2 | V2795L |
| Dg20 | chr1 | 228504652 |  | G | A | OBSCN | 5 | NM_001098623.2 | D4510N |
| Dg25 | chr1 | 228509137 |  | A | C | OBSCN | 5 | NM_001098623.2 | K4865N |
| Dg08 | chr8 | 52733242 |  | C | T | PCMTD1 | 5 | NM_001286782.1 | R172H |
| Dg15 | chr8 | 52732958 |  | G | A | PCMTD1 | 5 | NM_001286782.1 | L267F |
| Dg19 | chr8 | 52732961 |  | G | A | PCMTD1 | 5 | NM_001286782.1 | P266S |
| Dg20 | chr8 | 52732972 |  | A | AT | PCMTD1 | 5 | NM_001286782.1 | I262N? |
| Dp03 | chr8 | 52732972 |  | A | AT | PCMTD1 | 5 | NM_001286782.1 | I262N? |
| Dg07 | chr6 | 106547220 |  | G | T | PRDM1 | 5 | NM_001198.3 | E153* |
| Dg10 | chr6 | 106553398 |  | AG | A | PRDM1 | 5 | NM_001198.3 | S455 |
| Dg14 | chr6 | 106547339 |  | CAACCAGGAA | C | PRDM1 | 5 | NM_001198.3 | NQE193 |
| Dg14 | chr6 | 106547349 |  | C | T | PRDM1 | 5 | NM_001198.3 | L196F |
| Dg26 | chr6 | 106553774 |  | G | A | PRDM1 | 5 | NM_001198.3 | C580Y |
| Dp02 | chr6 | 106547305 |  | AC | A | PRDM1 | 5 | NM_001198.3 | N181 |
| Dg03 | chr1 | 237797002 |  | T | G | RYR2 | 5 | NM_001035.2 | V2227G |
| Dg13 | chr1 | 237758925 |  | G | T | RYR2 | 5 | NM_001035.2 | A1522S |
| Dg17 | chr1 | 237619906 |  | A | C | RYR2 | 5 | NM_001035.2 | I495L |
| Dg17 | chr1 | 237955458 |  | T | G | RYR2 | 5 | NM_001035.2 | S4539R |
| Dg20 | chr1 | 237965207 |  | C | G | RYR2 | 5 | NM_001035.2 | F4714L |
| Dg26 | chr1 | 237791126 |  | T | G | RYR2 | 5 | NM_001035.2 | I2062M |
| Dg26 | chr1 | 237868544 |  | G | T | RYR2 | 5 | NM_001035.2 | A3161S |
| Dg26 | chr1 | 237982460 |  | T | G | RYR2 | 5 | NM_001035.2 | F4853C |
| Dg04 | chr12 | 122242657 |  | A | AC | SETD1B | 5 | NM_015048.1 | H5H? |
| Dg07 | chr12 | 122248137 |  | G | A | SETD1B | 5 | NM_015048.1 | R429Q |
| Dg13 | chr12 | 122242657 |  | A | AC | SETD1B | 5 | NM_015048.1 | H5H? |
| Dg26 | chr12 | 122243813 |  | G | T | SETD1B | 5 | NM_015048.1 | E116* |
| Dp03 | chr12 | 122260618 |  | C | T | SETD1B | 5 | NM_015048.1 | T1335M |
| Dg01 | chr16 | 11348766 |  | G | C | SOCS1 | 5 | NM_003745.1 | N190K |
| Dg01 | chr16 | 11348808 |  | C | G | SOCS1 | 5 | NM_003745.1 | E176D |
| Dg01 | chr16 | 11348945 |  | G | C | SOCS1 | 5 | NM_003745.1 | Q131E |
| Dg01 | chr16 | 11348989 |  | C | G | SOCS1 | 5 | NM_003745.1 | S116T |
| Dg01 | chr16 | 11349096 |  | G | C | SOCS1 | 5 | NM_003745.1 | Y80* |
| Dg01 | chr16 | 11349176 |  | G | A | SOCS1 | 5 | NM_003745.1 | H54Y |
| Dg01 | chr16 | 11349307 |  | T | C | SOCS1 | 5 | NM_003745.1 | D10G |
| Dg02 | chr16 | 11349290 |  | C | A | SOCS1 | 5 | NM_003745.1 | A16S |
| Dg02 | chr16 | 11349322 |  | T | C | SOCS1 | 5 | NM_003745.1 | N5S |
| Dg20 | chr16 | 11348711 |  | A | G | SOCS1 | 5 | NM_003745.1 | F209L |
| Dg20 | chr16 | 11348721 |  | G | C | SOCS1 | 5 | NM_003745.1 | S205R |
| Dg20 | chr16 | 11348853 |  | C | T | SOCS1 | 5 | NM_003745.1 | M161I |
| Dg20 | chr16 | 11348945 |  | G | C | SOCS1 | 5 | NM_003745.1 | Q131E |
| Dg20 | chr16 | 11348962 |  | C | G | SOCS1 | 5 | NM_003745.1 | S125T |
| Dg20 | chr16 | 11348972 |  | C | T | SOCS1 | 5 | NM_003745.1 | G122R |
| Dg20 | chr16 | 11349004 |  | C | T | SOCS1 | 5 | NM_003745.1 | C111Y |
| Dg20 | chr16 | 11349082 |  | C | T | SOCS1 | 5 | NM_003745.1 | S85N |
| Dg20 | chr16 | 11349095 |  | A | C | SOCS1 | 5 | NM_003745.1 | W81G |
| Dg20 | chr16 | 11349155 |  | G | C | SOCS1 | 5 | NM_003745.1 | H61D |
| Dg20 | chr16 | 11349287 |  | C | T | SOCS1 | 5 | NM_003745.1 | A17T |
| Dg20 | chr16 | 11349289 |  | G | A | SOCS1 | 5 | NM_003745.1 | A16V |
| Dg20 | chr16 | 11349329 |  | C | T | SOCS1 | 5 | NM_003745.1 | A3T |
| Dg20 | chr16 | 11349332 |  | C | G | SOCS1 | 5 | NM_003745.1 | V2L |
| Dg20 | chr16 | 11349333 |  | C | T | SOCS1 | 5 | NM_003745.1 | M1I |
| Dg25 | chr16 | 11349332 |  | C | T | SOCS1 | 5 | NM_003745.1 | V2I |
| Dp01 | chr16 | 11348989 |  | C | T | SOCS1 | 5 | NM_003745.1 | S116N |
| Dg18 | chrX | 24382453 |  | G | C | SUPT20HL1 | 5 | NM_001136234.1 | A526P |
| Dg21 | chrX | 24382453 |  | G | C | SUPT20HL1 | 5 | NM_001136234.1 | A526P |
| Dg22 | chrX | 24382495 |  | G | C | SUPT20HL1 | 5 | NM_001136234.1 | A540P |
| Dg22 | chrX | 24382501 |  | G | C | SUPT20HL1 | 5 | NM_001136234.1 | A542P |
| Dp01 | chrX | 24382501 |  | G | GCTGCTCCTGCTC | SUPT20HL1 | 5 | NM_001136234.1 | A542AAPAP |
| Dp03 | chrX | 24382429 |  | C | G | SUPT20HL1 | 5 | NM_001136234.1 | P518A |
| Dg01 | chr6 | 32821566 |  | A | T | TAP1 | 5 | NM_000593.5.7 | S10T |
| Dg08 | chr6 | 32821130 |  | G | GCAGC | TAP1 | 5 | NM_000593.5.7 | A155GC? |
| Dg13 | chr6 | 32821269 |  | G | A | TAP1 | 5 | NM_000593.5.7 | P109S |
| Dg16 | chr6 | 32820881 |  | C | T | TAP1 | 5 | NM_000593.5.7 | G238D |
| Dp01 | chr6 | 32818776 |  | A | C | TAP1 | 5 | NM_000593.5.7 | L392R |
| Dg03 | chr3 | 176763923 |  | G | T | TBL1XR1 | 5 | NM_024665.4 | H307N |
| Dg03 | chr3 | 176763924 |  | A | T | TBL1XR1 | 5 | NM_024665.4 | F306L |
| Dg03 | chr3 | 176763925 |  | A | T | TBL1XR1 | 5 | NM_024665.4 | F306Y |
| Dg03 | chr3 | 176763926 |  | A | T | TBL1XR1 | 5 | NM_024665.4 | F306I |
| Dg18 | chr3 | 176755954 |  | C | A | TBL1XR1 | 5 | NM_024665.4 | V352L |
| Dg26 | chr3 | 176765152 |  | C | A | TBL1XR1 | 5 | NM_024665.4 | G267V |
| Dp02 | chr3 | 176750787 |  | T | A | TBL1XR1 | 5 | NM_024665.4 | D463V |
| Dp08 | chr3 | 176769487 |  | T | A | TBL1XR1 | 5 | NM_024665.4 | I78L |
| Dg01 | chr4 | 106156335 |  | TC | T | TET2 | 5 | NM_001127208.2 | P413 |
| Dg01 | chr4 | 106197035 |  | C | CT | TET2 | 5 | NM_001127208.2 | L1790L? |
| Dg14 | chr4 | 106155920 |  | TC | T | TET2 | 5 | NM_001127208.2 | I274 |
| Dg23 | chr4 | 106158444 |  | T | TA | TET2 | 5 | NM_001127208.2 | I1116N? |
| Dp04 | chr4 | 106193931 |  | C | T | TET2 | 5 | NM_001127208.2 | R1465* |
| Dp05 | chr4 | 106164895 |  | TA | T | TET2 | 5 | NM_001127208.2 | Y1255 |
| Dp05 | chr4 | 106164897 |  | C | CGGAAATTGGT | TET2 | 5 | NM_001127208.2 | G1256GNWW? |
| Dg06 | chr7 | 88964410 |  | T | G | ZNF804B | 5 | NM_181646.2 | L705W |
| Dg19 | chr7 | 88965640 |  | A | C | ZNF804B | 5 | NM_181646.2 | N1115T |
| Dg20 | chr7 | 88956731 |  | A | C | ZNF804B | 5 | NM_181646.2 | Q108P |
| Dp03 | chr7 | 88963260 |  | G | A | ZNF804B | 5 | NM_181646.2 | A322T |
| Dp05 | chr7 | 88964703 |  | C | T | ZNF804B | 5 | NM_181646.2 | H803Y |

Supplementary Table 5: Analysis of *TP53* gene copy number variation

| Case no. | *TP53* Ct | *GAPDH* Ct | ΔCt | ΔΔCt | Ratio | CN | CN |
| --- | --- | --- | --- | --- | --- | --- | --- |
| V01 | 24.49 | 22.64 | 1.85 | 0.07 | 0.96 | 1.91 | 2 |
| V02 | 24.10 | 22.30 | 1.80 | 0.01 | 0.99 | 1.98 | 2 |
| V03 | 24.55 | 22.42 | 2.13 | 0.34 | 0.79 | 1.57 | 2 |
| V04 | 24.33 | 22.20 | 2.13 | 0.34 | 0.79 | 1.58 | 2 |
| V05 | 24.68 | 22.80 | 1.88 | 0.09 | 0.94 | 1.88 | 2 |
| V06 | 24.65 | 22.56 | 2.10 | 0.31 | 0.81 | 1.61 | 2 |
| V07 | 24.60 | 22.94 | 1.66 | -0.13 | 1.09 | 2.19 | 2 |
| V08 | 24.29 | 22.38 | 1.91 | 0.12 | 0.92 | 1.84 | 2 |
| V09 | 23.89 | 22.06 | 1.84 | 0.05 | 0.97 | 1.94 | 2 |
| V10 | 24.45 | 22.65 | 1.80 | 0.01 | 0.99 | 1.99 | 2 |
| V11 | 23.62 | 22.03 | 1.59 | -0.20 | 1.15 | 2.29 | 2 |
| V12 | 23.94 | 22.15 | 1.78 | -0.01 | 1.01 | 2.01 | 2 |
| V13 | 24.08 | 22.48 | 1.60 | -0.19 | 1.14 | 2.28 | 2 |
| V14 | 24.86 | 23.38 | 1.48 | -0.31 | 1.24 | 2.47 | 2 |
| V15 | 23.83 | 21.98 | 1.84 | 0.06 | 0.96 | 1.92 | 2 |
| V16 | 24.26 | 22.49 | 1.77 | -0.02 | 1.01 | 2.03 | 2 |
| V17 | 23.88 | 22.49 | 1.38 | -0.41 | 1.33 | 2.65 | 2 |
| V18 | 24.45 | 22.83 | 1.62 | -0.17 | 1.12 | 2.25 | 2 |
| V19 | 24.42 | 22.50 | 1.91 | 0.13 | 0.92 | 1.83 | 2 |
| V20 | 24.18 | 22.04 | 2.14 | 0.35 | 0.78 | 1.57 | 2 |
| V21 | 25.27 | 22.76 | 2.51 | 0.72 | 0.61 | 1.21 | 1 |
| V22 | 24.82 | 23.08 | 1.73 | -0.05 | 1.04 | 2.08 | 2 |
| V23 | 24.34 | 22.61 | 1.74 | -0.05 | 1.04 | 2.08 | 2 |
| V24 | 24.90 | 23.08 | 1.82 | 0.03 | 0.98 | 1.96 | 2 |
| V25 | 24.75 | 22.50 | 2.24 | 0.45 | 0.73 | 1.46 | 2 |
| V26 | 24.39 | 22.17 | 2.22 | 0.43 | 0.74 | 1.49 | 2 |
| V27 | 24.33 | 22.42 | 1.91 | 0.12 | 0.92 | 1.84 | 2 |
| V28 | 24.03 | 21.67 | 2.36 | 0.57 | 0.67 | 1.34 | 2 |
| V29 | 24.53 | 22.73 | 1.81 | 0.02 | 0.99 | 1.98 | 2 |
| V30 | 24.17 | 22.43 | 1.74 | -0.05 | 1.03 | 2.07 | 2 |
| V31 | 24.71 | 22.96 | 1.75 | -0.04 | 1.03 | 2.05 | 2 |
| V32 | 24.46 | 22.51 | 1.95 | 0.16 | 0.89 | 1.79 | 2 |
| V33 | 24.67 | 22.21 | 2.46 | 0.67 | 0.63 | 1.26 | 1 |
| V34 | 24.46 | 22.53 | 1.92 | 0.13 | 0.91 | 1.82 | 2 |
| V35 | 24.21 | 22.08 | 2.13 | 0.34 | 0.79 | 1.58 | 2 |
| V36 | 24.37 | 22.55 | 1.82 | 0.03 | 0.98 | 1.96 | 2 |
| V37 | 24.85 | 22.81 | 2.04 | 0.25 | 0.84 | 1.68 | 2 |
| V38 | 23.88 | 21.62 | 2.25 | 0.47 | 0.72 | 1.45 | 2 |
| V39 | 24.13 | 22.39 | 1.74 | -0.05 | 1.04 | 2.07 | 2 |
| V40 | 24.77 | 22.57 | 2.20 | 0.42 | 0.75 | 1.50 | 2 |
| V41 | 24.27 | 22.34 | 1.94 | 0.15 | 0.90 | 1.81 | 2 |
| V42 | 24.14 | 22.51 | 1.63 | -0.16 | 1.12 | 2.24 | 2 |
| V43 | 24.29 | 22.16 | 2.14 | 0.35 | 0.79 | 1.57 | 2 |
| V44 | 24.06 | 21.78 | 2.28 | 0.49 | 0.71 | 1.42 | 2 |
| V45 | 24.27 | 22.49 | 1.78 | -0.01 | 1.01 | 2.02 | 2 |
| V46 | 24.44 | 22.43 | 2.02 | 0.23 | 0.85 | 1.71 | 2 |
| V47 | 24.31 | 21.93 | 2.38 | 0.59 | 0.66 | 1.32 | 1 |
| V48 | 23.68 | 21.54 | 2.14 | 0.35 | 0.78 | 1.57 | 2 |
| V49 | 24.25 | 22.38 | 1.87 | 0.08 | 0.95 | 1.89 | 2 |
| V50 | 24.40 | 22.25 | 2.16 | 0.37 | 0.77 | 1.55 | 2 |
| V51 | 25.13 | 22.49 | 2.64 | 0.85 | 0.56 | 1.11 | 1 |
| V52 | 24.51 | 22.52 | 1.99 | 0.20 | 0.87 | 1.74 | 2 |
| V53 | 24.28 | 22.13 | 2.15 | 0.36 | 0.78 | 1.56 | 2 |
| V54 | 24.64 | 22.39 | 2.25 | 0.46 | 0.73 | 1.45 | 2 |
| V55 | 23.89 | 21.64 | 2.25 | 0.47 | 0.72 | 1.45 | 2 |
| V56 | 24.51 | 22.30 | 2.21 | 0.42 | 0.75 | 1.49 | 2 |
| V57 | 24.98 | 22.54 | 2.44 | 0.65 | 0.64 | 1.27 | 1 |
| V58 | 24.20 | 22.02 | 2.18 | 0.39 | 0.76 | 1.53 | 2 |
| V59 | 23.88 | 22.09 | 1.79 | 0.00 | 1.00 | 2.00 | 2 |
| V60 | 24.65 | 22.19 | 2.45 | 0.66 | 0.63 | 1.26 | 1 |
| V61 | 24.69 | 22.71 | 1.98 | 0.20 | 0.87 | 1.75 | 2 |
| V62 | 24.13 | 22.22 | 1.91 | 0.12 | 0.92 | 1.84 | 2 |
| V63 | 25.21 | 22.15 | 3.07 | 1.28 | 0.41 | 0.82 | 1 |
| V64 | 24.39 | 22.29 | 2.09 | 0.31 | 0.81 | 1.62 | 2 |
| V65 | 24.31 | 22.66 | 1.65 | -0.14 | 1.10 | 2.21 | 2 |
| V66 | 24.21 | 22.23 | 1.99 | 0.20 | 0.87 | 1.74 | 2 |
| V67 | 25.26 | 22.51 | 2.75 | 0.96 | 0.51 | 1.03 | 1 |
| V68 | 25.04 | 22.71 | 2.33 | 0.54 | 0.69 | 1.38 | 2 |
| V69 | 24.86 | 22.92 | 1.94 | 0.15 | 0.90 | 1.80 | 2 |
| V70 | 24.22 | 22.33 | 1.89 | 0.10 | 0.93 | 1.87 | 2 |
| V71 | 24.01 | 22.35 | 1.66 | -0.13 | 1.09 | 2.19 | 2 |
| V72 | 25.01 | 22.76 | 2.25 | 0.46 | 0.73 | 1.45 | 2 |
| V73 | 24.47 | 22.36 | 2.10 | 0.32 | 0.80 | 1.61 | 2 |
| V74 | 24.27 | 22.62 | 1.66 | -0.13 | 1.10 | 2.19 | 2 |
| V75 | 24.65 | 22.96 | 1.69 | -0.10 | 1.07 | 2.14 | 2 |
| V76 | 25.09 | 22.38 | 2.71 | 0.92 | 0.53 | 1.06 | 1 |
| V77 | 25.21 | 22.58 | 2.63 | 0.84 | 0.56 | 1.12 | 1 |
| V78 | 24.17 | 22.44 | 1.72 | -0.07 | 1.05 | 2.09 | 2 |
| V79 | 24.89 | 22.46 | 2.43 | 0.64 | 0.64 | 1.28 | 1 |
| V80 | 24.73 | 22.09 | 2.64 | 0.85 | 0.55 | 1.11 | 1 |
| V81 | 25.64 | 23.49 | 2.15 | 0.36 | 0.78 | 1.56 | 2 |
| V82 | 24.27 | 22.20 | 2.07 | 0.28 | 0.82 | 1.64 | 2 |
| V83 | 24.92 | 22.40 | 2.52 | 0.73 | 0.60 | 1.21 | 1 |
| V84 | 24.29 | 22.26 | 2.03 | 0.24 | 0.84 | 1.69 | 2 |
| V85 | 24.35 | 22.65 | 1.70 | -0.09 | 1.07 | 2.13 | 2 |

Supplementary Table 7: Confirmation by direct sequencing

| Case no. | CHR | POS | REF | ALT | refGene_Gene | refGene_No | refGene_AACH | Forward primer | Reverse primer | Confirmation by direct sequencing |
| --- | --- | --- | --- | --- | --- | --- | --- | --- | --- | --- |
| Dg26 | chr3 | 32022391 | C | T | OSBPL10 | NM_001174060.1 | R94K | ATCACTGGGTTCGCTGAAGG | CATTTCCCGGGGATTTGGAG | Yes |
| Dg26 | chr3 | 32022413 | G | A | OSBPL10 | NM_001174060.1 | L87F | ATCACTGGGTTCGCTGAAGG | CATTTCCCGGGGATTTGGAG | Yes |
| Dg26 | chr3 | 32022422 | A | T | OSBPL10 | NM_001174060.1 | Y84N | ATCACTGGGTTCGCTGAAGG | CATTTCCCGGGGATTTGGAG | Yes |
| Dg26 | chr3 | 32022426 | G | C | OSBPL10 | NM_001174060.1 | S82R | ATCACTGGGTTCGCTGAAGG | CATTTCCCGGGGATTTGGAG | Yes |
| Dg02 | chr3 | 32022427 | C | G | OSBPL10 | NM_001174060.1 | S82T | ATCACTGGGTTCGCTGAAGG | CATTTCCCGGGGATTTGGAG | Yes |
| Dg26 | chr3 | 32022431 | G | C | OSBPL10 | NM_001174060.1 | L81V | ATCACTGGGTTCGCTGAAGG | CATTTCCCGGGGATTTGGAG | Yes |
| Dg18 | chr3 | 32022500 | GGCTGCTGCGGCTT | G | OSBPL10 | NM_001174060.1 | GSRSS53 | ATCACTGGGTTCGCTGAAGG | CATTTCCCGGGGATTTGGAG | Yes |
| Dg10 | chr3 | 32022510 | G | T | OSBPL10 | NM_001174060.1 | S54R | ATCACTGGGTTCGCTGAAGG | CATTTCCCGGGGATTTGGAG | Yes |
| Dg13 | chr3 | 32022601 | G | A | OSBPL10 | NM_001174060.1 | A24V | ATCACTGGGTTCGCTGAAGG | CATTTCCCGGGGATTTGGAG | Yes |
| Dg18 | chr3 | 32022602 | C | T | OSBPL10 | NM_001174060.1 | A24T | ATCACTGGGTTCGCTGAAGG | CATTTCCCGGGGATTTGGAG | Yes |
| Dg02 | chr3 | 32022608 | T | A | OSBPL10 | NM_001174060.1 | S22C | ATCACTGGGTTCGCTGAAGG | CATTTCCCGGGGATTTGGAG | Yes |
| Dg17 | chr3 | 32022622 | C | T | OSBPL10 | NM_001174060.1 | S17N | ATCACTGGGTTCGCTGAAGG | CATTTCCCGGGGATTTGGAG | Yes |
| Dg06 | chr3 | 32022625 | C | T | OSBPL10 | NM_001174060.1 | S16N | ATCACTGGGTTCGCTGAAGG | CATTTCCCGGGGATTTGGAG | Yes |
| Dg20 | chr3 | 32022625 | C | T | OSBPL10 | NM_001174060.1 | S16N | ATCACTGGGTTCGCTGAAGG | CATTTCCCGGGGATTTGGAG | Yes |
| Dg26 | chr3 | 32022625 | C | T | OSBPL10 | NM_001174060.1 | S16N | ATCACTGGGTTCGCTGAAGG | CATTTCCCGGGGATTTGGAG | Yes |
| Dg09 | chr3 | 32022635 | C | T | OSBPL10 | NM_001174060.1 | G13S | ATCACTGGGTTCGCTGAAGG | CATTTCCCGGGGATTTGGAG | Yes |
| Dg20 | chr17 | 7577085 | C | T | TP53 | NM_000546.5 | E285K | AGAGGAGCTGGTGTTGTTGG | TTGGGAGTAGATGGAGCCTG | Yes |
| Dg20 | chr17 | 7577097 | C | G | TP53 | NM_000546.5 | D281H | AGAGGAGCTGGTGTTGTTGG | TTGGGAGTAGATGGAGCCTG | Yes |
| Dp02 | chr17 | 7577105 | G | A | TP53 | NM_000546.5 | P278L | AGAGGAGCTGGTGTTGTTGG | TTGGGAGTAGATGGAGCCTG | Yes |
| Dp08 | chr17 | 7577561 | ACT | A | TP53 | NM_000546.5 | S240 | GCCAGAGAAAAGAAAACTGAGTG | CCCCATGAGATGTGCAAAGT | Yes |
| Dg01 | chr17 | 7578190 | T | C | TP53 | NM_000546.5 | Y220C | ATTTACTTTGCACATCTCATGGG | CACTTGTGCCCTGACTTTCA | Yes |
| Dp05 | chr17 | 7578203 | C | T | TP53 | NM_000546.5 | V216M | ATTTACTTTGCACATCTCATGGG | CACTTGTGCCCTGACTTTCA | Yes |
| Dg19 | chr17 | 7578388 | C | T | TP53 | NM_000546.5 | R181H | ATTTACTTTGCACATCTCATGGG | CACTTGTGCCCTGACTTTCA | Yes |
| Dp04 | chr17 | 7578407 | G | C | TP53 | NM_000546.5 | R175G | ATTTACTTTGCACATCTCATGGG | CACTTGTGCCCTGACTTTCA | Yes |
| Dp01 | chr17 | 7578429 | CTG | C | TP53 | NM_000546.5 | Q167 | ATTTACTTTGCACATCTCATGGG | CACTTGTGCCCTGACTTTCA | Yes |
